# iSODA: A Comprehensive Tool for Integrative Omics Data Analysis in Single- and Multi-Omics Experiments

**DOI:** 10.1021/acs.analchem.4c04355

**Published:** 2025-01-31

**Authors:** Damien Olivier-Jimenez, Rico J. E. Derks, Oscar Harari, Carlos Cruchaga, Muhammad Ali, Alessandro Ori, Domenico Di Fraia, Birol Cabukusta, Andy Henrie, Martin Giera, Yassene Mohammed

**Affiliations:** † Center for Proteomics and Metabolomics, Leiden University Medical Center, Leiden 2333ZA, Netherlands; ‡ Department of Neurology, The Ohio State University, Columbus, Ohio 43210, United States of America; § Washington University School of Medicine in St. Louis, St. Louis, Missouri 63110, United States of America; ∥ Leibniz Institute on Aging—Fritz Lipmann Institute (FLI), Jena 07745, Germany; ⊥ Department of Cell and Chemical Biology, ONCODE Institute, Leiden University Medical Center, Leiden 2333ZA, Netherlands; # DataTecnica, Washington, District of Columbia 20037, United States of America; ¶ Gerald Bronfman Department of Oncology, McGill University, Montreal, Quebec H3A 0G4, Canada

## Abstract

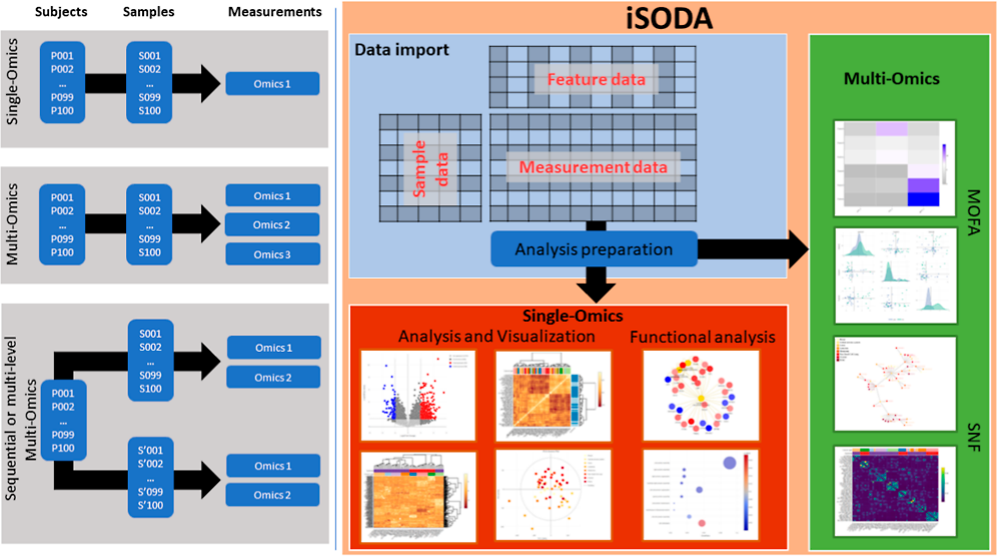

Thanks to the plummeting costs of continuously evolving omics analytical platforms, research centers collect multiomics data more routinely. They are, however, confronted with the lack of a versatile software solution to harmoniously analyze single-omics and interpret multiomics data. We have developed iSODA, a web-based application for the analysis of single- and multiomics data. The tool emphasizes intuitive interactive visualizations designed for user-driven data exploration. Researchers can access a variety of functions ranging from simple visualization like volcano plots and PCA to advanced functional analyses like enrichment analysis and lipid saturation analysis. For integrated multiomics, iSODA incorporates multi-omics factor analysis and similarity network fusion. The ability to adapt the data on-the-fly allows for tasks, such as the removal of outlier samples or failed features, imputation, or normalization. All results are presented through interactive plots, the modular design supports extensions, and tooltips and tutorials provide comprehensive guidance for users. iSODA is accessible under http://isoda.online/.

## Introduction

The rapid advances in high-throughput omics resulted in an increase in data generation at lower costs.^1^ These data help understanding biological processes, elucidating disease pathways, and advancing personalized medicine.^2^ Various tools and methods exist to navigate omics data—from statistical techniques pinpointing significant patterns to network-based approaches highlighting relationships within biological systems. Most tools, however, require either advanced knowledge of coding for data handling or are dedicated to a specific omics platform.

A few solutions dedicated to analysis of (multi)omics data were developed over the past decade. Some focus on specific diseases or applications, while others adopt a general approach.^3−6^ In the long list of software tools (Table S1), a few notable implementations stand out for their interactive design and generality. MetaboAnalyst is a web-based application for metabolomics data analysis and interpretation.^7^ It allows for streamlined analysis for both quantitative and untargeted metabolomics data. Perseus is specialized in interpreting protein quantification, interaction, and post-translational modification data.^8^ It enables omics data analysis covering normalization, pattern recognition, time series analysis, and classification. PaintOmics 4 maps acquired omics data on biological pathways,^6^ including KEGG^9^ and Reactome.^10^ MergeOmics is a pipeline for integrating data sets across omics.^11^ It leverages three functional analyses for disease association; marker set enrichment analysis (MSEA), meta-MSEA, and key driver analysis. OmicsAnalyst focuses on multiomics analysis through three tracks; correlation network, cluster heatmap, and dimension reduction.^4^ 3Omics generates interomics correlation networks with respect to experimental conditions for transcriptomics, proteomics, and metabolomics.^5^ It includes coexpression analysis, phenotype analysis for transcriptomics and proteomics, KEGG pathway EA for metabolomics and proteomics, and Gene Ontology EA for proteomics and transcriptomics. In summary, great advances have been made toward convergent multiomics data analysis. However, most tools take a deterministic approach to data analysis, making it a linear pipeline rather than exploration. The tools are either specialized for a specific omics or dedicated to multiomics but not both. Static plotting also seems to be common, in contrast to interactivity. All of these aspects limit the users’ experience and ability to explore the data dynamically.

We have developed integrated simple omics data analysis (iSODA) as an interactive software solution for single- and multiomics data exploration. iSODA is a web-based platform that offers users a dynamic environment to process, study, and integrate their data; essentially to study multiomics characterization experiments, on each omics layer individually, and side-by-side with multiomics integration. Our goal was to transcend the linear approach to data analysis to enable a dynamic exploration. We demonstrate iSODA by using two applications. The first is a lipidomics library of 90 lipid transport protein knockouts studying the effect of these proteins on the cell lipidome.^12^ In the second, we use the multiomics characterization data sets of the NCI-60 cell lines.^13^ We discuss how to use iSODA to pinpoint insights in these data sets.

## Materials and Methods

### Software Implementation

iSODA was developed in R—4.4.0 and Shiny framework.^14^ We used bs4Dash and ShinyWidgets for UI elements, shinymanager for authentication, and shinyjs and shinybrowser for aspect ratio adjustment. The backend architecture implements three R6 classes; (1) the omics class for single omics experiments; (2) the multi-omics factor analysis (MOFA) class for multi omics factor analysis;^15^ and (3) the similarity network fusion (SNF) class for SNF integration.^16^ We used plotly^17^ for interactive plots, visNework for visualizing networks, and heatmaply for heatmaps.^18^ We used RColorBrewer, viridisLite, and grDevices to provide various color palettes. Omics data are imported as tables using the data.table package, and RDS compressed files are used to save results.

The various analyses rely on the stats core package, including: distance calculations—Euclidean, maximum, Manhattan, Canberra, binary, and Minkowski. Hierarchical clustering methods—ward.D, ward.D2, single, complete, average, McQuitty, median, and centroid. Pearson’s and Spearman’s correlation coefficients for correlation, Student’s *t* test and Mann–Whitney–Wilcoxon for statistical tests, and *p*-value adjustment methods include Bonferroni, Holm, Hochberg, Hommel, Benjamini & Hochberg, and Benjamini & Yekutieli correction. Principal component analysis—PCA using pcaMethods package.^19^ Advanced regression analysis and feature selection was achieved using Lasso and Elastic-Net Regularized Generalized Linear Models using the glmnet package.^20^ For functional analyses, we used ClusterProfiler and Enrichplot packages for enrichment and over-representation analyses.^21^ Gene and protein annotations are provided using the Org.Hs.eg.db package,^22^ and users can also upload their own annotation for any omics for functional analyses.

### LTP KO Lipidomics Library

The lipid transfer protein knockout (LTP KO) library was produced by our group^12^ to investigate the disturbances caused by the loss of LTPs. Briefly, the MelJuSo cell line [human melanoma (ME)] was used, and 90 LTP gene knockout experiments were conducted using CRISPR/Cas9 technology (Table S2). These 90 genes are from 11 families determined by the LTP domains: oxysterol-binding protein (OSBP) (*n* = 30), START (*n* = 34), PITP (*n* = 16), GLTP (*n* = 11), CRAL-TRIO (*n* = 71), SMP (*n* = 15), VPS13 (*n* = 11), NPC1 NTD (*n* = 6), ML (*n* = 9), SCP2 (*n* = 12), and ASTER/VAST (*n* = 8). Additionally, six nontargeting controls were included. These samples were analyzed for their lipidomics content in the original study.^12^

### NCI-60 Cancer Cell Lines Multiomics Data Set

The 60 Human Tumor Cell Lines used by the National Cancer Institute for over 20 years as a screen to identify and characterize novel compounds for growth inhibition or killing of tumor cell lines.^23^ The screen utilizes 60 different human tumor cell lines, representing leukemia (LE, *n* = 6), ME (*n* = 10), and cancers of breast (BR, *n* = 5), central nervous system/brain (CNS, *n* = 6), colon (CO, *n* = 7), nonsmall cell lung (LC, *n* = 9), ovarian (OV, *n* = 7), prostate (PR, *n* = 2), and renal (RE, *n* = 8). Table S3 lists the individual cell lines. CellMiner is an online database hosting the multiomics characterization data on the NCI-60 cell lines.^13^ We used three characterization data sets representing genomics (Illumina 450 K methylation, gene average), transcriptomics (5 Platform Gene Transcript, averaged intensities), and proteomics (SWATH mass spectrometry, protein). A complete description of the sample measurement and data acquisition are provided in the original works.^13^

## Results

iSODA has interfaces for single-omics and for multiomics integration (Figure S1). Tooltips provide concise descriptions throughout the interface. Results can be saved, uploaded, and shared via compressed downloadable files or kept on the server using UUIDs. We describe next iSODA briefly and refer to the Supporting Information for details.

### Single-Omics Analysis

Data can be uploaded using three tables: sample annotations, measurement data, and feature annotations. Data preanalysis, includes filtering, normalization (scaling, total normalization, and standardization), imputation (minimum, median, mean, and maximum), and batch effect correction using ComBat.^24^ In interactive visualization up to four analyses can be displayed simultaneously. The analyses include dendrograms, PCA, volcano plots, heatmaps, and feature/sample correlation analyses. Visualization and analysis parameters can be adjusted for each plot from its sidebar. All plots and their data can be downloaded. iSODA allows omics specific analyses, like lipid class distribution and double bonds plots, for lipidomics.

Functional analysis implements EA and over-representation analysis (ORA). EA is based on K–S statistics as implemented originally by Geneset EA.^25^ ORA uses the Fisher exact test to determine if a feature annotation is over-represented.^26^ The user can upload their own feature annotations, while gene ontology terms are made readily available for the corresponding omics, i.e., genomics, transcriptomics, and proteomics. Results can be shown in dot, bar, ridge, CNET, and eMap plots. We refer to the Supporting Information for a detailed description and highlight here CNET—an interactive network of features and enriched terms. CNET allows clustering nodes dynamically using physics laws, either pulling together or repelling nodes based on their associations.

### Multiomics Integration

Integration provides a holistic view to pinpoint patterns that are not observable in the single-omics. Currently, iSODA supports MOFA and SNF. MOFA is an unsupervised integration that reduces large-scale omics data sets into latent factors.^15^ These factors capture the underlying sources of variation across the omics data sets, providing insights into driving biological processes. MOFA’s factors are similar to PCA components in one omics layer. Results can be explored through explained variance, factor, combined factors, feature weights, feature top weights, and scatter plots as well as MOFA heatmap. SNF constructs individual networks for each omics data set representing the similarity between samples. These networks are then fused into a single network through an iterative process capturing both the shared and unique characteristics of each data set.^16^ Results can be explored using similarity heatmap and network, as well as fusion heatmap and network.

### Lipidomics of LTP KOs

LTPs are classified into families based on protein domains suggesting similarities in function.^27^ There are currently 348 proteins annotated as lipid transport proteins.^28^ To systematically study and highlight the functions of various LTPs, we characterized 90 intracellular LTP KO based on the MelJuSo cell line using targeted quantitative lipidomics with internal standards (Table S2 and Figure S2).^12^ Using iSODA, we imputed missing values with a median and removed lipid species below two times the average blank signals; 752 lipid species out of the measured 1109 remained and were used for further analysis.

**Figure 1:**
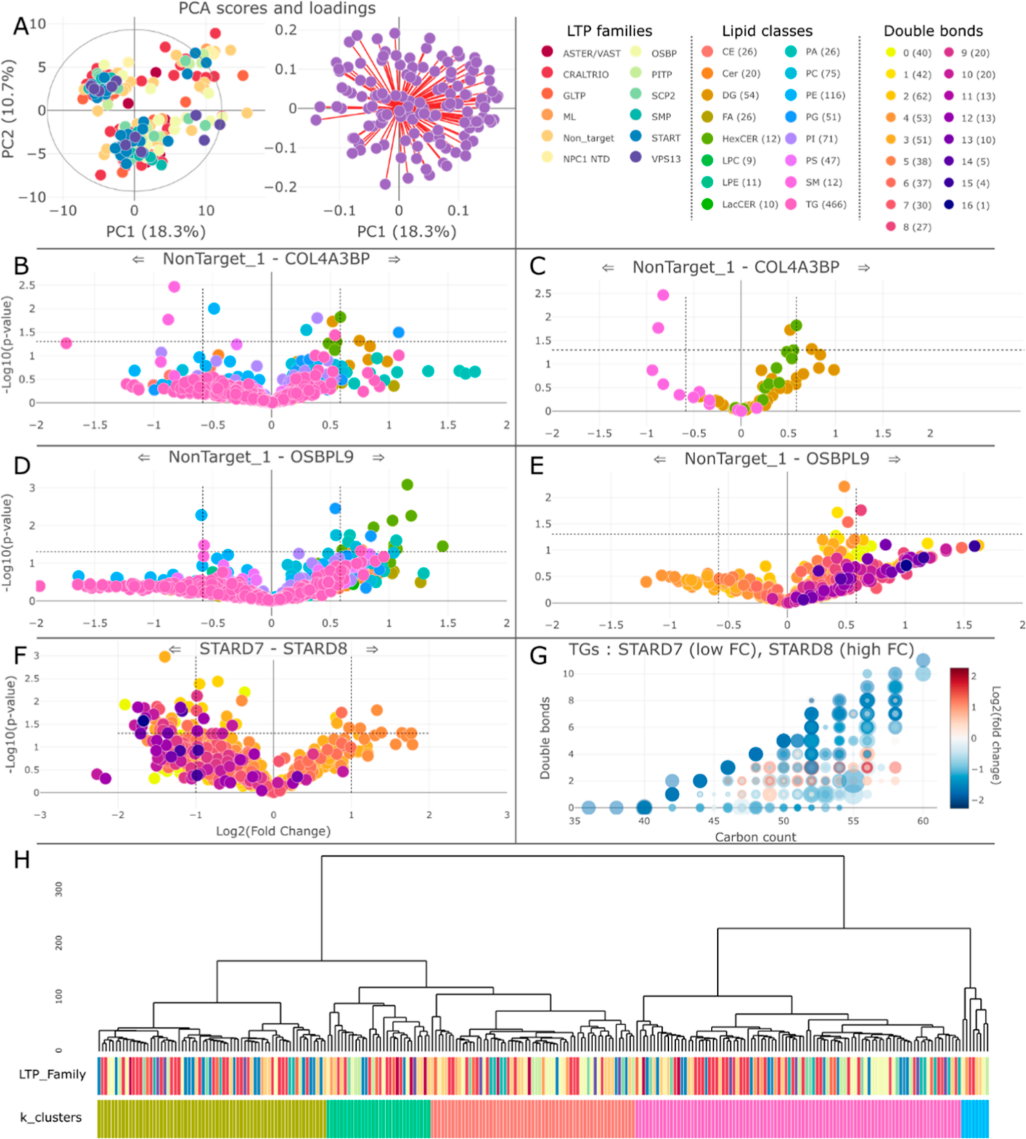
Lipidomics analysis of the LTP KO data set. (A) PCA of all KO. (B) Volcano plot comparing COL4A3BP KO to nontarget and (C) selected lipid classes with trends are displayed. (D) Comparing OSBPL9 to nontarget showing no visible trend for any class and (E) with only TGs colored by the total double bond count showing a clear trend. In (F,G), TGs in StarD7 and StarD8 were compared showing different trends regarding saturation. (H) Dendrogram of all LTP KO library using all lipid species measured showing no obvious alignment with the LTP family, samples could, however, be clustered according to 5 main groups. Total normalized tables were used, lasso was applied to PCA based on the LTP family, alpha = 0.8.

First, we attempted to identify any association between the LTP families and the observed lipidomics phenotype. The protein family is based on the functional domain similarity and suggests that similar functions would be reflected in respective lipid cargo giving rise to similar patterns in lipidomics, underlining associations between LTP families and their role in lipid metabolism. Our data revealed no such associations (Figure 1A). Considering that a protein domain might interact/affect specific lipid species, and therefore, the effect might only show on a subset of measured lipids, we performed a multinomial LASSO regression to extract any discriminatory lipid species between families. This also led to no observable discrimination (Figure 1A). It can hence be argued that protein domains and their similarities are not necessarily proxies for the actual function. Examination on a gene-by-gene basis seemed essential to identify the effects of a specific knockout on the lipidome (Table S4).

We highlight here a few examples. COL4A3BP, also known as ceramide transfer protein, primarily transfers ceramides between the endoplasmic reticulum and Golgi apparatus. Ceramides are key components of cell membranes and play a crucial role in cellular signaling and apoptosis. Dysregulation of ceramide metabolism has been linked to various cancers. Moreover, given the importance of sphingolipids in the nervous system, dysfunction of COL4A3BP is known to be implicated in neurological diseases.^29^ When comparing the COL4A3BP KO to control (Figure 1B), a few significant lipid species can be detected. Mapping the lipid class on the volcano plot revealed that DGs and LacCer species were present at higher concentrations, while lipids from the SM class were at lower concentrations (Figure 1C). Although not at a significant level with a FC of 1.5 (corresponding to logFC cutoff 0.585), a trend can be seen given all measured LacCer and SM species.

Next, we investigated oxysterol binding protein-like 9, OSBPL9, which is part of the OSBP family. It contains a pleckstrin homology domain, which facilitates binding to phosphoinositides, and a conserved oxysterol-binding domain that binds sterols and other lipids and therefore is believed to bind and transport predominantly sterols and phosphoinositides within cells. OSBPL9 plays a crucial role in maintaining cellular lipid homeostasis and is involved in various cell signaling pathways that regulate growth, survival, and metabolism. Disruptions in OSBPL9 may contribute to neurological disorders given how critical lipid homeostasis for neural functions is. Several lipid classes show overproduction in the OSBPL9 knockout compared to the control, notably hexosylceramides (HexCer) with multiple significant species (Figure 1D). When focusing specifically on triglycerides (TG) and investigating the double bonds, additional patterns emerge with a count of double bonds higher than 5 being gradually overexpressed in the knockouts (Figure 1E).

StarD7 and StarD8 belong to the StAR-related lipid transfer (START) domain family, known for their roles in lipid binding and transfer. StarD7 is specifically involved in the intracellular transport of phospholipids and, particularly, the transfer of phosphatidylcholine to mitochondria, which is crucial for maintaining mitochondrial membrane integrity and function. StarD8, also known as DLC-3 (deleted in liver cancer-3), is involved in lipid binding and transport and plays a role in cellular signaling pathways by interacting with phosphoinositides. StarD8 is also known to regulate the activity of Rho family GTPases, which are critical for cytoskeletal dynamics, cell movement, and growth. Comparing the effects of the StarD7 and StarD8 knockouts on the cell lipidome shows that they have opposite effects within a single lipid class. The absence of StarD7 affects TG saturation, while StarD8’s absence does not (Figures 1F and S3). This effect can also be seen in more detail when using the double bond plots, specifically showing opposite effects for many of the lipid species examined (Figures 1G and S3).

### Multiomics of NCI-60 Cancer Cell Lines

The three omics data sets we used covered epigenomics, transcriptomics, and proteomics. After inspection, prostate cancer with only two cell lines was under-represented and removed. Two other samples were excluded, i.e., ME:MDA-N for having no data in proteomics and CNS:SF-539 for having too many missing values in transcriptomics. The remaining data covered 8 cancers; LE, ME, breast, CNS/brain, colon, nonsmall cell lung, ovarian, and renal.

#### Single-Omics Analyses

**Figure 2:**
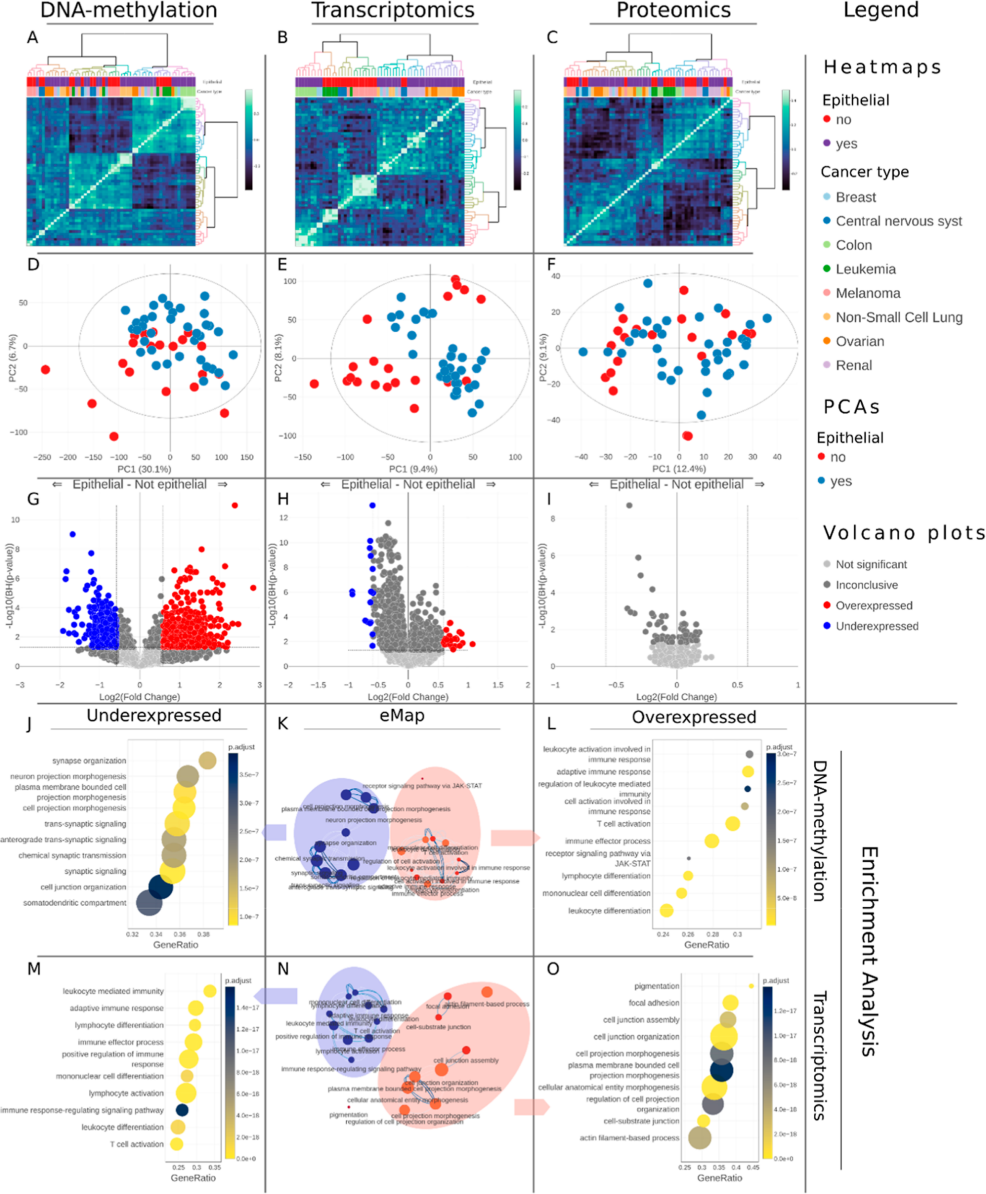
Single-omics and functional analyses of the NCI-60 DNA-methylation, transcriptomics, and proteomics data. (A–C) Correlation heatmaps showing similar clusters based on Pearson’s correlation. (D–F) PCA score plots (*z*-scored total normalized data, the nipals PCA method) colored by epithelial nature. (G–I) Volcano plots comparing cell lines according to their epithelial nature (total normalized data, *t* test, FC using mean, B–H *p*-value adjustment, *p*-value threshold was set to 0.05, FC threshold was set to 1.5). (J–O) Functional analyses with results for DNA-methylation and transcriptomics data comparing LE and ME cell lines. Feature set adjustment was set to BH, *p*-value = 0.05, set minimum, and maximum size were 3 and 800, respectively. (J,L) are dot plots and K is the eMap from the DNA-methylation data, M–O are for transcriptomics. In dot plots top 10 enriched terms were selected, in eMaps top 20, colored by NES values with blue for negative and red for positive, the node size was scaled to gene count, similarity score was JC, and score threshold was 0.2. For each omics, the suppressed (J,M) and activated (L,O) biological processes and molecular functions are shown.

The sample correlation heatmap (Figure 2A–C) shows that the clusters follow the cancer type in transcriptomics (Figure 2B), but not on proteomics and DNA-methylation level (Figure 2A,C). A closer look shows that the epithelial nature of the cells, i.e., carcinoma or not, is a main driver of grouping. This is most apparent in transcriptomics, which seems to carry the most embedded patterns regarding cancer and the epithelial nature. PCA score plots of both cancer (Figure S4D–F) and epithelial nature (Figure 2D–F) show similar trends. Here, the non-epithelial cell lines form two groups with LE on top-right and ME together with CNS at bottom-left (Figure 2E). Epithelial cell lines show distinction between the colon on the one side, and lung, ovarian, and renal cancer on the other side. A regression analysis to select discriminating features can be applied with the provided sample annotations. When using cancer types as a grouping end point, various features in each of the three omics were identified (Figure S4J–L). Interestingly, the epithelial nature of the cell lines still drove the clustering, indicating an underlying strong similarity between cell lines based on origin. A similar analysis regarding the epithelial nature was also performed (Figure S5).

To identify statistically significant features, we used volcano plots, and epithelial and non-epithelial cell lines were compared in all three omics. Many discriminating genes were identified in DNA-methylation data, with fewer in transcriptomics, and no significant proteins were found (Figure 2G–I). While the multivariate analysis using regression was able to identify features for such discrimination, the univariate analysis using statistical testing and fold change was less effective, save for DNA-methylation. These results appear to be intertwining; carcinoma seems to be regulated upstream; hence, DNA-methylation data shows discriminating features in volcano plots; however, the effect on the phenotype is more subtle appearing only in the multivariate analysis. We also compared various cell lines in pairs, Figure S4P–R shows LE and ME, both non-epithelial. Although no proteins were significantly over- or under-represented, various transcripts and methylated genes were. The possibility to perform these analyses and interact with the results in a few clicks enables streamlined analysis and quick contrasting toward trend identification.

#### Functional Analysis

In addition to accepting user’s custom annotations for functional analyses, iSODA annotates genomics, transcriptomics, and proteomics with Gene Ontology. From various possible comparisons, we highlight the results of the EA comparing ME to LE (Figure 2J–O). Interestingly, functions related to an immune response were upregulated in the DNA-methylation and downregulated in the transcriptomics data. This aligns with the general understanding that DNA methylation regulates gene transcription through repression. Here, we observe this repression on the functional level rather than on a specific gene. Various functions that exhibit opposite regulation in DNA methylation versus transcriptomics were associated with different immune system pathways. Further, methylation of genes associated with synapses and morphogenesis were reduced in ME, while genes associated with pigmentation and cell junction were upregulated in ME. ORA of the differentiated gene methylation between epithelial and non-epithelial cell lines resulted in functions grouped into two clusters; associated with epidermis development and cell junction assembly (Figure S6A,B). The functional analyses demonstrate how different omics complement each other in describing cell biology. To achieve a more holistic view, we next describe their integration.

#### SNF of the NCI-60 Cell Lines

**Figure 3:**
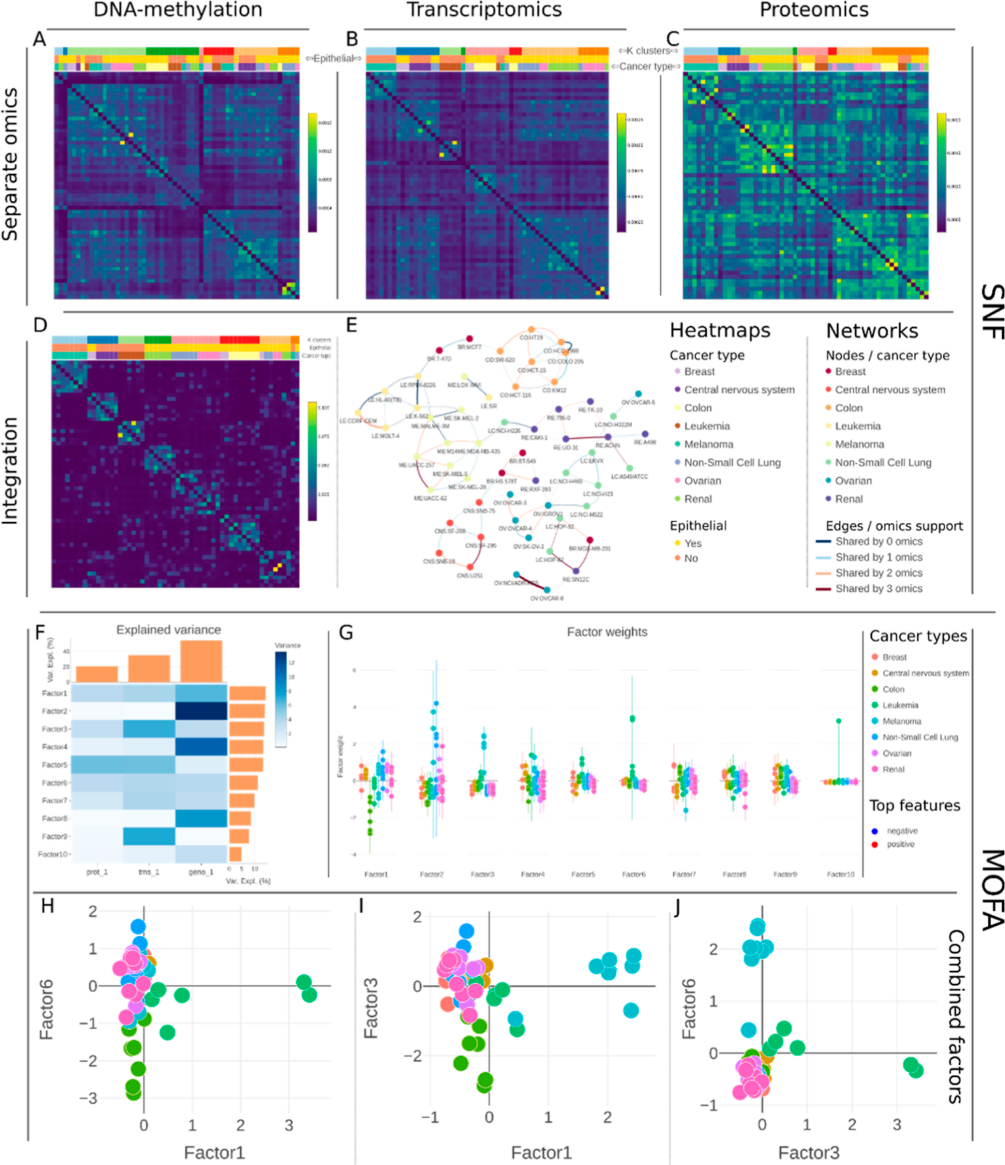
SNF on NCI-60 cell lines. (A–C) Similarity heatmaps. (D,E) Fusion heatmap and network results. *k*-Nearest neighbors method was set to 5, sigma to 0.5, using an Euclidean distance, and setting k clusters to 8. The heatmaps were mapped with *k* clusters, cancer type, and epithelial status, while the networks were colored by the cancer type and 5% of the top scoring edges were kept. Detailed plots are in Figure S7. (F–J) MOFA on DNA-methylation, transcriptomics, and proteomics data sets of the NCI-60 cell lines. F explained variance for each factor and omics combinations. *G* is the factor plot comparing sample weights stratified according to the cancer type. (H–J) Combined factor plots showing how specific groups of interest can be discriminated using a combination of factors; in this case colon, LE, and ME show good discrimination combining factors 1, 3, and 6.

SNF finds similarities between samples using spectral clustering.^16^ For comparison, we show single-omics and multiomics clustering (Figure 3A–E). Before fusion, LE cell lines are barely clustered together, the renal cell lines produce compact clusters on DNA-methylation and transcriptomics, and the ME cell line cluster generally consistently in all three data sets. After SNF, the integrated view better captures the nature of cell lines by clustering these, to a good degree, into their original cancers (Figure 3D,E). A data-driven *k* = 8 clustering shows a better agreement with the original cancer types after SNF. While SNF indicates integration enhances clustering, we turn to MOFA to study features driving the similarities.

#### MOFA of the NCI-60 Cell Lines

We set the MOFA to generate 10 factors; however, the exact number of factors varies for different goals. For example, up to 10 factors are recommended to identify features that drive the variance in the different omics layers, while finding outlier samples requires more factors; details can be found in MOFA documentations.^15^ The variance plot (Figure 3F) shows the explained variance associated with each factor and omics. The factor plot shows the samples’ factor weights and allows coloring according to the different sample annotations. When projecting the epithelial nature of the cell lines, there was no clear separation across all 10 factors. When projecting the cancer type, a few factors stood out, especially factors 1, 3, and 6 separating colon, LE, and ME from the rest (Figure 3G–J). For an in-depth view of how the integration results relate to the data measured, top contributing features for a factor can be displayed (Figure S8A,C) along with the measured values and associated sample weight (Figure S8D–F). Heatmaps of these top features and how they contribute to discrimination in each omics can also be studied (Figure S9).

## Discussion

iSODA allows users to analyze their single-omics experiments individually, and side-by-side use multiomics integration. The unified interface enables an efficient streamlined analysis for multiomics experiments. Although we have demonstrated the functionalities using two examples, the actual emphasis of iSODA lies in its interactive visualization and modular design. The latter open the door for combining methods; for example, EA on lipid saturation. Although EA and double bond annotation are each not novel, the combination results in a novel analysis (example in the Supporting Information). The modular architecture allows also extending the core single-omics module to the specifics of the omics data; for example, handling lipid shorthand IDs.^30^ iSODA’s modularity was employed in SODAlight, a lipidomics-only instance for the Neurolipid Atlas.^31^

When performing the literature search (Table S1), six implementations stood out for providing a graphical user interface, are general purpose, and not specific toward a disease or organism; i.e. MetaboAnalyst,^7^ Perseus,^8^ PaintOmics 4,^6^ MergeOmics,^11^ OmicsAnalyst,^4^ and 3Omics.^5^ We compared the tools based on six criteria (Table 1). For data filtering, PaintOmics, MergeOmics, 3Omics, and OmicsAnalyst do not provide filtering options, requiring users to perform filtering and data preparation externally. MetaboAnalyst, Perseus, and iSODA, offer advanced filtering. Regarding visualization, MetaboAnalyst, Perseus, and iSODA provide interactive plots; however, this interactivity is central in iSODA allowing various advanced plotting. All seven evaluated software packages offer some form of functional analysis; however, iSODA allows users to supply custom annotations. Regarding a unified interface to single-omics analysis and multiomics integration, most omics tools including the ones discussed here can be split into two categories: those specialized in single-omics (e.g., MetaboAnalyst and Perseus) and those for multiomics (e.g., PaintOmics, MergeOmics, 3Omics, and OmicsAnalyst). iSODA provides a unified interface to perform both.

**Table 1:**
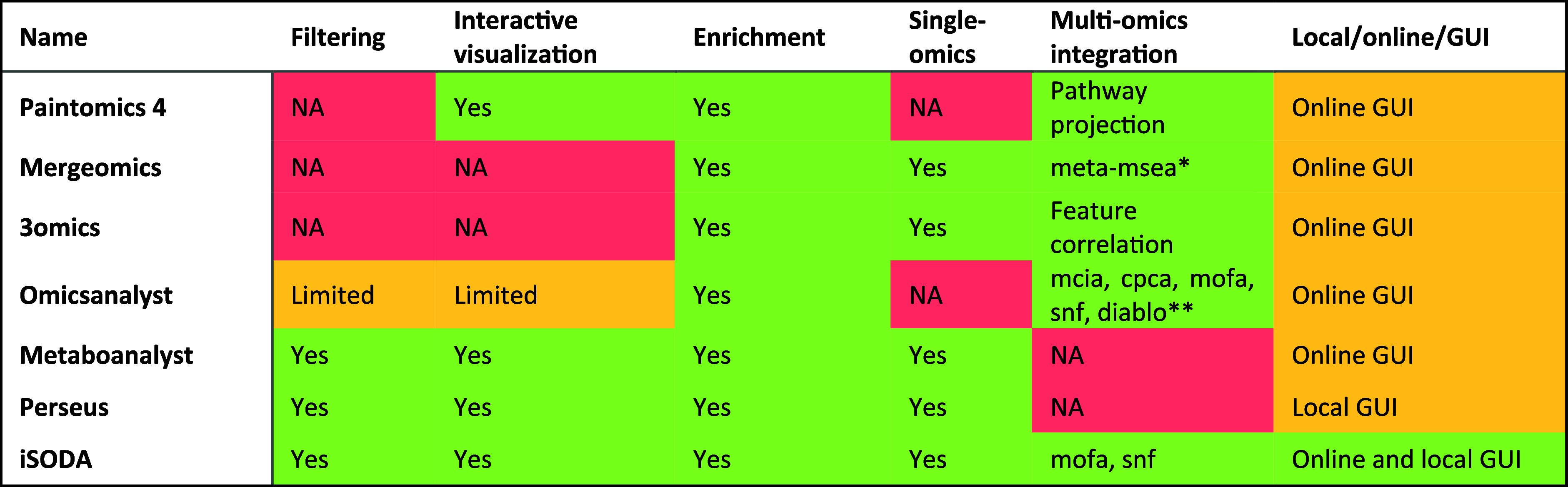
Comparison of 6 Software Packages^a^

a *Meta-MSEA: meta-MSEA. **Many: MCIA (multiple co-inertia analysis), CPCA (consensus PCA), MOFA, SNF, and DIABLO. Tools were selected for having GUI, capability to handle multi-omics, and being non-specific to a disease or organism.

For multiomics integration, a variety of methods are used, ranging from knowledge-driven approaches based on enrichment to data-driven approaches based on variance or similarity. Knowledge-driven methods like pathway projection (PaintOmics 4) or meta-MSEA (MergeOmics) are less effective for small molecules omics, such as lipidomics. OmicsAnalyst offers the most extensive range of data-driven methods, including DIABLO and PLS-DA (supervised) and SNF, MOFA, and CPCA (unsupervised). iSODA currently focuses on two unsupervised methods, SNF and MOFA. However, given the unified interface of iSODA, various methods for the supervised feature selection (like discriminant regression analysis or statistical tests) can be performed in each single-omics modules individually, and the results carried over to the multiomics modules, resulting in a supervised MOFA or SNF.

While data analysis happens on the server side in iSODA, visualization and data upload involve the browser directly. Relying on browser technologies makes tools accessible; however, it comes with performance challenges. We evaluated the performance with the data sets used here and one larger multiomics data set from 363 neuro degenerative disease (NDD) patients. Using a 6-year-old computer at the time of writing (Windows 10, Intel W-2135, 64 GB RAM), we measured reasonable data upload and plot generation times. For the LTP data set (4.19 MB, 350k entries), NCI-60 transcriptomics (8 MB, 1.43 M entries), and NDD transcriptomics (106 MB, 21 M entries), we measured 0.3, 0.47, and 6.78 s for data upload and 0.02, 9.77, and 28.44 s for generating volcano plots, respectively. It is worth mentioning that the latter corresponds to 121,000 data points on the screen. Table S5 summarizes the results.

Although iSODA can be extended, the current release has a few limitations. iSODA was meant to work on the feature level and not raw instrument data; however, it can be extended with processing modules to extract feature tables from raw data. For example, parsing mzML and mzIdentML to generate feature tables (using mzR package^32^) should allow direct import for proteomics data. iSODA manages data sets with 100 s of samples; however, we expect that collections with 1000 or 10,000 s of samples might be a challenging task for visualization. Reduction or stratification of data, which can be performed in iSODA, should allow such analyses in the current version. Extending visualization with modules that use scalable visual summaries^33^ should allow handling larger collections of data points on the screens,^34^ and running the tool on dedicated and better computational resources should also help. For supervised feature selection, iSODA uses mainly LASSO, i.e., there is currently no other supervised data analysis like PLS–DA or SVM implemented; LASSO is a proven versatile method compared to others. Groups comparison is limited to parametric the Student’s *t* test or nonparametric Mann–Whitney–Wilcoxon test; however, additional tests can be included using an existing code as a template. There is currently no automated outlier removal; we believe such a step should be performed manually, and iSODA aids in that. The application meant to live in a trusted environment; hence, there is currently no advanced user and data management logic; however, running iSODA in Posit Connect (commercial alternative to Shiny Server) would readily provide advanced management.

## Conclusions

Interactive data visualization allows finding trends faster, identifying relationships effectively, and simplifying complex data in a stepwise manner. iSODA is a user-friendly interactive data analysis tool that equally focuses on single-omics and multiomics. It includes a core module with several omics-independent processes that can be extended to handle omics-specific analyses and visualizations. The multiomics analyses are linked to the single-omics modules, leveraging the insights gained from single-omics processing to improve the integrative analysis. Importantly, iSODA is not a linear analysis pipeline; it is rather a versatile toolbox to explore data in innovative ways; going back and forth between plots and omics views, filtering, and exploring specific parts of the uploaded data, all with the possibility to download the plot-associated data for possible external analysis. Future developments aim to introduce more specialized processes for each of the five current modules: lipidomics, metabolomics, proteomics, transcriptomics, and genomics, as well as additional multiomics integration strategies.

## Data Availability

iSODA is accessible under http://isoda.online. Data used is available from iSODA Web site as well as GitHub https://github.com/isodaonline/iSODA. The iSODA source code is available under GPL-3.0 license.

## Supporting Information Available

Detailed description of iSODA user interface and available functionalities and interactive visualization, extended figures of the results, list of software tools for omics data analyses, metadata of the samples used, tabular version of the LTP family *k*-nearest neighbors clustering, and comparison of multiomics platforms (PDF)

## References

[ref1] Stephens Z. D.; Lee S. Y.; Faghri F.; Campbell R. H.; Zhai C.; Efron M. J.; Iyer R.; Schatz M. C.; Sinha S.; Robinson G. E. Big Data: Astronomical or Genomical?. PLoS Biol. 2015, 13 (7), e1002195 10.1371/journal.pbio.1002195. 26151137 PMC4494865

[ref2] Hasin Y.; Seldin M.; Lusis A. Multi-omics approaches to disease. Genome Biol. 2017, 18 (1), 83 10.1186/s13059-017-1215-1. 28476144 PMC5418815

[ref3] aConard A. M.; Goodman N.; Hu Y.; Perrimon N.; Singh R.; Lawrence C.; Larschan E. TIMEOR: a web-based tool to uncover temporal regulatory mechanisms from multi-omics data. Nucleic Acids Res. 2021, 49 (W1), W641–W653. 10.1093/nar/gkab384. 34125906 PMC8262710

[ref4] Zhou G.; Ewald J.; Xia J. OmicsAnalyst: a comprehensive web-based platform for visual analytics of multi-omics data. Nucleic Acids Res. 2021, 49 (W1), W476–W482. 10.1093/nar/gkab394. 34019646 PMC8262745

[ref5] Kuo T. C.; Tian T. F.; Tseng Y. J. 3Omics: a web-based systems biology tool for analysis, integration and visualization of human transcriptomic, proteomic and metabolomic data. BMC Syst. Biol. 2013, 7, 64 10.1186/1752-0509-7-64. 23875761 PMC3723580

[ref6] Yin Y.; Liu X. Z.; Tian Q.; Fan Y. X.; Ye Z.; Meng T. Q.; Wei G. H.; Xiong C. L.; Li H. G.; He X.; et al. Transcriptome and DNA methylome analysis of peripheral blood samples reveals incomplete restoration and transposable element activation after 3-months recovery of COVID-19. Front. Cell Dev. Biol. 2022, 10, 1001558 10.3389/fcell.2022.1001558. 36263014 PMC9574079

[ref7] Pang Z.; Zhou G.; Ewald J.; Chang L.; Hacariz O.; Basu N.; Xia J. Using MetaboAnalyst 5.0 for LC-HRMS spectra processing, multi-omics integration and covariate adjustment of global metabolomics data. Nat. Protoc. 2022, 17 (8), 1735–1761. 10.1038/s41596-022-00710-w. 35715522

[ref8] Tyanova S.; Temu T.; Sinitcyn P.; Carlson A.; Hein M. Y.; Geiger T.; Mann M.; Cox J. The Perseus computational platform for comprehensive analysis of (prote)omics data. Nat. Methods 2016, 13 (9), 731–740. 10.1038/nmeth.3901. 27348712

[ref9] Kanehisa M.; Goto S. KEGG: kyoto encyclopedia of genes and genomes. Nucleic Acids Res. 2000, 28 (1), 27–30. 10.1093/nar/28.1.27. 10592173 PMC102409

[ref10] Milacic M.; Beavers D.; Conley P.; Gong C.; Gillespie M.; Griss J.; Haw R.; Jassal B.; Matthews L.; May B.; et al. The Reactome Pathway Knowledgebase 2024. Nucleic Acids Res. 2024, 52 (D1), D672–D678. 10.1093/nar/gkad1025. 37941124 PMC10767911

[ref11] Shu L.; Zhao Y.; Kurt Z.; Byars S. G.; Tukiainen T.; Kettunen J.; Orozco L. D.; Pellegrini M.; Lusis A. J.; Ripatti S.; et al. Mergeomics: multidimensional data integration to identify pathogenic perturbations to biological systems. BMC Genom. 2016, 17 (1), 874 10.1186/s12864-016-3198-9. PMC5097440 27814671

[ref12] Cabukusta B.; Pauwels S. B.; Akkermans J. J. L. L.; Blomberg N.; Mulder A. A.; Koning R. I.; Giera M.; Neefjes J. The ORP9-ORP11 Dimer Promotes Sphingomyelin Synthesis; Cold Spring Harbor Laboratory, 2023. 10.7554/eLife.91345 PMC11302984 39106189

[ref13] Reinhold W. C.; Sunshine M.; Liu H.; Varma S.; Kohn K. W.; Morris J.; Doroshow J.; Pommier Y. CellMiner: a web-based suite of genomic and pharmacologic tools to explore transcript and drug patterns in the NCI-60 cell line set. Cancer Res. 2012, 72 (14), 3499–3511. 10.1158/0008-5472.CAN-12-1370. 22802077 PMC3399763

[ref14] Chang W.; Cheng J.; Allaire J.; Sievert C.; Schloerke B.; Xie Y.; Allen J.; McPherson J.; Dipert A.; Borges B. shiny: Web Application Framework for R. 2024. 10.32614/CRAN.package.shiny

[ref15] Argelaguet R.; Velten B.; Arnol D.; Dietrich S.; Zenz T.; Marioni J. C.; Buettner F.; Huber W.; Stegle O. Multi-Omics Factor Analysis-a framework for unsupervised integration of multi-omics data sets. Mol. Syst. Biol. 2018, 14 (6), e8124 10.15252/msb.20178124. 29925568 PMC6010767

[ref16] Wang B.; Mezlini A. M.; Demir F.; Fiume M.; Tu Z.; Brudno M.; Haibe-Kains B.; Goldenberg A. Similarity network fusion for aggregating data types on a genomic scale. Nat. Methods 2014, 11 (3), 333–337. 10.1038/nmeth.2810. 24464287

[ref17] Sievert C. Interactive Web-Based Data Visualization with R, Plotly, and Shiny; CRC Press, Taylor and Francis Group, 2020.

[ref18] Galili T.; O’Callaghan A.; Sidi J.; Sievert C. heatmaply: an R package for creating interactive cluster heatmaps for online publishing. Bioinformatics 2018, 34 (9), 1600–1602. 10.1093/bioinformatics/btx657. 29069305 PMC5925766

[ref19] Stacklies W.; Redestig H.; Scholz M.; Walther D.; Selbig J. pcaMethods--a bioconductor package providing PCA methods for incomplete data. Bioinformatics 2007, 23 (9), 1164–1167. 10.1093/bioinformatics/btm069. 17344241

[ref20] Friedman J.; Hastie T.; Tibshirani R. Regularization Paths for Generalized Linear Models via Coordinate Descent. J. Stat. Software 2010, 33 (1), 1–22. 10.18637/jss.v033.i01. PMC2929880 20808728

[ref21] Wu T.; Hu E.; Xu S.; Chen M.; Guo P.; Dai Z.; Feng T.; Zhou L.; Tang W.; Zhan L.; et al. clusterProfiler 4.0: A universal enrichment tool for interpreting omics data. Innovation 2021, 2 (3), 100141 10.1016/j.xinn.2021.100141. 34557778 PMC8454663

[ref22] Carlson M. org.Hs.eg.db: Genome wide annotation for Human. 2024.

[ref23] Workman P. The NCI-60 Human Tumor Cell Line Screen: A Catalyst for Progressive Evolution of Models for Discovery and Development of Cancer Drugs. Cancer Res. 2023, 83 (19), 3170–3173. 10.1158/0008-5472.CAN-23-2612. 37779429

[ref24] Johnson W. E.; Li C.; Rabinovic A. Adjusting batch effects in microarray expression data using empirical Bayes methods. Biostatistics 2007, 8 (1), 118–127. 10.1093/biostatistics/kxj037. 16632515

[ref25] Subramanian A.; Tamayo P.; Mootha V. K.; Mukherjee S.; Ebert B. L.; Gillette M. A.; Paulovich A.; Pomeroy S. L.; Golub T. R.; Lander E. S.; et al. Gene set enrichment analysis: a knowledge-based approach for interpreting genome-wide expression profiles. Proc. Natl. Acad. Sci. U.S.A. 2005, 102 (43), 15545–15550. 10.1073/pnas.0506580102. 16199517 PMC1239896

[ref26] Tavazoie S.; Hughes J. D.; Campbell M. J.; Cho R. J.; Church G. M. Systematic determination of genetic network architecture. Nat. Genet. 1999, 22 (3), 281–285. 10.1038/10343. 10391217

[ref27] aWong L. H.; Gatta A. T.; Levine T. P. Lipid transfer proteins: the lipid commute via shuttles, bridges and tubes. Nat. Rev. Mol. Cell Biol. 2019, 20 (2), 85–101. 10.1038/s41580-018-0071-5. 30337668

[ref28] Bateman A.; Martin M. J.; Orchard S.; Magrane M.; Ahmad S.; Alpi E.; Bowler-Barnett E. H.; Britto R.; Bye-A-Jee H.; Cukura A.; et al. UniProt: the Universal Protein Knowledgebase in 2023. Nucleic Acids Res. 2023, 51 (D1), D523–D531. 10.1093/nar/gkac1052. 36408920 PMC9825514

[ref29] Gehin C.; Lone M. A.; Lee W.; Capolupo L.; Ho S.; Adeyemi A. M.; Gerkes E. H.; Stegmann A. P.; Lopez-Martin E.; Bermejo-Sanchez E.; et al. CERT1 mutations perturb human development by disrupting sphingolipid homeostasis. J. Clin. Invest. 2023, 133 (10), e165019 10.1172/JCI165019. 36976648 PMC10178846

[ref30] Liebisch G.; Fahy E.; Aoki J.; Dennis E. A.; Durand T.; Ejsing C. S.; Fedorova M.; Feussner I.; Griffiths W. J.; Kofeler H.; et al. Update on LIPID MAPS classification, nomenclature, and shorthand notation for MS-derived lipid structures. J. Lipid Res. 2020, 61 (12), 1539–1555. 10.1194/jlr.S120001025. 33037133 PMC7707175

[ref31] Feringa F. M.; Hertog S. J. K.; Wang L.; Derks R. J. E.; Kruijff I.; Erlebach L.; Heijneman J.; Miramontes R.; Pompner N.; Blomberg N.; et al. The Neurolipid Atlas: a lipidomics resource for neurodegenerative diseases uncovers cholesterol as a regulator of astrocyte reactivity impaired by ApoE4. bioRxiv 2024, 10.1101/2024.07.01.601474.

[ref32] Chambers M. C.; Maclean B.; Burke R.; Amodei D.; Ruderman D. L.; Neumann S.; Gatto L.; Fischer B.; Pratt B.; Egertson J.; et al. A cross-platform toolkit for mass spectrometry and proteomics. Nat. Biotechnol. 2012, 30 (10), 918–920. 10.1038/nbt.2377. 23051804 PMC3471674

[ref33] Liu Z.; Jiang B.; Heer J. imMens: Real-time Visual Querying of Big Data. Comput. Graph. Forum 2013, 32 (3pt4), 421–430. 10.1111/cgf.12129.

[ref34] Sievert C. Interactive Web-Based Data Visualization with R, Plotly, and Shiny; CRC Press, Taylor and Francis Group, 2020.

