# Supplementary material for: iSODA: A Comprehensive Tool for Integrative Omics Data Analysis in Single- and Multi-Omics Experiments
Source: Anal Chem. 2025 Jan 31;97(5):2689–97. doi: 10.1021/acs.analchem.4c04355 (PMC11822744; doi:10.1021/acs.analchem.4c04355)
Supplement: Supplementary file 1 — ac4c04355_si_001.pdf [file ac4c04355_si_001.pdf]

Supporting Information

iSODA: A Comprehensive Tool for Integrative Omics Data Analysis in Single- and Multi-Omics Experiments

Damien Olivier-Jimenez[1], Rico J. E. Derks[1], Oscar Harari[2], Carlos Cruchaga[3], Muhammad Ali[3], Alessandro Ori[4], Domenico Di Fraia[4], Birol Cabukusta[5], Andy Henrie[6], Martin Giera[1], Yassene Mohammed[1,7]*

[1]Center for Proteomics and Metabolomics, Leiden University Medical Center, 2333ZA Leiden, Netherlands

[2]Department of Neurology, The Ohio State University, Columbus, OH 43210, United States of America

[3]Washington University School of Medicine in St. Louis, St. Louis, MO 63110, United States of America

[4]Leibniz Institute on Aging - Fritz Lipmann Institute (FLI), 07745 Jena, Germany

[5]Department of Cell and Chemical Biology, ONCODE Institute, Leiden University Medical Center, 2333ZA Leiden, Netherlands

[6]Datatecnica, Washington, DC 20037, United States of America

[7]Gerald Bronfman Department of Oncology, McGill University, Montreal, QC H3A 0G4, Canada.

***Corresponding author:** Yassene Mohammed | Center for Proteomics and Metabolomics, Leiden University Medical Center, 2333ZA Leiden, Netherlands | Email: y.mohammed@lumc.nl

Table of Contents

1.	iSODA user interface and available functionalities and interactive visualization	S3-S10
2.	Figure S1. Screenshot of iSODA showing four interactive analyses	S11
3.	Figure S2. A screenshot of the data summary of the LTP knockout library dataset as shown in iSODA	S12
4.	Figure S3. Lipidomics analysis of the LTP KO dataset. Comparison of TGs in StarD7 and StarD8 to control showing different trends regarding double bonds	S13
5.	Figure S3. Single-omics data analysis of the NCI-60 DNA-methylation, transcriptomics, and proteomics characterization data	S14-S15
6.	Figure S4. Heatmaps with feature selection to discriminate epithelial nature for the three omics used	S16
7.	Figure S5. Over representation analysis comparing epithelial and non-epithelial cell lines	S17
8.	Figure S6. Similarity Network Fusion on NCI-60 cell lines	S18
9.	Figure S7. MOFA on the NCI-60 multi-omics datasets used highlighting the top contributing features to factor 6	S19
10.	Figure S8. MOFA heatmaps for factors1, 3 and 6 across all datasets, displaying the top 50 contributing features	S20
11.	Table S1. Tools researched for omics data analysis	S21-S22
12.	Table S2. The gene targeted in the knockout experiment using CRISPR/Cas9 to produce the Lipid Trasport Protein library characterized by lipidomics	S23-S45
13.	Table S3. The human tumor cell lines used by National Cancer Institute as a screen to characterize novel compounds for tumor treatment	S46-S49
14.	Table S4. LTP family and gene knockout along with the cluster assigned to the genes by k-nearest neighbors	S50-S54
15.	Table S5. Performance testing of iSODA	S55
16.	Example of combining modular functionalities for novel analysis	S56-S57

iSODA – integrated Simple Omics Data Analysis

iSODA is a software developed to simplify both single- and multi-omics data analysis.

The user starts by creating one or multiple single-omics experiments (lipidomics, metabolomics, proteomics, transcriptomics, genomics) that can be accessed individually through the side bar. Each has their own dedicated UI with three main tabs for data upload, visualization, and functional analysis respectively. After having uploaded their data, interpreted it through the visualization and looked for the underlying biological processes at play via the functional analysis, several of these single-omics modules can be imported in the multi-omics modules.

Likewise, the multi-omics modules present an upload tab to select the single-omics experiments previously created, as well as a visualization tab to interpret the results. Some biological phenomena cannot be fully understood when glancing at single-omics data, but can unravel when considering the bigger picture. MOFA can achieve that by providing more information from the features' contribution, whereas SNF focuses more or the effects of integration on the samples.

Data tab

The Data tab within each single-omics module allows the user to upload their data via three subtabs named after the data they are expected to receive: Sample annotations, Measurement data, Feature annotations. While the app mainly uses the measurement data in plots and calculations, the sample and feature tables are used to annotation groups either on the samples or features, used for filtering purposes or mapped in the graphical representations of the data.

Sample annotations: tab designed for the upload and curation of sample metadata; the input table formatted with samples as rows and annotations as columns. This sample annotations table carries four essential columns: an ID column with unique identifiers, a type column specifying the type of each row (sample, blank, pool, or QC), a group column identifying the main sample groups to examine, and a batch column.

Measurement data: tab designed for the upload and curation of quantitative measurement data. The data must be provided with samples as rows, features as columns, and the values can only be numerical or missing. Upon upload, the table undergoes several normalization processes to produce other tables, for instance, total normalization and z-score scaling. Using the sample annotations, this tab can also filter the data to keep the most relevant features, displaying signals significantly above blanks. The lipidomics module expects features to be specifically formatted using lipid shorthand notations to extract structural information that is directly exported as feature annotations.

Feature annotations: tab used to upload feature metadata. This table is formatted with features as rows and annotations as columns. Feature annotations can be simple – one value per cell – or complex: multiple values per cell, separated by a pipe ("|") character. Complex annotations typically include ontologies, like classifications, pathways or gene ontologies and can be used in certain plots like the volcano plot, or for functional analysis.

Interactive visualization tab.

Interpreting results from experiments relies on processing the measured data in a specific way, depending on what needs to be achieved, and using sample and feature annotations to further enhance interpretability. The output then needs to be presented graphically in a meaningful manner

for the user to assess the results and explore their data. The visualization tab is designed to automate all these steps in a few clicks: the user can select up to four plots to be displayed simultaneously and they will immediately appear on screen using the most relevant input parameters. In addition to the selection, zooming and hovering capabilities provided by plotly, iSODA's plots can be interacted with using the four sets of parameters accessed via the sidebars:

- Input settings let the user select which transformed version of the measured data to use (raw, total normalized, z-scored) as well as other relevant input to be mapped on the plot, like sample or feature annotations.
- Data settings are used to access the parameters used in statistics and other background processes.
- Aesthetics settings control the colors, marker size, opacity and other display options used to enhance the readability of the data.
- Output settings allow a selection of the output formats for images (JPG, PNG, WEBP and SVG) as well as export options within or outside the app. Some of the plot results can be exported to the annotation tables to be used elsewhere on the app, like for instance sample or feature clusters. Most plots also have associated tables which can be downloaded from here, to be used outside the app.

Networks also have their own settings to adjust the physics parameters and display clear and uncluttered networks.

Current plot selection includes a dendrogram, volcano plot, sample correlation, feature correlation, heatmap and PCA. Moreover, the lipidomics module has three specific plots: class comparison, class distribution and double bonds plots

Dendrogram: Provides a rapid assessment of the similarity between samples and sample groups. The samples are clustered using hierarchical clustering and displayed in the form of a dendrogram, which can be mapped with sample annotations to find out if the unsupervised groups can be explained by known factors. The number of desired clusters can be set and saved on the sample annotations table to be used elsewhere on the app. For example, in Figure 4 one can see how this functionality is used to highlight that cancer cell lines are primarily clustered according to whether the cells are epithelial or not.

Volcano Plot: A standard way of identifying features that distinguish two sample groups. The features are displayed in a scatter plot, the y-axis representing the -Log10(p-value) and the x-axis the Log2(fold change). The p-values are calculated using either a t-Test or a Wilcoxon test and can be adjusted with multiple methods. The fold changes are calculated using either the mean or the median values. In some cases, p-values or fold changes cannot be calculated because of one group having too few or no values for a feature. These features are reported in violin plots surrounding the main scatter plot. The left-to-right spread of the features reflects their differential production or expression between the two sample groups (higher absolute fold change), and the more a feature is located on the top of the plot, the more significant it is (lower p-value). The adjacent violin plots display the features that can be regarded as some of the most important, since they are absent in one of both groups. To highlight relevant features, the user can set p-value and fold change thresholds, manifesting in dashed lines across the volcano plot. Features above these thresholds can be exported to the feature annotations table and used, for instance, in functional analysis. As an alternative to viewing the differential expression, the user can also map feature annotations on the volcano plot to assess if a known group of features is being over- or underexpressed.

Sample Correlation Plot: An alternative representation to the dendrogram visualization, augmented with a heatmap to display and better understand sample clusters. The heatmap displays the correlation coefficient (Pearson or Spearman) between each sample pair, and the sample order is arranged using hierarchical clustering, the results of which are also displayed with the dendrograms on the sides of the heatmap. As with the dendrogram, sample annotations can be mapped on the leaves of the dendrograms. While the dendrogram only displayed the clusters, the sample correlation heatmap also shows how close (or different) samples are based on the correlation coefficients. The sample clusters are also emphasized with the heatmap colors. The calculated clusters can be exported to the sample annotations table.

Feature Correlation Plot: Shows which features are correlated and highlights feature groups thanks to the hierarchical clustering and a heatmap. It is the same representation as the sample correlation but applied to features. Due to the limited rendering capabilities on browsers, the plotted data can be reduced using a maximum feature count filter and a minimum correlation coefficient threshold, thus displaying only the best scoring correlations. Like the sample correlation, the correlation coefficients are shown on the heatmap, and the feature ordering is based on hierarchical clustering, helping highlight groups of features and their correlations, notably with the color coding. Obtaining feature annotations is not as straightforward as gathering sample information: creating groups of features based on this feature correlation is a good starting point when nothing else is available. The generated feature clusters can then be exported to the feature annotations table.

Heatmap: A visual representation of the measured data combining sample and feature clustering, allowing to find out if some groups of samples are associated with groups of features. This is achieved by displaying the z-score scaled data as a heatmap with samples as columns and features as rows. Based on the data, hierarchical clustering is applied to samples and features, highlighting areas in the dataset where groups of samples and feature expression coincide. Since this plot combines sample and features, their respective annotations can be mapped on the heatmap. Moreover, Lasso and Elastic-Net Regularized Generalized Linear Models can be applied on the features to keep only those best segregating the sample groups. Using the representation, the user can directly assess if the sample group mapping can be associated with known feature groups, driving the observed clustering.

Principal Component Analysis: Representation of the samples and the features in a reduced dimensional space that retains as much variance as possible from the dataset, highlighting potential sample groups and the features driving the groupings. In iSODA, PCA is delineated in three plots: explained variance, scores plot and loadings plot, with the option to display the scores and loadings plots together. The user can choose the number of computed principal components and display the associated explained variance. Two PCs can then be chosen for the 2D scores and loadings plots. The scores plot displays the coordinates of the original samples in this new 2D space. Likewise, the loadings plot represents each feature's contribution to the two selected PCs. Sample and feature annotations can be mapped on the markers. The scores plot can be used to identify trends and sample groupings, while the loadings plot can identify the features contributing to these trends. Like with the heatmap, Lasso and Elastic-Net Regularized Generalized Linear Models can be used to specifically select the most segregating features.

Class Distribution: A lipidomics specific visualization that provides a summary of the mean lipid class concentrations for all sample groups. The lipid concentration is displayed on the y-axis and the lipid classes on the x-axis. The class-wise group concentrations will be represented as colored bar

plots. This plot allows the user to directly spot concentration differences between sample groups and assess the relative lipid class concentrations with the shared y-axis.

Class Comparison: Grid version of the class distribution, allowing a better assessment of the minute group concentration variations. Sample group concentrations are represented by bar plots for each lipid class. Plots are arranged in a grid, each one with its own y-axis. Like for the class distribution, the bars represent the mean concentrations. On top of the bars, box plots represent the median concentrations and the quartiles, along with the individual sample concentrations as markers. With the separate axes, group concentrations can be better examined. The additional box plots and markers help identify the sample distribution for each group and potential outliers.

Double Bonds Plot: Another lipidomics specific plot that works in conjunction with the volcano plot, allowing a more structure specific examination of the lipid class differences between two sample groups. Two sample groups and a lipid class are selected, and like with the volcano plot, p-values and fold changes are calculated. Each individual lipid species is displayed on a bubble plot, the y-axis representing the double bond count and the x-axis the carbon count. The carbon and double bond counts can be specified to the side-chain level or the total values. Each bubble – or lipid species – is displayed with a size relative to the p-value and colored according to fold change. The bubble size emphasizes the most relevant features while the coloring highlights the direction of the expression. Combined with the structural information provided by the double bonds and carbon counts, this plot can reveal structural trends that might be biologically relevant to differentiate the two conditions.

Functional analysis tab

This tab is designed for functional analysis, a deeper dive to reveal the factors differentiating two sample groups. It is subdivided into three subtabs, "Functional comparison" to set up the analyses, and two visualization tabs to plot the results: "Enrichment" and "Over-representation". The functional comparison tab is divided into multiple sections to prepare the input data and the parameters for the analyses. The sample selection lets the user choose the appropriate data table and the two sample groups to compare. The feature selection section lets the user choose which features will be kept for the subsequent functional analysis. This can be done either with a statistical in conjunction with p-value and fold change thresholds; or using a custom user selection via the feature annotation table, which includes groups exported from other plots, like the volcano plot and the feature correlation. The last two sections are dedicated to the parameters for enrichment (EA) and overrepresentation (ORA) analyses, notably selecting the "Feature sets" to be used. Feature sets are groups of features sharing a role, like the Gene Ontology "Biological Processes", "Molecular Functions" and "Cellular Components", which are automatically available for non-small molecule omics. Alternatively, the feature sets can be supplied in the feature annotation table. Since each feature can be associated with multiple feature sets, these can be separated by a pipe ("|") character.

Enrichment analysis. Computational method designed to determine whether feature sets show statistically significant, concordant differences between two sample groups. It is based on the original Geneset Enrichment Analysis (GSEA) but applied to any omics using appropriate feature sets. The algorithm starts by ranking all features in the dataset based on their differential expression between the two sample groups, creating a ranked list. An Enrichment Score (ES) is then calculated for each predefined feature set, which reflects the degree to which features from that set are represented at the extremes of the ranked feature list. This is achieved by walking down the ranked

list and increasing a running-sum statistic when a feature in the feature set is encountered and decreasing it when it is not. The ES is the maximum deviation from zero encountered in walking the list, corresponding to a weighted Kolmogorov-Smirnov-like statistic. A positive ES indicates a feature set enrichment at the top of the ranked list while a negative ES indicates a feature set enrichment at the bottom of the ranked list. Subsequently, a permutation test is applied to estimate the significance of the ES and derive a p-value, indicating the likelihood that the observed enrichment is due to chance. The permutations are then used to calculate the Normalized Enrichment Score (NES) to account for differing set sizes. By applying a feature set p-value threshold, the enrichment analysis can identify sets that are significantly associated with one sample group or the other. Within these sets, some features contribute more than others to the enrichment score: the core enrichment – or leading-edge subset – are features that appear in the ranked list at or before the point where the running sum reaches its maximum deviation from zero.

Over-representation analysis (hypergeometric test / fisher exact test). Over-representation Analysis (ORA) is a statistical method used to identify if a feature set is represented more frequently in a list of pre-selected features of interest than would be expected by random chance. These pre-selected features of interest are set apart from the other features either by using a statistical test along with p-value and fold change thresholds; or selected manually by the user via the feature annotations table. A hypergeometric test (or Fisher's exact test) is then applied to assess the probability (p-value) that the observed frequency of a specific feature set within the list occurs more than would be expected by chance, given the distribution of annotations in the universe of features (i.e. the complete list of features). Sets with a low p-value are significantly over-represented in the list of features of interest, implying they are relevant in the studied sample groups.

Functional analysis plots. Enrichment and over-representation analysis use similar plots that rely on the same metrics, highlighting the top feature sets and features that differentiate the compared sample groups.

Dot Plot: This plot displays the most significant feature sets resulting from the analysis. They are represented on the y-axis of the bubble plot, against their associated feature ratio on the x-axis. Each bubble – or set – is colored based on their p-value and sized proportional to their feature count. For enrichment analysis, the plot differentiates between suppressed and activated sets using enrichment scores, highlighting the sets associated with one sample group or the other. This visualization aids in identifying biologically significant feature sets that contribute to the observed differences between the two groups under comparison.

Bar Plot: This plot illustrates the top feature sets identified from an overrepresentation analysis, similar to the previously described dot plot. The y-axis lists the feature sets, while the x-axis shows the feature ratios. Each bar is colored according to the p-value of the set it represents, showcasing a clear visual comparison of the statistical significance across different feature sets.

Ridge Plot: This ridge plot provides a multi-layered visualization of the top feature sets identified in an enrichment analysis, with each set plotted along the y-axis against the Log2(fold change) of its associated features on the x-axis. The distribution of features within a set is represented by a density curve along the x-axis, creating a series of vertically stacked ridges. Each ridge is color-coded according to the p-value of its corresponding feature set. Horizontally, the plot illustrates the spread of features from one extreme to the other, indicating their predominance in one of the sample groups. Vertically, the plot allows for comparison of these feature distributions across different sets.

CNET Plot: This network visualization represents feature sets and their associated features as nodes, connected by edges that link features to their respective feature sets. Annotations for both features and sets can be mapped onto the nodes to provide additional information. The CNET plot provides a way to visualize and interpret the complex relationships between features and their feature sets. By displaying Log2(fold change) values on the feature nodes, the plot facilitates the observation of potential differential expressions among feature sets. The plot not only shows which feature sets are influenced by the current analysis but also highlights the associated features. Since these features may belong to multiple sets, the plot often reveals clusters of feature sets, indicating broader, more complex phenomena that aggregate the effects of individual sets.

eMap Plot: The enrichment map (eMap) plot is a streamlined alternative to the CNET plot, specifically designed to handle large numbers of feature sets without becoming cluttered. Unlike the CNET plot, the eMap plot omits feature nodes, reducing complexity and avoiding the creation of unreadable "hairball" networks. Instead, it creates set-to-set edges that reflect the number of shared features relative to the total associated features, quantified by Jaccard's similarity score (default) to indicate connection strength between two feature sets. The network can be further simplified by applying a similarity score threshold to produce more manageable feature set clusters. Node sizes are scaled based on the feature count of each set, while node coloring reflects p-values, and edge thickness is proportional to the similarity score. This layout allows users to discern larger biological mechanisms coalescing smaller feature sets, offering insights on a bigger scale than what is provided by the CNET plot.

Multi-omics integration

The main aim of omics integration is finding and possibly quantifying patterns in the multiple omics measurements that are not obvious when considering them separately. Each omics layer (genomics, proteomics, transcriptomics, metabolomics) provides a different perspective on the biological system, their integration offers a holistic view of the biological processes and mechanisms at play.

MOFA (Multi-Omics Factor Analysis). MOFA is an unsupervised integration method designed to reduce the complexity of large-scale omics datasets into a manageable number of latent factors (Z). These factors capture the underlying sources of variation across the datasets, providing insights into biological processes that might be driving the observed patterns. Factors can be assimilated to principal components in PCA, each explaining a portion of the variance from the dataset. Similarly, samples and features have weights (W) associated to each factor.

Explained Variance Plot: This plot provides an overview of the contribution of each omics dataset to the computed factors by displaying the variance explained for each factor in the form of a heatmap. Additionally, two bar plots on the sides show the cumulative variance for each omics and each factor. This arrangement allows researchers to identify factors unique to specific omics as well as those that are significant contributors across multiple omics, potentially indicating a shared biological phenomenon. The plot serves as a useful reference in conjunction with other plots to identify the most relevant factors for the omics under investigation.

Factor Plot: This plot summarizes the sample factor weights and assesses their potential to explain group differences. It displays samples on the x-axis and their corresponding factor weights on the y-axis. Multiple factors can be represented simultaneously, with samples differentiated according to sample annotations. Optional violin plots give a better impression of sample distributions. By

mapping sample annotations, researchers can determine whether certain factors distinctly separate specific sample groups. These factors may then be analyzed in greater detail using additional visualizations.

Combined Factors Plot: This visualization complements the factor plot by showing whether paired factors explain variations between sample groups. One or multiple factors can be selected, which generates a grid containing two types of plots. Plots on the diagonal illustrate the distribution of samples across the loadings of a single factor, while off-diagonal plots depict the distribution across two factors. Density plots are used to visualize group distributions and to assess whether factors explain differences between sample groups. Additionally, the combined factor scatter plots enable the examination of whether sample groups can be characterized by more than one factor, similar to how PCA plots sample scores for two components.

Feature Weights Plot: This plot illustrates the contributions of individual features to a selected factor within a specified omics dataset. Users select a factor and omics dataset, and the features are displayed with their factor weights on the x-axis and their contribution rank on the y-axis. Typically, features will align along a sigmoid curve, or alternatively, a logarithmic curve when absolute factor weights are considered. This format allows users to quickly identify the most influential features at either end of the sigmoid curve or at the peak of the logarithmic curve. Additionally, mapping feature annotations can provide deeper insights into which groups of features significantly influence the factor.

Feature Top Weights Plot: This plot serves as an alternative to the feature weights plot, focusing specifically on the highest-scoring features. Users select an omics-factor pair, and the plot displays the top contributing features in a lollipop plot. Contributions can be further filtered to show either the top negative or positive contributing features, enabling a more targeted analysis of feature impact.

MOFA Heatmap: This visualization integrates the single-omics heatmap approach with the identification of a factor's top contributing features. Similar to the heatmap available in the single omics module, the MOFA heatmap presents the measured data and provides clustering for both samples and features. Unlike the standard heatmap, which employs supervised discriminant analysis for feature selection, the MOFA heatmap selects the top-ranked features based on their factor contributions, regardless of whether these are negative or positive. This enables the user to view if certain sample groups can be distinguished by the most relevant features of a factor. If no distinct groups are apparent, it may indicate that the factor is associated with an overlooked biological process.

Scatter Plot: This plot enables users to assess the correlation between specific feature signals and the dataset’s sample weights. Users can select an omics dataset along with the top contributing features to a factor—whether negative, positive, or both. A grid of scatter plots is then generated, each representing a distinct feature. In each scatter plot, samples are plotted with their measured values for that feature on the y-axis and their sample weights on the x-axis. A Pearson correlation coefficient and a confidence interval are calculated and displayed for each plot to quantify the strength and certainty of the correlation. Sample annotations can be mapped to visually emphasize different sample groups. This scatter plot provides a detailed examination of how the measured top-weighted features correlate with sample weights, possibly explaining sample groups.

SNF (Similarity Network Fusion). SNF is another computational method for integrating different single-omics datasets. SNF works by constructing individual networks for each dataset, each representing the similarity between samples. These networks are then fused into a single network through an iterative process capturing both the shared and unique characteristics of each dataset. For a single-omics dataset, a distance matrix between the samples is created, using the distance method chosen by the user (e.g. Euclidean). The distance matrix is then transformed into an affinity matrix, capturing the local neighborhood relationships among the samples by employing the the K-nearest neighbors and sigma parameters. The former specifies the number of nearest neighbors for each sample based on the distance matrix, and the latter determines how rapidly the affinity between samples decreases when constructing the affinity matrix. The samples are then grouped into K clusters through spectral clustering. In the case of the fusion heatmap and the similarity fusion network, the affinity matrices of each individual omics are combined, using the additional K-nearest neighbors parameter and a designated number of iterations (T) for the diffusion process, thereby enhancing the comprehensive understanding of the dataset through a multifaceted approach.

Similarity Heatmap: This heatmap illustrates the impact of spectral clustering on omics datasets separately. The x-axis and y-axis display samples organized by cluster groups, while the cell values indicate affinity levels. Cluster assignments can be shown as side annotations alongside other sample characteristics, enabling evaluation of whether the clustering within single-omics data aligns with anticipated sample groupings.

Fusion Heatmap: This heatmap presents sample affinities by integrating multiple omics datasets. Users can select two or more single-omics datasets, which are each subjected to spectral clustering. The outcomes are then merged to form a comprehensive affinity matrix that encapsulates the characteristics of the integrated omics data. Like the similarity heatmap, this heatmap organizes samples according to their cluster affiliations and allows for the mapping of sample annotations. The fusion heatmap is particularly useful for identifying sample similarities that emerge when multiple data perspectives are considered simultaneously.

Similarity Network: This network graph visualizes the affinity matrix for single-omics datasets. The similarity matrix is converted into a network, where all samples are connected by edges that denote the affinity values between them. To enhance readability, the network is trimmed to retain only the highest-scoring edges, a percentage which can be set by the user. Nodes are color-coded based on cluster affiliation or other sample annotations, and edge thickness is proportional to the affinity value. By interactively hovering over nodes and edges, additional details will be displayed on screen. This network serves as an alternative to the similarity heatmap, enabling users to verify whether the clustering of single-omics data corresponds with predefined sample groups and to explore complex relationships like affinities across multiple sample groups.

Similarity Fusion Network: This network graph represents the multi-omics sample clustering, employing the same approach as the single-omics similarity network but based on the fusion heatmap. Users can select two or more single-omics datasets, and the combined affinity matrix is then transformed into a network. Here again, the user can select the top scoring edges to be displayed. The edge width corresponds to the affinity values, while the coloring denotes the number of omics datasets supporting each connection, based on the set edge threshold. This network is interpreted like the single-omics network, with the added benefit of sourcing the connections from multiple datasets. The similarity fusion network is particularly effective in revealing groupings less apparent in

single-omics analyses. It also identifies unique connections that emerge only when multiple omics datasets are considered together.

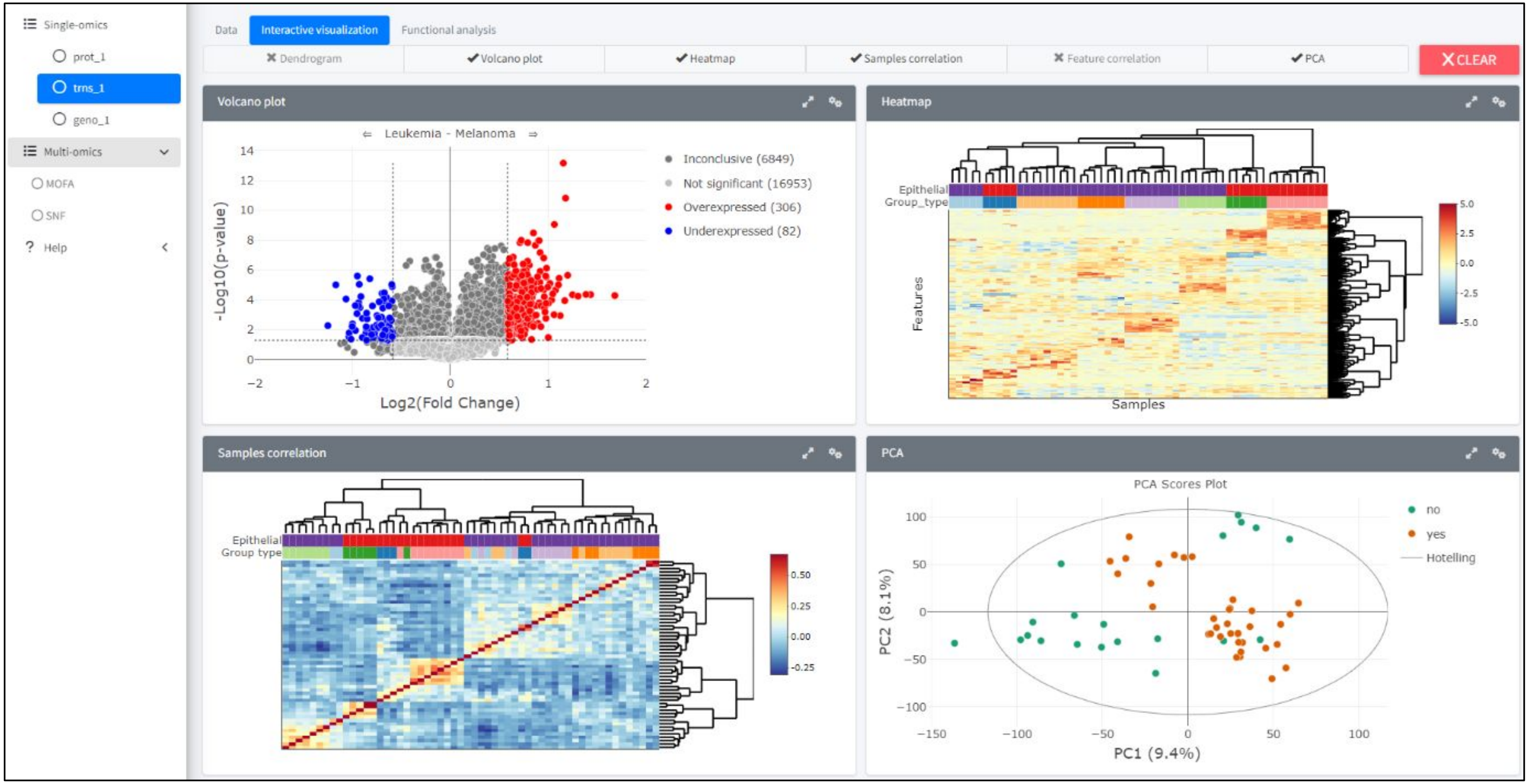

Figure S1. Screenshot of iSODA showing four interactive analyses.

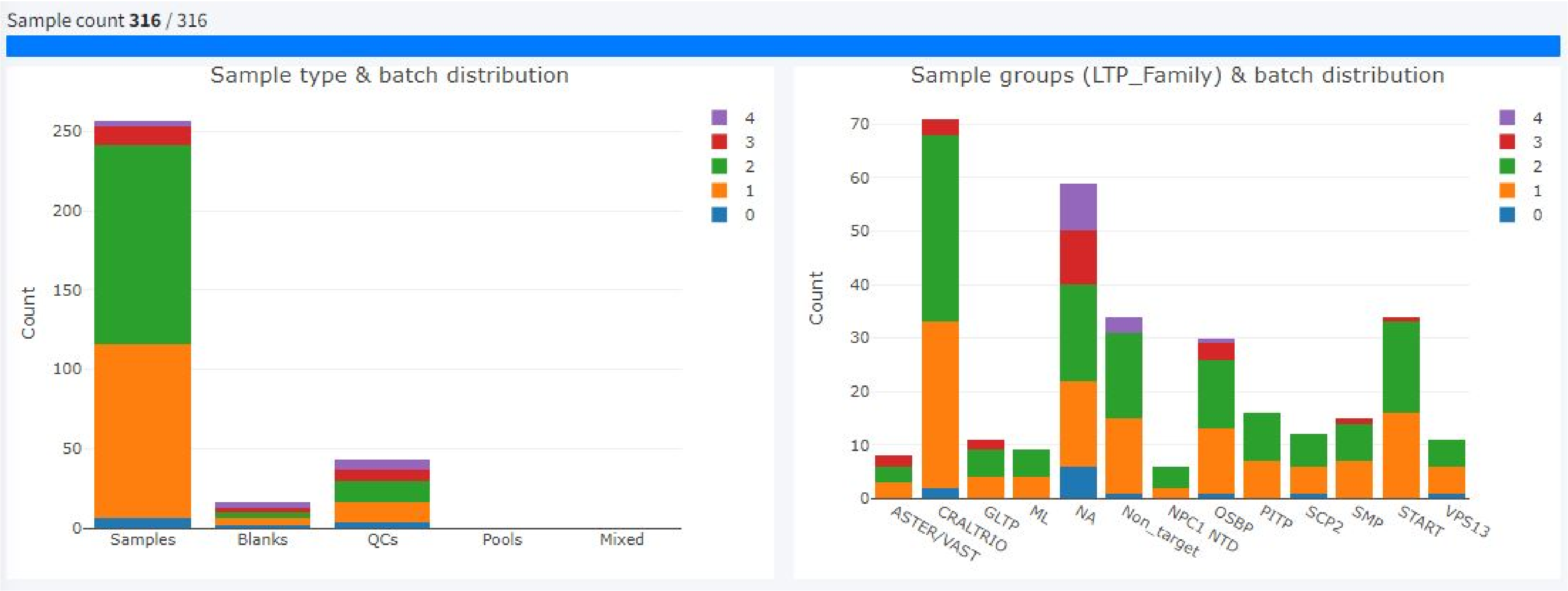

Figure S2. A screenshot of the data summary of the LTP knockout library dataset as shown in iSODA.

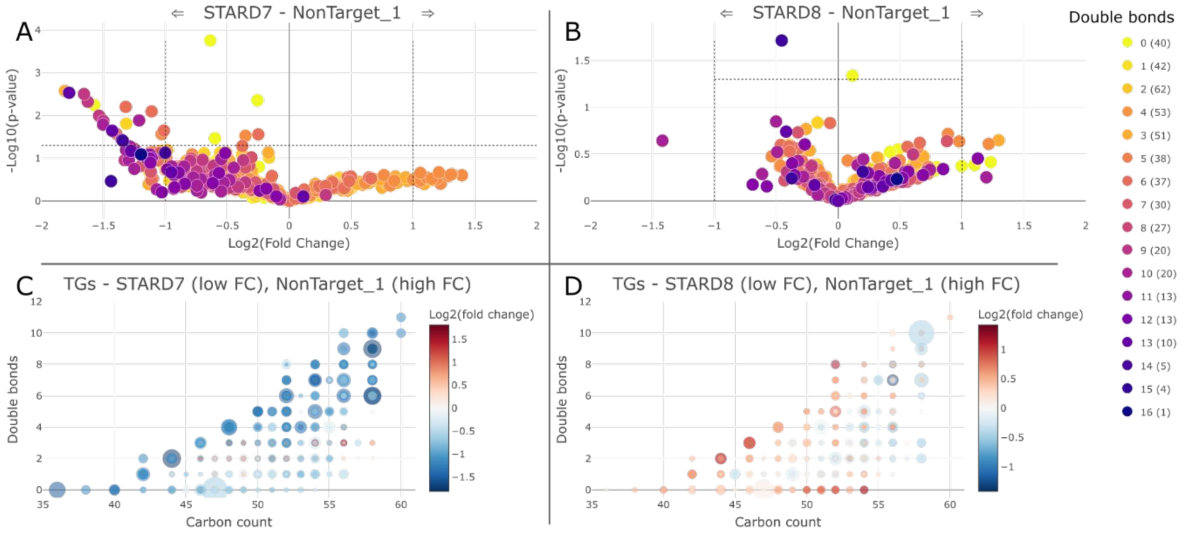

Figure S3. Lipidomics analysis of the LTP KO dataset. Comparison of TGs in StarD7 and StarD8 to control showing different trends regarding double bonds. Total normalized tables were used.

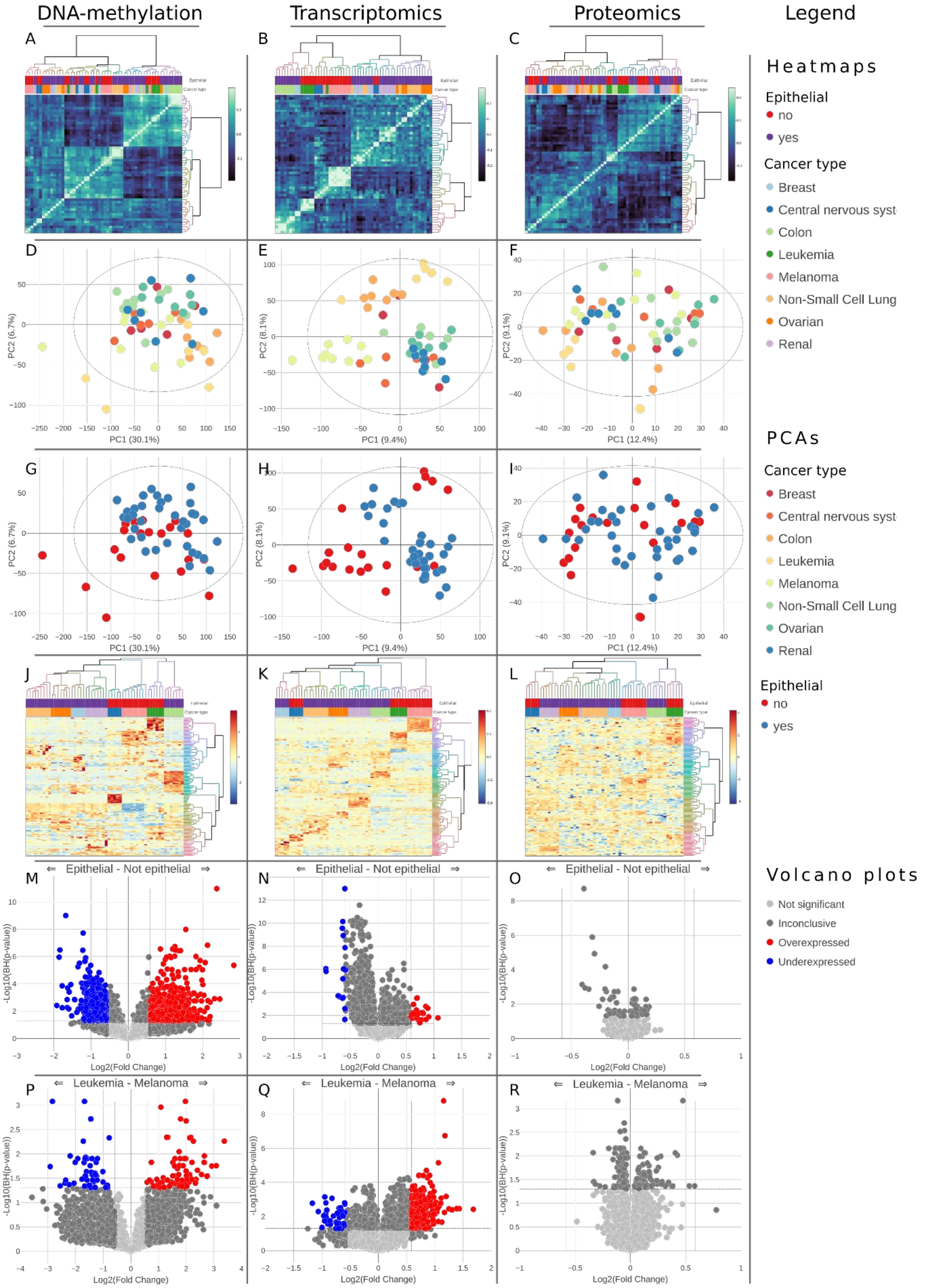

Figure S4. Single-omics data analysis of the NCI-60 DNA-methylation, transcriptomics, and proteomics characterization data. A-C correlation heatmaps showing similar clusters based on Pearson's correlation. D-I represent PCA score plots (z-scored total normalized data, the nipals PCA method)

with D-F are colored by cancer type and G-I by epithelial nature. J-L heatmaps with feature selection to discriminate cancer type for the three omics (z-scored total normalized data, LASSO alpha set to 0.8, Euclidean distance, ward.D2 clustering). Annotations mapped on top included cancer type and epithelial nature of the tissue. k=8 clusters was set and is rendered in the colored dendrogram. M-O volcano plots comparing cell lines according to their epithelial nature, and P-R comparing leukemia to melanoma samples (total normalized data, t-test, FC using mean, B-H p-value adjustment, p-value threshold was set to 0.05, FC threshold was set to 1.5).

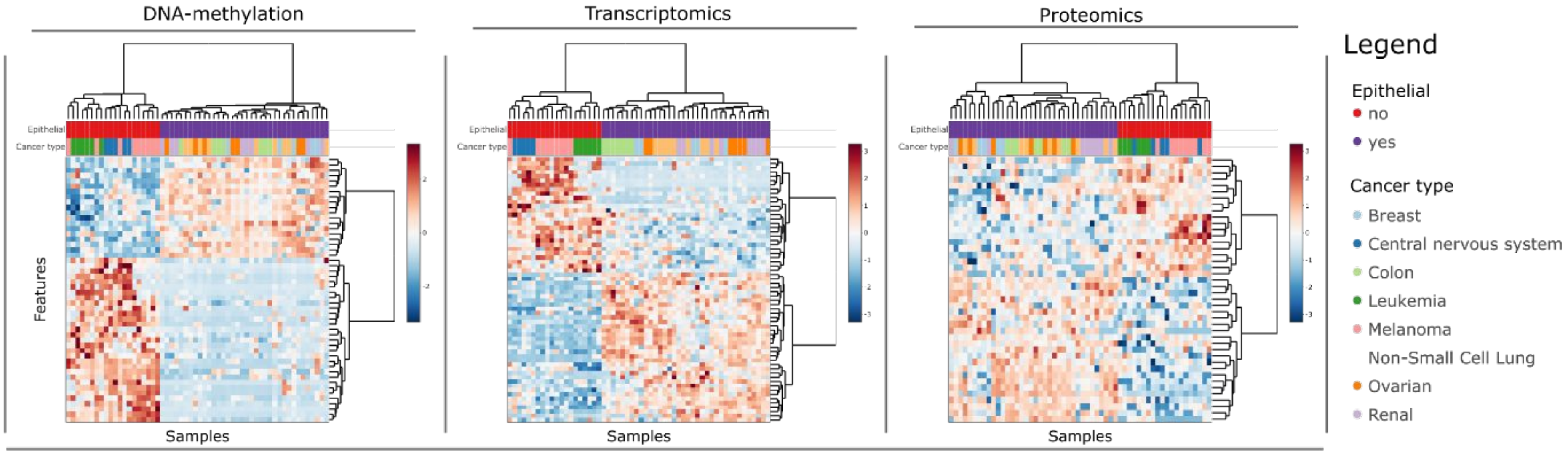

Figure S5. Heatmaps with feature selection to discriminate epithelial nature for the three omics (z-scored total normalized data, LASSO alpha set to 0.8, Euclidean distance, ward.D2 clustering). Annotations mapped on top included cancer type and epithelial nature of the tissue. k=8 clusters were set and rendered in the colored dendrogram.

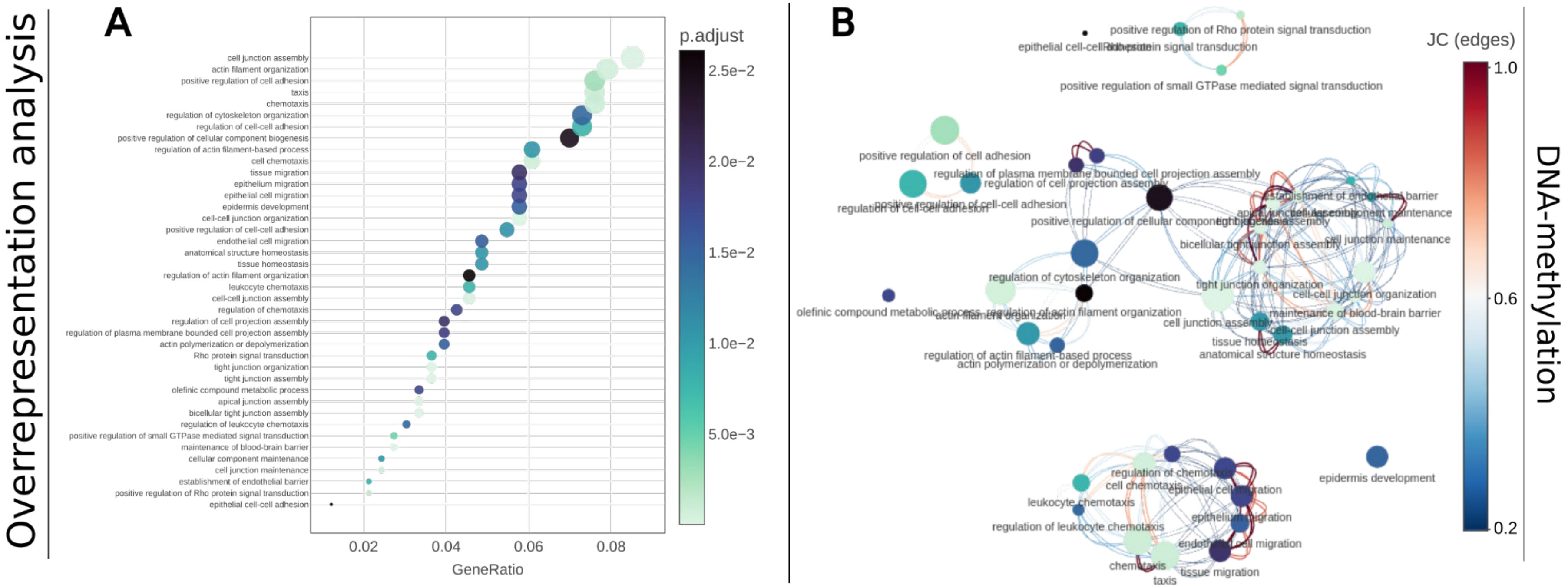

Figure S6. Over representation analysis comparing epithelial and non-epithelial cell lines with the DNA- methylation data, using the under- and overexpressed features. Over- and underexpressed DNA-methylated genes were determined by the volcano plot analysis and imported into functional analysis using the built-in save tool feature. A shows top 20 annotations. B displays top 20 set, node coloring reflects adjusted p-value, their size is scaled to gene counts, JC similarity score used with threshold set to 0.2, edges colored according to JC score.

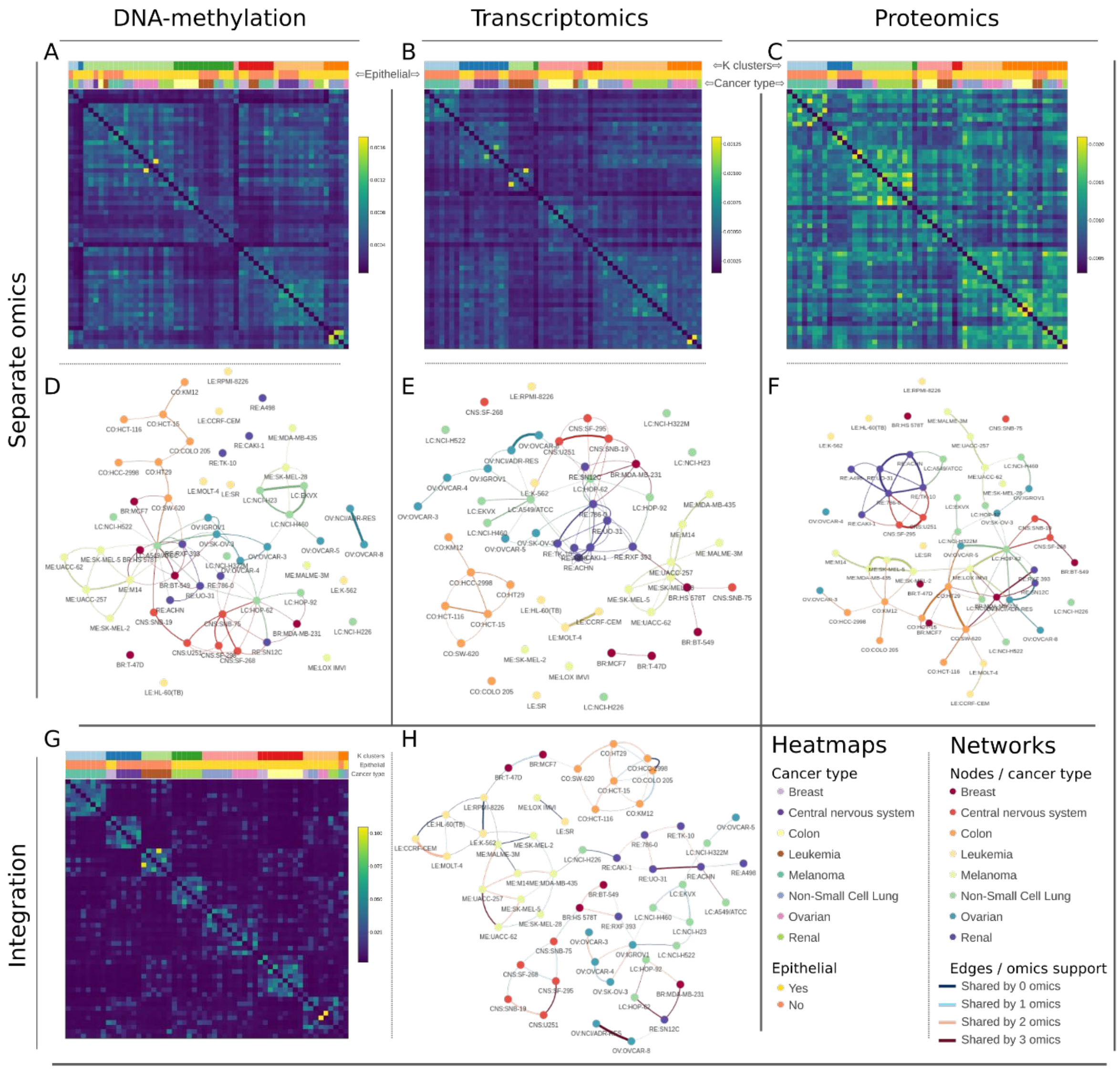

Figure S7. Similarity Network Fusion on NCI-60 cell lines. A-C are similarity heatmaps and D-F are the network of the three omics individually. G shows the fusion heatmap and network results. k-nearest neighbors method was set to 5, sigma to 0.5, using an Euclidean distance, and setting k clusters to 8. The heatmaps were mapped with k clusters, cancer type and epithelial status, while the networks were colored by cancer type and 5% of the top scoring edges were kept.

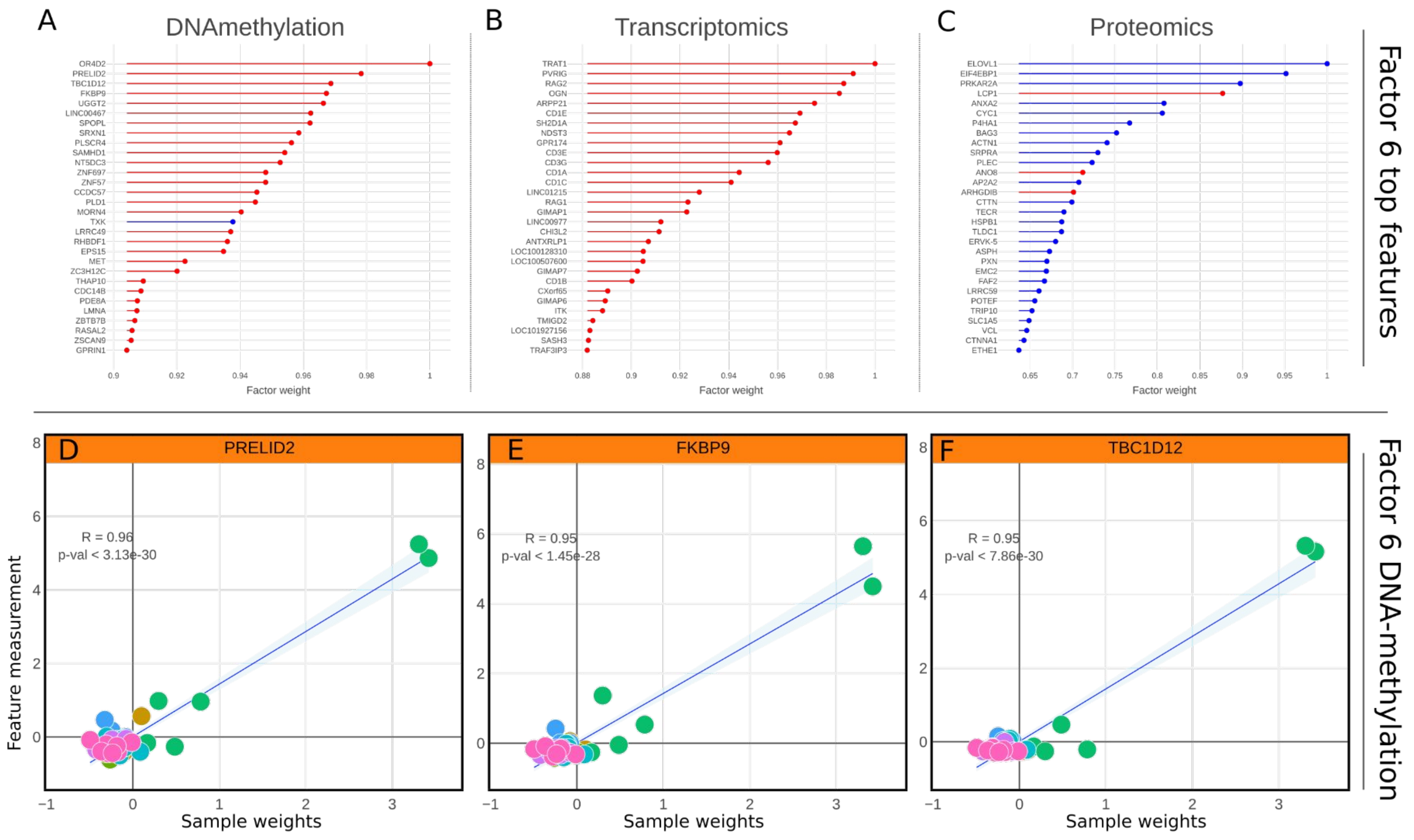

Figure S8. MOFA on DNA-methylation, transcriptomics, and proteomics datasets of the NCI-60 cell lines highlighting the top contributing features to factor 6. A-C show top discriminating factors according to MOFA in the three omics datasets used. D-F are scatter plots for these top contributing features, plotting the actual measured value for the feature (normalized) against the sample weights.

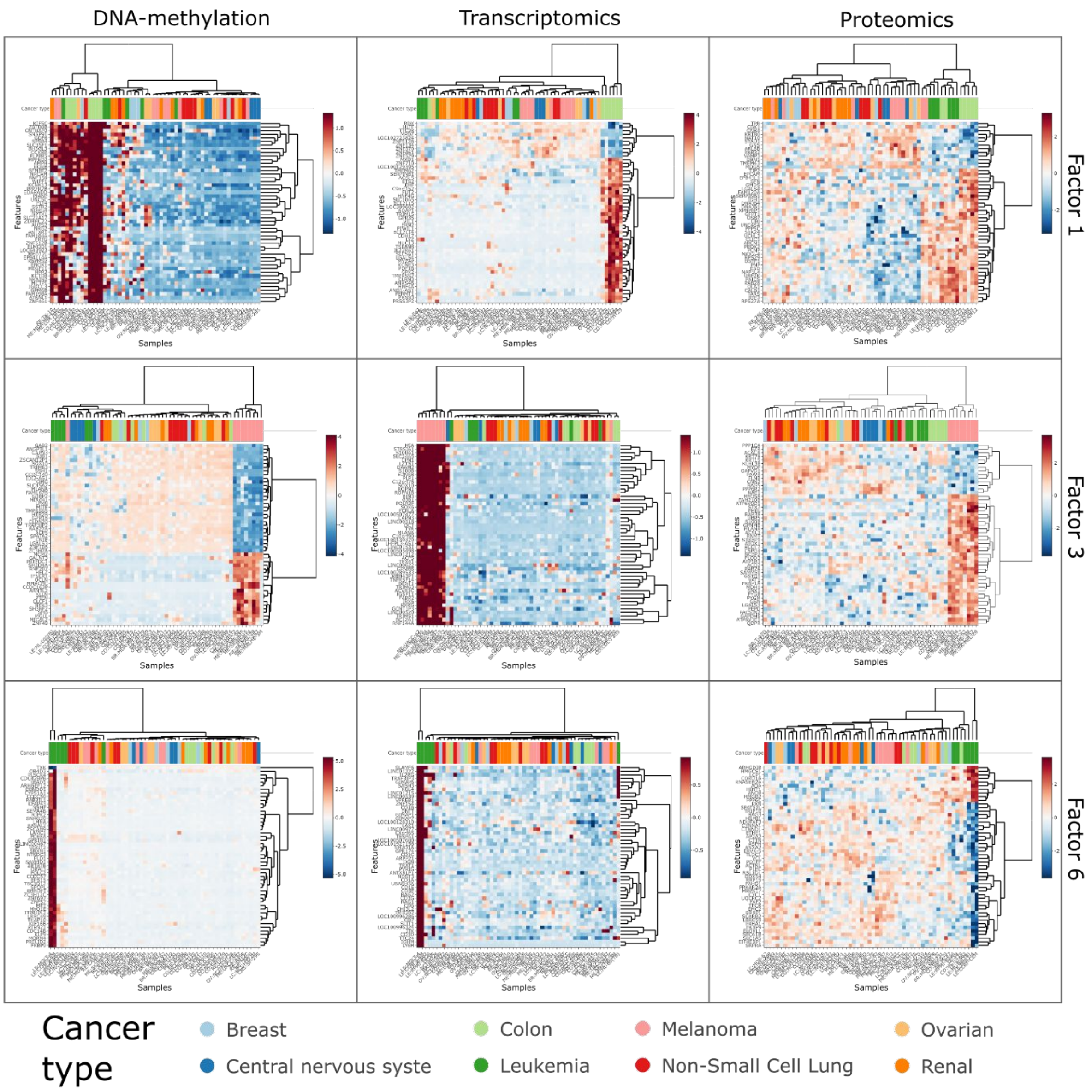

Figure S9. MOFA heatmaps for factors1, 3 and 6 across all datasets, displaying the top 50 contributing features (positive or negative contributions).

Table S1. Tools researched for omics data analysis.

Name	Year	Theme	URL
KaPPA-View	2005	Plants	https://doi.org/10.1104/pp.105.060525
g:Profiler	2007	Gene data	https://doi.org/10.1093/nar/gkm226
MetPA	2010	Metabolomics data	https://doi.org/10.1093/bioinformatics/btq418
Paintomics	2010	General	https://doi.org/10.1093/bioinformatics/btq594
IMPaLA	2011	General	https://doi.org/10.1093/bioinformatics/btr499
Metscape 2	2011	General	https://doi.org/10.1093/bioinformatics/btr661
VisANT 4.0	2013	Networks	https://doi.org/10.1093/nar/gkt401
3Omics	2013	General	https://doi.org/10.1186/1752-0509-7-64
CARMO	2015	Plants	https://doi.org/10.1111/tpj.12894
Mergeomics	2016	General	https://doi.org/10.1186/s12864-016-3057-8
OASIS	2016	Cancer	https://doi.org/10.1038/nmeth.3692
Pathview Web	2017	General	https://doi.org/10.1093/nar/gkx372
OmicsNet	2018	Networks	https://doi.org/10.1093/nar/gky510
PaintOmics 3	2018	General	https://doi.org/10.1093/nar/gky466
miRNet 2.0	2020	miRNA	https://doi.org/10.1093/nar/gkaa467
Argonaut	2020	General	https://doi.org/10.1016/j.patter.2020.100122
Imaging-AMARETTO	2020	Imaging	https://doi.org/10.1200/CCI.19.00125
CHOmics	2020	Cell lines	https://doi.org/10.1371/journal.pcbi.1008498
ADAS-viewer	2020	Alzheimer	https://doi.org/10.1038/s41540-021-00177-7
Panomicon	2020	Gene data	https://doi.org/10.1016/j.heliyon.2020.e04618
GraphOmics	2021	General	https://doi.org/10.1186/s12859-021-04500-1
Arena3D	2021	Networks	https://doi.org/10.1093/nar/gkab278
NeDRex	2021	Drug repurposing	https://doi.org/10.1038/s41467-021-27138-2
Mergeomics 2.0	2021	General	https://doi.org/10.1093/nar/gkab405
MiBiOmics	2021	General	https://doi.org/10.1186/s12859-020-03921-8
OmicsAnalyst	2021	General	https://doi.org/10.1093/nar/gkab394
TIMEOR	2021	Gene data	https://doi.org/10.1093/nar/gkab384
MAINE	2021	Gene data	https://doi.org/10.1093/bioinformatics/btab862
iNetModels 2.0	2021	Database	https://doi.org/10.1093/nar/gkab254
MOVIS	2022	General	https://doi.org/10.1016/j.csbj.2022.02.012
OmicsNet 2.0	2022	Networks	https://doi.org/10.1093/nar/gkac376
MicrobioSee	2022	Microbiology	https://doi.org/10.3389/fgene.2022.853612
HTT-OMNI	2022	Huntingtin	https://doi.org/10.1016/j.mcpro.2022.100275

ExpressVis	2022	Trns & Prot	https://doi.org/10.1093/nar/gkac399
Visual Omics	2022	Gene data	https://doi.org/10.1093/bioinformatics/btac777
BALDR	2023	Diabetes	https://doi.org/10.1371/journal.pcbi.1011403
PlantMetSuite	2023	Plants	https://doi.org/10.3390/plants12152880

Table S2. The gene targeted in the knockout experiment using CRISPR/Cas9 to produce the Lipid Trasport Protein library characterized by lipidomics. These 90 genes are from 11 families OSBP (n=30), START (n=34), PITP (n=16), GLTP (n=11), CRAL-TRIO (n=71), SMP (n=15), VPS13 (n=11), NPC1 NTD (n=6), ML (n=9), SCP2 (n=12) and ASTER/VAST (n=8).

Target Gene Name	Target Gene ID	Ensembl Gene ID	UniProtKB accession	LTP Family	Protein names	Gene Ontology (biological process)	Gene Ontology (cellular component)	Gene Ontology (molecular function)
OSBPL8	114882	ENSG00000091039	Q9BZF1	OSBP	Oxysterol-binding protein-related protein 8 (ORP-8) (OSBP-related protein 8)	fat cell differentiation [GO:0045444]; negative regulation of cell migration [GO:0030336]; negative regulation of sequestering of triglyceride [GO:0010891]; phosphatidylserine acyl-chain remodeling [GO:0036150]; phospholipid transport [GO:0015914]; positive regulation of glucose import [GO:0046326]; positive regulation of insulin receptor signaling pathway [GO:0046628]; positive regulation of phosphatidylinositol 3-kinase/protein kinase B signal transduction [GO:0051897]; protein localization to nuclear pore [GO:0090204]	cortical endoplasmic reticulum [GO:0032541]; cytosol [GO:0005829]; endoplasmic reticulum [GO:0005783]; endoplasmic reticulum membrane [GO:0005789]; membrane [GO:0016020]; nuclear membrane [GO:0031965]	cholesterol binding [GO:0015485]; phosphatidylinositol-4-phosphate binding [GO:0070273]; phosphatidylserine binding [GO:0001786]; phosphatidylserine transfer activity [GO:0140343]; phospholipid transporter activity [GO:0005548]; sterol transporter activity [GO:0015248]
OSBPL9	114883	ENSG000001 17859	Q96SU4	OSBP	Oxysterol-binding protein-related protein 9 (ORP-9) (OSBP-related protein 9)	bile acid biosynthetic process [GO:0006699]	cytosol [GO:0005829]; Golgi apparatus [GO:0005794]; intracellular membrane-bounded organelle [GO:0043231]; late endosome membrane [GO:0031902]; membrane [GO:0016020]	sterol binding [GO:0032934]; sterol transporter activity [GO:0015248]
STARD7	56910	ENSG0000008 4090	Q9NQZ5	START	StAR-related lipid transfer protein 7, mitochondrial (Gestational trophoblastic tumor protein 1) (START domain-containing protein 7) (StARD7)		mitochondrial outer membrane [GO:0005741]	lipid binding [GO:0008289]
STARD8	9754	ENSG0000013 0052	Q92502	START	StAR-related lipid transfer protein 8 (Deleted in liver cancer 3 protein) (DLC-3) (START domain-containing protein 8) (StARD8) (START-GAP3)	actin cytoskeleton organization [GO:0030036]; regulation of Rho protein signal transduction [GO:0035023]; signal transduction [GO:0007165]	focal adhesion [GO:0005925]	GTPase activator activity [GO:0005096]; lipid binding [GO:0008289]

STARD9	57519	ENSG000001 59433	Q9P2P6	START	StAR-related lipid transfer protein 9 (START domain-containing protein 9) (StARD9)	microtubule-based movement [GO:0007018]; spindle assembly [GO:0051225]	centriole [GO:0005814]; cytoplasm [GO:0005737]; nucleus [GO:0005634]	ATP binding [GO:0005524]; lipid binding [GO:0008289]; microtubule binding [GO:0008017]; microtubule motor activity [GO:0003777]
STARD10	10809	ENSG00000214530	Q9Y365	START	START domain-containing protein 10 (StARD10) (Antigen NY-CO-28) (PCTP-like protein) (PCTP-L) (Serologically defined colon cancer antigen 28) (StAR-related lipid transfer protein 10)	lipid transport [GO:0006869]	cytosol [GO:0005829]; intercellular canaliculus [GO:0046581]; membrane [GO:0016020]; microvillus [GO:0005902]; motile cilium [GO:0031514]	lipid binding [GO:0008289]
DLC1	10395	ENSG00000164741	Q96QB1	START	Rho GTPase-activating protein 7 (Deleted in liver cancer 1 protein) (DLC-1) (HP protein) (Rho-type GTPase-activating protein 7) (START domain-containing protein 12) (StARD12) (StAR-related lipid transfer protein 12)	actin cytoskeleton organization [GO:0030036]; activation of cysteine-type endopeptidase activity involved in apoptotic process [GO:0006919]; apoptotic process [GO:0006915]; focal adhesion assembly [GO:0048041]; forebrain development [GO:0030900]; heart morphogenesis [GO:0003007]; hindbrain morphogenesis [GO:0021575]; negative regulation of cell migration [GO:0030336]; negative regulation of cell population proliferation [GO:0008285]; negative regulation of focal adhesion assembly [GO:0051895]; negative regulation of Rho protein signal transduction [GO:0035024]; negative regulation of stress fiber assembly [GO:0051497]; neural tube closure [GO:0001843]; positive regulation of execution phase of apoptosis [GO:1900119]; positive regulation of protein dephosphorylation [GO:0035307]; regulation of actin cytoskeleton organization [GO:0032956]; regulation of cell shape [GO:0008360]; regulation of Rho protein signal transduction [GO:0035023]; regulation of small GTPase mediated signal transduction [GO:0051056]; signal transduction [GO:0007165]	caveola [GO:0005901]; cortical actin cytoskeleton [GO:0030864]; cytoplasm [GO:0005737]; cytosol [GO:0005829]; endoplasmic reticulum [GO:0005783]; focal adhesion [GO:0005925]; intracellular membrane-bounded organelle [GO:0043231]; membrane raft [GO:0045121]; nucleus [GO:0005634]; ruffle membrane [GO:0032587]	GTPase activator activity [GO:0005096]; lipid binding [GO:0008289]; SH2 domain binding [GO:0042169]

PITPNC1	26207	ENSG00000154217	Q9UKF7	PITP	Cytoplasmic phosphatidylinositol transfer protein 1 (Mammalian rdgB homolog beta) (M-rdgB beta) (MrdgBbeta) (Retinal degeneration B homolog beta) (RdgBbeta)	phospholipid transport [GO:0015914]; signal transduction [GO:0007165]	cytoplasm [GO:0005737]; cytosol [GO:0005829]; nucleoplasm [GO:0005654]	phosphatidic acid binding [GO:0070300]; phosphatidic acid transfer activity [GO:1990050]; phosphatidylglycerol binding [GO:1901611]; phosphatidylinositol binding [GO:0035091]; phosphatidylinositol transfer activity [GO:0008526]
GLTPD2	388323	ENSG000001 82327	A6NH11	GLTP	Glycolipid transfer protein domain-containing protein 2	ceramide transport [GO:0035627]; intermembrane lipid transfer [GO:0120009]	cytosol [GO:0005829]	ceramide 1-phosphate binding [GO:1902387]; ceramide 1-phosphate transfer activity [GO:1902388]
SEC14L1	6397	ENSG000001 29657	Q92503	CRALTRIO	SEC14-like protein 1	choline transport [GO:0015871]; innate immune response [GO:0045087]; negative regulation of RIG-I signaling pathway [GO:0039536]	cytoplasm [GO:0005737]; cytosol [GO:0005829]; Golgi apparatus [GO:0005794]; nucleoplasm [GO:0005654]	protein sequestering activity [GO:0140311]; RIG-I binding [GO:0039552]
TTPA	7274	ENSG00000137561	P49638	CRALTRIO	Alpha-tocopherol transfer protein (Alpha-TTP)	embryonic placenta development [GO:0001892]; intermembrane lipid transfer [GO:0120009]; lipid metabolic process [GO:0006629]; negative regulation of establishment of blood-brain barrier [GO:0090212]; positive regulation of amyloid-beta clearance [GO:1900223]; response to toxic substance [GO:0009636]; vitamin E metabolic process [GO:0042360]; vitamin transport [GO:0051180]	cytosol [GO:0005829]; late endosome [GO:0005770]; membrane [GO:0016020]	lipid transfer activity [GO:0120013]; phosphatidylinositol bisphosphate binding [GO:1902936]; phosphatidylinositol-3,4-bisphosphate binding [GO:0043325]; phosphatidylinositol-4,5-bisphosphate binding [GO:0005546]; vitamin E binding [GO:0008431]
GDAP2	54834	ENSG000001 96505	Q9NXN4	CRALTRIO	Ganglioside-induced differentiation-associated protein 2	response to retinoic acid [GO:0032526]	lysosomal membrane [GO:0005765]	

SESTD1	91404	ENSG00000187231	Q86VW0	CRALTRIO	SEC14 domain and spectrin repeat-containing protein 1 (Huntingtin-interacting protein-like protein) (Protein Solo)	negative regulation of calcium ion transmembrane transport via high voltage-gated calcium channel [GO:1904878]	intermediate filament cytoskeleton [GO:0045111]	phosphatidic acid binding [GO:0070300]; phosphatidylinositol-3,4-bisphosphate binding [GO:0043325]; phosphatidylinositol-3,5-bisphosphate binding [GO:0080025]; phosphatidylinositol-3-phosphate binding [GO:0032266]; phosphatidylinositol-4,5-bisphosphate binding [GO:0005546]; phosphatidylinositol-4-phosphate binding [GO:0070273]; phosphatidylinositol-5-phosphate binding [GO:0010314]
MOSPD2	158747	ENSG00000130150	Q8NHP6	CRALTRIO	Motile sperm domain-containing protein 2	chemotaxis [GO:0006935]; lipid droplet formation [GO:0140042]; positive regulation of monocyte chemotaxis [GO:0090026]; positive regulation of neutrophil chemotaxis [GO:0090023]; protein homooligomerization [GO:0051260]	endomembrane system [GO:0012505]; endoplasmic reticulum [GO:0005783]; endoplasmic reticulum membrane [GO:0005789]; endoplasmic reticulum-endosome membrane contact site [GO:0140284]; membrane [GO:0016020]; organelle membrane contact site [GO:0044232]; plasma membrane [GO:0005886]; specific granule membrane [GO:0035579]	FFAT motif binding [GO:0033149]
TEX2	55852	ENSG000001 36478	Q8IWB9	SMP	Testis-expressed protein 2 (Transmembrane protein 96)	lipid transport [GO:0006869]; signal transduction [GO:0007165]; sphingolipid metabolic process [GO:0006665]	endoplasmic reticulum [GO:0005783]; endoplasmic reticulum membrane [GO:0005789]; membrane [GO:0016020]; nuclear membrane [GO:0031965]	lipid binding [GO:0008289]
VPS13D	55187	ENSG000000 48707	Q5THJ4	VPS13	Intermembrane lipid transfer protein VPS13D (Vacuolar protein sorting-associated protein 13D)	lipid transport [GO:0006869]; mitochondrion organization [GO:0007005]; positive regulation of mitophagy [GO:1901526]; protein retention in Golgi apparatus [GO:0045053]; protein targeting to vacuole [GO:0006623]	extracellular exosome [GO:0070062]; extrinsic component of membrane [GO:0019898]	

COL4A3BP	10087	ENSG00000113163	Q9Y5P4	START	Ceramide transfer protein (hCERT) (Collagen type IV alpha-3-binding protein) (Goodpasture antigen-binding protein) (GPBP) (START domain-containing protein 11) (StARD11) (StAR-related lipid transfer protein 11)	cell morphogenesis [GO:0000902]; cell population proliferation [GO:0008283]; ceramide metabolic process [GO:0006672]; ceramide transport [GO:0035627]; endoplasmic reticulum organization [GO:0007029]; ER to Golgi ceramide transport [GO:0035621]; heart morphogenesis [GO:0003007]; immune response [GO:0006955]; in utero embryonic development [GO:0001701]; intermembrane lipid transfer [GO:0120009]; intermembrane sphingolipid transfer [GO:0120012]; lipid homeostasis [GO:0055088]; mitochondrion organization [GO:0007005]; muscle contraction [GO:0006936]; response to endoplasmic reticulum stress [GO:0034976]; signal transduction [GO:0007165]	cytosol [GO:0005829]; endoplasmic reticulum membrane [GO:0005789]; Golgi apparatus [GO:0005794]; mitochondrion [GO:0005739]; nucleoplasm [GO:0005654]	ceramide 1-phosphate binding [GO:1902387]; ceramide 1-phosphate transfer activity [GO:1902388]; ceramide binding [GO:0097001]; ceramide transfer activity [GO:0120017]; identical protein binding [GO:0042802]; kinase activity [GO:0016301]; phosphatidylinositol-4-phosphate binding [GO:0070273]
OSBPL1A	114876	ENSG0000014 1447	Q9BXW6	OSBP	Oxysterol-binding protein-related protein 1 (ORP-1) (OSBP-related protein 1)	bile acid biosynthetic process [GO:0006699]; cholesterol metabolic process [GO:0008203]; vesicle-mediated transport [GO:0016192]	cytosol [GO:0005829]; endosome [GO:0005768]; extracellular exosome [GO:0070062]; late endosome [GO:0005770]; organelle membrane contact site [GO:0044232]; perinuclear endoplasmic reticulum [GO:0097038]; plasma membrane [GO:0005886]	cholesterol binding [GO:0015485]; phospholipid binding [GO:0005543]; sterol transporter activity [GO:0015248]
OSBPL2	9885	ENSG0000013 0703	Q9H1P3	OSBP	Oxysterol-binding protein-related protein 2 (ORP-2) (OSBP-related protein 2)	bile acid biosynthetic process [GO:0006699]; cholesterol transport [GO:0030301]; intracellular cholesterol transport [GO:0032367]; phospholipid transport [GO:0015914]; plasma membrane organization [GO:0007009]; protein homotetramerization [GO:0051289]	cytoplasmic side of plasma membrane [GO:0009898]; cytosol [GO:0005829]; lipid droplet [GO:0005811]; perinuclear endoplasmic reticulum [GO:0097038]; plasma membrane [GO:0005886]	cholesterol binding [GO:0015485]; cholesterol transfer activity [GO:0120020]; phosphatidylinositol transfer activity [GO:0008526]; phosphatidylinositol-4,5-bisphosphate binding [GO:0005546]; sterol transporter activity [GO:0015248]
OSBPL3	26031	ENSG0000007 0882	Q9H4L5	OSBP	Oxysterol-binding protein-related protein 3 (ORP-3) (OSBP-related protein 3)	bile acid biosynthetic process [GO:0006699]	cytosol [GO:0005829]; endoplasmic reticulum membrane [GO:0005789]; filopodium tip [GO:0032433]; membrane [GO:0016020]; nuclear membrane [GO:0031965]; perinuclear endoplasmic reticulum [GO:0097038]; plasma membrane [GO:0005886]	cholesterol binding [GO:0015485]; sterol transporter activity [GO:0015248]
OSBP2	23762	ENSG000001 84792	Q969R2	OSBP	Oxysterol-binding protein 2 (Oxysterol-binding protein-related protein 4) (ORP-4) (OSBP-related protein 4)	localization [GO:0051179]; spermatid development [GO:0007286]	apical dendrite [GO:0097440]; cytosol [GO:0005829]; perinuclear endoplasmic reticulum [GO:0097038]; plasma membrane [GO:0005886]	cholesterol binding [GO:0015485]; sterol transporter activity [GO:0015248]

OSBPL5	114879	ENSG00000021762	Q9H0X9	OSBP	Oxysterol-binding protein-related protein 5 (ORP-5) (OSBP-related protein 5) (Oxysterol-binding protein homolog 1)	cholesterol metabolic process [GO:0008203]; cholesterol transport [GO:0030301]; Golgi to plasma membrane transport [GO:0006893]; phosphatidylserine acyl-chain remodeling [GO:0036150]; phospholipid transport [GO:0015914]	cytosol [GO:0005829]; endoplasmic reticulum membrane [GO:0005789]; endoplasmic reticulum-plasma membrane contact site [GO:0140268]; intracellular membrane-bounded organelle [GO:0043231]; membrane [GO:0016020]	cholesterol binding [GO:0015485]; oxysterol binding [GO:0008142]; phosphatidylinositol-4-phosphate binding [GO:0070273]; phosphatidylserine binding [GO:0001786]; phosphatidylserine transfer activity [GO:0140343]; phospholipid transporter activity [GO:0005548]; sterol binding [GO:0032934]; sterol transporter activity [GO:0015248]
OSBPL6	114880	ENSG0000007 9156	Q9BZF3	OSBP	Oxysterol-binding protein-related protein 6 (ORP-6) (OSBP-related protein 6)	bile acid biosynthetic process [GO:0006699]; regulation of cholesterol transport [GO:0032374]	cytosol [GO:0005829]; early endosome membrane [GO:0031901]; endoplasmic reticulum membrane [GO:0005789]; nuclear membrane [GO:0031965]; perinuclear endoplasmic reticulum [GO:0097038]; plasma membrane [GO:0005886]	cholesterol binding [GO:0015485]; sterol transporter activity [GO:0015248]
OSBPL7	114881	ENSG0000000 6025	Q9BZF2	OSBP	Oxysterol-binding protein-related protein 7 (ORP-7) (OSBP-related protein 7)	bile acid biosynthetic process [GO:0006699]; cellular response to cholesterol [GO:0071397]; positive regulation of proteasomal protein catabolic process [GO:1901800]; regulation of autophagy [GO:0010506]	autophagosome [GO:0005776]; cytosol [GO:0005829]; endoplasmic reticulum membrane [GO:0005789]; nuclear membrane [GO:0031965]; nucleoplasm [GO:0005654]; perinuclear endoplasmic reticulum [GO:0097038]; plasma membrane [GO:0005886]	cholesterol binding [GO:0015485]; sterol transporter activity [GO:0015248]
OSBPL10	114884	ENSG000001 44645	Q9BXB5	OSBP	Oxysterol-binding protein-related protein 10 (ORP-10) (OSBP-related protein 10)	phosphatidylserine acyl-chain remodeling [GO:0036150]	cytoskeleton [GO:0005856]; cytosol [GO:0005829]; intracellular membrane-bounded organelle [GO:0043231]; membrane [GO:0016020]	cholesterol binding [GO:0015485]; phosphatidylserine binding [GO:0001786]; phospholipid transporter activity [GO:0005548]; sterol transporter activity [GO:0015248]
STARD3	10948	ENSG00000131748	Q14849	START	StAR-related lipid transfer protein 3 (Metastatic lymph node gene 64 protein) (MLN 64) (Protein CAB1) (START domain-containing protein 3) (StARD3)	cholesterol metabolic process [GO:0008203]; cholesterol transport [GO:0030301]; lipid metabolic process [GO:0006629]; mitochondrial transport [GO:0006839]; progesterone biosynthetic process [GO:0006701]; steroid metabolic process [GO:0008202]; vesicle tethering to endoplasmic reticulum [GO:0099044]	cytoplasm [GO:0005737]; cytosol [GO:0005829]; endoplasmic reticulum membrane [GO:0005789]; endoplasmic reticulum-endosome membrane contact site [GO:0140284]; endosome [GO:0005768]; intracellular membrane-bounded organelle [GO:0043231]; late endosome membrane [GO:0031902]; lysosomal membrane [GO:0005765]; mitochondrion [GO:0005739]; nucleoplasm [GO:0005654]; organelle membrane contact site [GO:0044232]	cholesterol binding [GO:0015485]; cholesterol transfer activity [GO:0120020]; protein homodimerization activity [GO:0042803]
STARD4	134429	ENSG000001642 11	Q96DR4	START	StAR-related lipid transfer protein 4 (START domain-containing protein 4) (StARD4)	cholesterol import [GO:0070508]; cholesterol transport involved in cholesterol storage [GO:0010879]; intracellular cholesterol transport [GO:0032367]; positive regulation of bile acid biosynthetic process [GO:0070859]; positive regulation of cholesterol metabolic process [GO:0090205]	cytoplasm [GO:0005737]; cytoplasmic vesicle [GO:0031410]; cytosol [GO:0005829]; endoplasmic reticulum [GO:0005783]	cholesterol binding [GO:0015485]; cholesterol transfer activity [GO:0120020]

STARD5	80765	ENSG000001 72345	Q9NSY2	START	StAR-related lipid transfer protein 5 (START domain-containing protein 5) (StARD5)	cholesterol import [GO:0070508]	cytosol [GO:0005829]	cholesterol binding [GO:0015485]; cholesterol transfer activity [GO:0120020]
STARD6	147323	ENSG000001 74448	P59095	START	StAR-related lipid transfer protein 6 (START domain-containing protein 6) (StARD6)	lipid transport [GO:0006869]		lipid binding [GO:0008289]
STARD13	90627	ENSG00000133121	Q9Y3M8	START	StAR-related lipid transfer protein 13 (46H23.2) (Deleted in liver cancer 2 protein) (DLC-2) (Rho GTPase-activating protein) (START domain-containing protein 13) (StARD13)	actin cytoskeleton organization [GO:0030036]; endothelial cell migration [GO:0043542]; endothelial tube lumen extension [GO:0097498]; negative regulation of cell migration involved in sprouting angiogenesis [GO:0090051]; regulation of Rho protein signal transduction [GO:0035023]; regulation of small GTPase mediated signal transduction [GO:0051056]; signal transduction [GO:0007165]	cytosol [GO:0005829]; lipid droplet [GO:0005811]; mitochondrial membrane [GO:0031966]	GTPase activator activity [GO:0005096]; lipid binding [GO:0008289]
ACOT11	26027	ENSG00000162390	Q8WXI4	START	Acyl-coenzyme A thioesterase 11 (Acyl-CoA thioesterase 11) (EC 3.1.2.-) (Acyl-CoA thioester hydrolase 11) (Adipose-associated thioesterase) (Brown fat-inducible thioesterase) (BFIT) (Palmitoyl-coenzyme A thioesterase) (EC 3.1.2.2)	acyl-CoA metabolic process [GO:0006637]; fatty acid metabolic process [GO:0006631]; intracellular signal transduction [GO:0035556]; negative regulation of cold-induced thermogenesis [GO:0120163]; response to cold [GO:0009409]; response to temperature stimulus [GO:0009266]	cytoplasm [GO:0005737]; cytosol [GO:0005829]; extracellular exosome [GO:0070062]; mitochondrial matrix [GO:0005759]	carboxylic ester hydrolase activity [GO:0052689]; fatty acyl-CoA hydrolase activity [GO:0047617]; lipid binding [GO:0008289]; long-chain fatty acyl-CoA hydrolase activity [GO:0052816]
ACOT12	134526	ENSG00000172497	Q8WYK0	START	Acetyl-coenzyme A thioesterase (EC 3.1.2.1) (Acyl-CoA thioester hydrolase 12) (Acyl-coenzyme A thioesterase 12) (Acyl-CoA thioesterase 12) (Cytoplasmic acetyl-CoA hydrolase 1) (CACH-1) (hCACH-1) (START domain-containing protein 15) (StARD15)	acetyl-CoA metabolic process [GO:0006084]; acyl-CoA metabolic process [GO:0006637]; fatty acid metabolic process [GO:0006631]	cytoplasm [GO:0005737]; cytosol [GO:0005829]; extracellular exosome [GO:0070062]; intercellular bridge [GO:0045171]; nucleoplasm [GO:0005654]	acetyl-CoA hydrolase activity [GO:0003986]; ATP binding [GO:0005524]; carboxylic ester hydrolase activity [GO:0052689]; fatty acyl-CoA hydrolase activity [GO:0047617]; identical protein binding [GO:0042802]; lipid binding [GO:0008289]

NPC1	4864	ENSG00000141458	O15118	NPC1 NTD	NPC intracellular cholesterol transporter 1 (Niemann-Pick C1 protein)	adult walking behavior [GO:0007628]; autophagy [GO:0006914]; bile acid metabolic process [GO:0008206]; cellular response to low-density lipoprotein particle stimulus [GO:0071404]; cellular response to steroid hormone stimulus [GO:0071383]; cholesterol efflux [GO:0033344]; cholesterol homeostasis [GO:0042632]; cholesterol metabolic process [GO:0008203]; cholesterol storage [GO:0010878]; cholesterol transport [GO:0030301]; cyclodextrin metabolic process [GO:2000900]; endocytosis [GO:0006897]; establishment of protein localization to membrane [GO:0090150]; gene expression [GO:0010467]; intestinal cholesterol absorption [GO:0030299]; intracellular cholesterol transport [GO:0032367]; intracellular lipid transport [GO:0032365]; liver development [GO:0001889]; lysosomal transport [GO:0007041]; macroautophagy [GO:0016236]; membrane raft organization [GO:0031579]; negative regulation of epithelial cell apoptotic process [GO:1904036]; negative regulation of macroautophagy [GO:0016242]; negative regulation of TORC1 signaling [GO:1904262]; neurogenesis [GO:0022008]; programmed cell death [GO:0012501]; protein glycosylation [GO:0006486]; response to cadmium ion [GO:0046686]; response to xenobiotic stimulus [GO:0009410]; symbiont entry into host cell [GO:0046718]	endoplasmic reticulum [GO:0005783]; extracellular exosome [GO:0070062]; extracellular region [GO:0005576]; Golgi apparatus [GO:0005794]; late endosome membrane [GO:0031902]; lysosomal membrane [GO:0005765]; lysosome [GO:0005764]; membrane [GO:0016020]; membrane raft [GO:0045121]; nuclear envelope [GO:0005635]; perinuclear region of cytoplasm [GO:0048471]; plasma membrane [GO:0005886]	cholesterol binding [GO:0015485]; signaling receptor activity [GO:0038023]; sterol transporter activity [GO:0015248]; transmembrane signaling receptor activity [GO:0004888]; virus receptor activity [GO:0001618]
NPC1L1	29881	ENSG00000015520	Q9UHC9	NPC1 NTD	NPC1-like intracellular cholesterol transporter 1 (NPC1L1) (Niemann-Pick C1-like protein 1)	cellular response to sterol depletion [GO:0071501]; cholesterol biosynthetic process [GO:0006695]; cholesterol homeostasis [GO:0042632]; cholesterol transport [GO:0030301]; intestinal cholesterol absorption [GO:0030299]; lipoprotein metabolic process [GO:0042157]; vitamin E metabolic process [GO:0042360]; vitamin transport [GO:0051180]	apical plasma membrane [GO:0016324]; cytoplasmic vesicle membrane [GO:0030659]; plasma membrane [GO:0005886]	cholesterol binding [GO:0015485]; myosin V binding [GO:0031489]; protein homodimerization activity [GO:0042803]; small GTPase binding [GO:0031267]; vitamin E binding [GO:0008431]

NPC2	10577	ENSG00000119655	P61916	ML	NPC intracellular cholesterol transporter 2 (Epididymal secretory protein E1) (Human epididymis-specific protein 1) (He1) (Niemann-Pick disease type C2 protein)	cholesterol efflux [GO:0033344]; cholesterol homeostasis [GO:0042632]; cholesterol metabolic process [GO:0008203]; cholesterol storage [GO:0010878]; cholesterol transport [GO:0030301]; gene expression [GO:0010467]; glycolipid transport [GO:0046836]; intracellular cholesterol transport [GO:0032367]; intracellular sterol transport [GO:0032366]; phospholipid transport [GO:0015914]; regulation of isoprenoid metabolic process [GO:0019747]; response to virus [GO:0009615]	azurophil granule lumen [GO:0035578]; endoplasmic reticulum [GO:0005783]; extracellular exosome [GO:0070062]; extracellular region [GO:0005576]; extracellular space [GO:0005615]; lysosomal lumen [GO:0043202]; lysosome [GO:0005764]	cholesterol binding [GO:0015485]; cholesterol transfer activity [GO:0120020]; enzyme binding [GO:0019899]
LY86	9450	ENSG0000011 2799	O95711	ML	Lymphocyte antigen 86 (Ly-86) (Protein MD-1)	inflammatory response [GO:0006954]; innate immune response [GO:0045087]; lipopolysaccharide-mediated signaling pathway [GO:0031663]; positive regulation of lipopolysaccharide-mediated signaling pathway [GO:0031666]	extracellular region [GO:0005576]	
LY96	23643	ENSG00000154589	Q9Y6Y9	ML	Lymphocyte antigen 96 (Ly-96) (ESOP-1) (Protein MD-2)	cell surface receptor signaling pathway [GO:0007166]; cellular defense response [GO:0006968]; cellular response to lipopolysaccharide [GO:0071222]; detection of lipopolysaccharide [GO:0032497]; inflammatory response [GO:0006954]; innate immune response [GO:0045087]; positive regulation of lipopolysaccharide-mediated signaling pathway [GO:0031666]; positive regulation of tumor necrosis factor production [GO:0032760]; response to lipopolysaccharide [GO:0032496]; toll-like receptor 4 signaling pathway [GO:0034142]; toll-like receptor signaling pathway [GO:0002224]	endosome membrane [GO:0010008]; extracellular region [GO:0005576]; lipopolysaccharide receptor complex [GO:0046696]; plasma membrane [GO:0005886]; receptor complex [GO:0043235]	coreceptor activity [GO:0015026]; lipopolysaccharide binding [GO:0001530]; lipopolysaccharide immune receptor activity [GO:0001875]; Toll-like receptor 4 binding [GO:0035662]
GM2A	2760	ENSG00000196743	P17900	ML	Ganglioside GM2 activator (Cerebroside sulfate activator protein) (GM2-AP) (Sphingolipid activator protein 3) (SAP-3) [Cleaved into: Ganglioside GM2 activator isoform short]	ganglioside catabolic process [GO:0006689]; glycosphingolipid catabolic process [GO:0046479]; learning or memory [GO:0007611]; lipid storage [GO:0019915]; lipid transport [GO:0006869]; maintenance of location in cell [GO:0051651]; neuromuscular process controlling balance [GO:0050885]; oligosaccharide catabolic process [GO:0009313]	apical plasma membrane [GO:0016324]; azurophil granule lumen [GO:0035578]; basolateral plasma membrane [GO:0016323]; cytoplasmic side of plasma membrane [GO:0009898]; cytosol [GO:0005829]; extracellular exosome [GO:0070062]; extracellular region [GO:0005576]; intracellular membrane-bounded organelle [GO:0043231]; lysosomal lumen [GO:0043202]	beta-N-acetylgalactosaminidase activity [GO:0032428]; lipid transporter activity [GO:0005319]; phospholipase activator activity [GO:0016004]; sphingolipid activator protein activity [GO:0030290]

PITPNM1	9600	ENSG00000110697	O00562	PITP	Membrane-associated phosphatidylinositol transfer protein 1 (Drosophila retinal degeneration B homolog) (Phosphatidylinositol transfer protein, membrane-associated 1) (PITPnm 1) (Pyk2 N-terminal domain-interacting receptor 2) (NIR-2)	brain development [GO:0007420]; lipid metabolic process [GO:0006629]; phosphatidylinositol biosynthetic process [GO:0006661]; phospholipid transport [GO:0015914]; phototransduction [GO:0007602]; protein transport [GO:0015031]	cell body [GO:0044297]; cleavage furrow [GO:0032154]; cytoplasm [GO:0005737]; cytosol [GO:0005829]; endoplasmic reticulum membrane [GO:0005789]; Golgi cisterna membrane [GO:0032580]; intracellular membrane-bounded organelle [GO:0043231]; lipid droplet [GO:0005811]; membrane [GO:0016020]; midbody [GO:0030496]	calcium ion binding [GO:0005509]; phosphatidic acid binding [GO:0070300]; phosphatidylcholine binding [GO:0031210]; phosphatidylcholine transporter activity [GO:0008525]; phosphatidylinositol binding [GO:0035091]; phosphatidylinositol transfer activity [GO:0008526]; receptor tyrosine kinase binding [GO:0030971]
PITPNM2	57605	ENSG00000090975	Q9BZ72	PITP	Membrane-associated phosphatidylinositol transfer protein 2 (Phosphatidylinositol transfer protein, membrane-associated 2) (PITPnm 2) (Pyk2 N-terminal domain-interacting receptor 3) (NIR-3)	phosphatidylinositol biosynthetic process [GO:0006661]	cytoplasm [GO:0005737]; cytosol [GO:0005829]; endomembrane system [GO:0012505]; membrane [GO:0016020]	calcium ion binding [GO:0005509]; phosphatidylcholine binding [GO:0031210]; phosphatidylcholine transporter activity [GO:0008525]; phosphatidylinositol binding [GO:0035091]; phosphatidylinositol transfer activity [GO:0008526]; receptor tyrosine kinase binding [GO:0030971]
PITPNB	23760	ENSG00000180957	P48739	PITP	Phosphatidylinositol transfer protein beta isoform (PI-TP-beta) (PtdIns transfer protein beta) (PtdInsTP beta)	lipid metabolic process [GO:0006629]; nucleus organization [GO:0006997]; phospholipid transport [GO:0015914]; retrograde vesicle-mediated transport, Golgi to endoplasmic reticulum [GO:0006890]	cytoplasm [GO:0005737]; endoplasmic reticulum membrane [GO:0005789]; Golgi apparatus [GO:0005794]; Golgi membrane [GO:0000139]	phosphatidylcholine binding [GO:0031210]; phosphatidylcholine transfer activity [GO:0120019]; phosphatidylcholine transporter activity [GO:0008525]; phosphatidylinositol binding [GO:0035091]; phosphatidylinositol transfer activity [GO:0008526]; sphingomyelin transfer activity [GO:0140338]
PITPNA	5306	ENSG00000174238	Q00169	PITP	Phosphatidylinositol transfer protein alpha isoform (PI-TP-alpha) (PtdIns transfer protein alpha) (PtdInsTP alpha)	axonogenesis [GO:0007409]; lipid metabolic process [GO:0006629]; phospholipid transport [GO:0015914]; visual perception [GO:0007601]	cytoplasm [GO:0005737]; cytosol [GO:0005829]; extracellular exosome [GO:0070062]; nucleus [GO:0005634]	lipid binding [GO:0008289]; phosphatidylcholine binding [GO:0031210]; phosphatidylcholine transfer activity [GO:0120019]; phosphatidylcholine transporter activity [GO:0008525]; phosphatidylglycerol binding [GO:1901611]; phosphatidylinositol binding [GO:0035091]; phosphatidylinositol transfer activity [GO:0008526]

PITPNM3	83394	ENSG00000091622	Q9BZ71	PITP	Membrane-associated phosphatidylinositol transfer protein 3 (Phosphatidylinositol transfer protein, membrane-associated 3) (PITPnm 3) (Pyk2 N-terminal domain-interacting receptor 1) (NIR-1)	phosphatidylinositol biosynthetic process [GO:0006661]	cell body [GO:0044297]; cell projection [GO:0042995]; COPII-coated ER to Golgi transport vesicle [GO:0030134]; cytoplasm [GO:0005737]; cytosol [GO:0005829]; endomembrane system [GO:0012505]; membrane [GO:0016020]	calcium ion binding [GO:0005509]; lipid binding [GO:0008289]; phosphatidylinositol transfer activity [GO:0008526]; phospholipase activity [GO:0004620]; receptor tyrosine kinase binding [GO:0030971]
SCP2	6342	ENSG00000116171	P22307	SCP2	Sterol carrier protein 2 (SCP-2) (Acetyl-CoA C-myristoyltransferase) (EC 2.3.1.155) (Non-specific lipid-transfer protein) (NSL-TP) (Propanoyl-CoA C-acyltransferase) (EC 2.3.1.176) (SCP-2/3-oxoacyl-CoA thiolase) (SCP-2/thiolase) (EC 2.3.1.16) (SCP-chi) (SCPX) (Sterol carrier protein X) (SCP-X)	alpha-linolenic acid metabolic process [GO:0036109]; bile acid biosynthetic process [GO:0006699]; bile acid metabolic process [GO:0008206]; fatty acid beta-oxidation [GO:0006635]; fatty acid beta-oxidation using acyl-CoA oxidase [GO:0033540]; inositol trisphosphate biosynthetic process [GO:0032959]; intracellular cholesterol transport [GO:0032367]; lipid hydroperoxide transport [GO:1901373]; phospholipid transport [GO:0015914]; positive regulation of intracellular cholesterol transport [GO:0032385]; positive regulation of steroid metabolic process [GO:0045940]; progesterone biosynthetic process [GO:0006701]; protein localization to plasma membrane [GO:0072659]; regulation of phospholipid biosynthetic process [GO:0071071]; steroid biosynthetic process [GO:0006694]	cytoplasm [GO:0005737]; cytosol [GO:0005829]; endoplasmic reticulum [GO:0005783]; membrane [GO:0016020]; mitochondrion [GO:0005739]; nucleoplasm [GO:0005654]; peroxisomal matrix [GO:0005782]; peroxisome [GO:0005777]; protein-containing complex [GO:0032991]	acetyl-CoA C-acyltransferase activity [GO:0003988]; acetyl-CoA C-myristoyltransferase activity [GO:0050633]; cholesterol binding [GO:0015485]; cholesterol transfer activity [GO:0120020]; fatty-acyl-CoA binding [GO:0000062]; long-chain fatty acyl-CoA binding [GO:0036042]; oleic acid binding [GO:0070538]; phosphatidylcholine transfer activity [GO:0120019]; phosphatidylinositol transfer activity [GO:0008526]; propanoyl-CoA C-acyltransferase activity [GO:0033814]; propionyl-CoA C2-trimethyltridecanoyltransferase activity [GO:0050632]; signaling receptor binding [GO:0005102]
SCP2D1	140856	ENSG00000132631	Q9UJQ7	SCP2	SCP2 sterol-binding domain-containing protein 1		cytosol [GO:0005829]	

HSD17B4	3295	ENSG00000133835	P51659	SCP2	Peroxisomal multifunctional enzyme type 2 (MFE-2) (17-beta-hydroxysteroid dehydrogenase 4) (17-beta-HSD 4) (D-bifunctional protein) (DBP) (Multifunctional protein 2) (MFP-2) (Short chain dehydrogenase/reductase family 8C member 1) [Cleaved into: (3R)-hydroxyacyl-CoA dehydrogenase (EC 1.1.1.n12); Enoyl-CoA hydratase 2 (EC 4.2.1.107) (EC 4.2.1.119) (3-alpha,7-alpha,12-alpha-trihydroxy-5-beta-cholest-24-enoyl-CoA hydratase)]	androgen metabolic process [GO:0008209]; estrogen metabolic process [GO:0008210]; fatty acid beta-oxidation [GO:0006635]; medium-chain fatty-acyl-CoA metabolic process [GO:0036112]; osteoblast differentiation [GO:0001649]; Sertoli cell development [GO:0060009]; very long-chain fatty acid metabolic process [GO:0000038]; very long-chain fatty-acyl-CoA metabolic process [GO:0036111]	cytosol [GO:0005829]; membrane [GO:0016020]; peroxisomal matrix [GO:0005782]; peroxisomal membrane [GO:0005778]; peroxisome [GO:0005777]	(3R)-hydroxyacyl-CoA dehydrogenase (NAD) activity [GO:0106386]; 17-beta-hydroxysteroid dehydrogenase (NAD+) activity [GO:0044594]; 3-hydroxyacyl-CoA dehydratase activity [GO:0018812]; 3-hydroxyacyl-CoA dehydrogenase activity [GO:0003857]; 3alpha,7alpha,12alpha-trihydroxy-5beta-cholest-24-enoyl-CoA hydratase activity [GO:0033989]; enoyl-CoA hydratase activity [GO:0004300]; estradiol 17-beta-dehydrogenase [NAD(P)] activity [GO:0004303]; isomerase activity [GO:0016853]; protein homodimerization activity [GO:0042803]
STOML1	9399	ENSG00000067221	Q9UBI4	SCP2	Stomatin-like protein 1 (SLP-1) (EPB72-like protein 1) (Protein unc-24 homolog) (Stomatin-related protein) (STORP)	lipid transport [GO:0006869]	late endosome membrane [GO:0031902]; membrane raft [GO:0045121]; plasma membrane [GO:0005886]	
HSDL2	84263	ENSG00000119471	Q6YN16	SCP2	Hydroxysteroid dehydrogenase-like protein 2 (EC 1.-.-.-) (Short chain dehydrogenase/reductase family 13C member 1)		membrane [GO:0016020]; mitochondrion [GO:0005739]; peroxisome [GO:0005777]	oxidoreductase activity [GO:0016491]
CPTP	80772	ENSG00000224051	Q5TA50	GLTP	Ceramide-1-phosphate transfer protein (CPTP) (Glycolipid transfer protein domain-containing protein 1) (GLTP domain-containing protein 1)	ceramide 1-phosphate transport [GO:1902389]; ceramide transport [GO:0035627]; intermembrane lipid transfer [GO:0120009]; negative regulation of autophagy [GO:0010507]; negative regulation of interleukin-1 beta production [GO:0032691]; negative regulation of NLRP3 inflammasome complex assembly [GO:1900226]	cytosol [GO:0005829]; endosome membrane [GO:0010008]; Golgi apparatus [GO:0005794]; nuclear outer membrane [GO:0005640]; plasma membrane [GO:0005886]	ceramide 1-phosphate binding [GO:1902387]; ceramide 1-phosphate transfer activity [GO:1902388]; phospholipid binding [GO:0005543]

GLTP	51228	ENSG000001394 33	Q9NZD2	GLTP	Glycolipid transfer protein (GLTP)	ceramide transport [GO:0035627]; ER to Golgi ceramide transport [GO:0035621]; intermembrane lipid transfer [GO:0120009]; response to immobilization stress [GO:0035902]	cytosol [GO:0005829]	ceramide 1-phosphate binding [GO:1902387]; ceramide 1-phosphate transfer activity [GO:1902388]; glycolipid binding [GO:0051861]; glycolipid transfer activity [GO:0017089]; identical protein binding [GO:0042802]; lipid binding [GO:0008289]; lipid transfer activity [GO:0120013]
PLEKHA8	84725	ENSG00000106086	Q96JA3	GLTP	Pleckstrin homology domain-containing family A member 8 (PH domain-containing family A member 8) (Phosphatidylinositol-four-phosphate adapter protein 2) (FAPP-2) (Phosphoinositol 4-phosphate adapter protein 2) (hFAPP2) (Serologically defined breast cancer antigen NY-BR-86)	ceramide transport [GO:0035627]; ER to Golgi ceramide transport [GO:0035621]; intermembrane lipid transfer [GO:0120009]; lipid transport [GO:0006869]; protein transport [GO:0015031]	cytosol [GO:0005829]; Golgi apparatus [GO:0005794]; Golgi membrane [GO:0000139]; nucleoplasm [GO:0005654]; trans-Golgi network [GO:0005802]	ceramide 1-phosphate binding [GO:1902387]; ceramide 1-phosphate transfer activity [GO:1902388]; ceramide binding [GO:0097001]; glycolipid binding [GO:0051861]; glycolipid transfer activity [GO:0017089]; phosphatidylinositol-4-phosphate binding [GO:0070273]
SEC14L2	23541	ENSG0000010 0003	O76054	CRALTRIO	SEC14-like protein 2 (Alpha-tocopherol-associated protein) (TAP) (hTAP) (Squalene transfer protein) (Supernatant protein factor) (SPF)	positive regulation of DNA-templated transcription [GO:0045893]; regulation of cholesterol biosynthetic process [GO:0045540]	cytoplasm [GO:0005737]; cytosol [GO:0005829]; extracellular exosome [GO:0070062]; nucleoplasm [GO:0005654]; nucleus [GO:0005634]	phospholipid binding [GO:0005543]; vitamin E binding [GO:0008431]
SEC14L3	266629	ENSG000001 00012	Q9UDX4	CRALTRIO	SEC14-like protein 3 (Tocopherol-associated protein 2)		cytoplasm [GO:0005737]; extracellular exosome [GO:0070062]; nuclear speck [GO:0016607]	lipid binding [GO:0008289]
SEC14L4	284904	ENSG000001 33488	Q9UDX3	CRALTRIO	SEC14-like protein 4 (Tocopherol-associated protein 3)		cytoplasm [GO:0005737]	lipid binding [GO:0008289]

SEC14L5	9717	ENSG00000103184	O43304	CRALTRIO	SEC14-like protein 5		cytoplasm [GO:0005737]	
SEC14L6	730005	ENSG00000214491	B5MCN3	CRALTRIO	Putative SEC14-like protein 6		cytoplasm [GO:0005737]	
MCF2L	23263	ENSG00000126217	O15068	CRALTRIO	Guanine nucleotide exchange factor DBS (DBL's big sister) (MCF2-transforming sequence-like protein)	intracellular signal transduction [GO:0035556]; positive regulation of Rho protein signal transduction [GO:0035025]; regulation of small GTPase mediated signal transduction [GO:0051056]	cytoplasm [GO:0005737]; cytosol [GO:0005829]; endomembrane system [GO:0012505]; extracellular space [GO:0005615]; extrinsic component of cytoplasmic side of plasma membrane [GO:0031234]; extrinsic component of membrane [GO:0019898]; plasma membrane [GO:0005886]	guanyl-nucleotide exchange factor activity [GO:0005085]; phosphatidylinositol binding [GO:0035091]
MCF2	4168	ENSG00000101977	P10911	CRALTRIO	Proto-oncogene DBL (Proto-oncogene MCF-2) [Cleaved into: MCF2-transforming protein; DBL-transforming protein]	cellular response to leukemia inhibitory factor [GO:1990830]; dendrite development [GO:0016358]; intracellular signal transduction [GO:0035556]; negative regulation of axonogenesis [GO:0050771]; regulation of small GTPase mediated signal transduction [GO:0051056]	cytoplasm [GO:0005737]; cytoskeleton [GO:0005856]; cytosol [GO:0005829]; extrinsic component of membrane [GO:0019898]; membrane [GO:0016020]	guanyl-nucleotide exchange factor activity [GO:0005085]
RLBP1	6017	ENSG00000140522	P12271	CRALTRIO	Retinaldehyde-binding protein 1 (Cellular retinaldehyde-binding protein)	response to stimulus [GO:0050896]; visual perception [GO:0007601]; vitamin A metabolic process [GO:0006776]	cell body [GO:0044297]; centrosome [GO:0005813]; cytosol [GO:0005829]; membrane [GO:0016020]; nucleoplasm [GO:0005654]	11-cis retinal binding [GO:0005502]; phosphatidylinositol bisphosphate binding [GO:1902936]; retinol binding [GO:0019841]
CLVS2	134829	ENSG00000146352	Q5SYC1	CRALTRIO	Clavesin-2 (Retinaldehyde-binding protein 1-like 2) (clathrin vesicle-associated Sec14 protein 2)	lysosome organization [GO:0007040]	clathrin-coated vesicle [GO:0030136]; early endosome membrane [GO:0031901]; endosome [GO:0005768]; trans-Golgi network [GO:0005802]; trans-Golgi network membrane [GO:0032588]	phosphatidylinositol bisphosphate binding [GO:1902936]; phosphatidylinositol-3,5-bisphosphate binding [GO:0080025]
CLVS1	157807	ENSG00000177182	Q8IUQ0	CRALTRIO	Clavesin-1 (Cellular retinaldehyde-binding protein-like) (Retinaldehyde-binding protein 1-like 1) (clathrin vesicle-associated Sec14 protein 1)	lysosome organization [GO:0007040]	clathrin-coated vesicle [GO:0030136]; early endosome membrane [GO:0031901]; endosome [GO:0005768]; trans-Golgi network [GO:0005802]; trans-Golgi network membrane [GO:0032588]	phosphatidylinositol bisphosphate binding [GO:1902936]; phosphatidylinositol-3,5-bisphosphate binding [GO:0080025]

TTPAL	79183	ENSG00000124120	Q9BTX7	CRALTRIO	Alpha-tocopherol transfer protein-like		membrane [GO:0016020]	phosphatidylinositol bisphosphate binding [GO:1902936]

NF1	4763	ENSG00000196712	P21359	CRALTRIO	Neurofibromin (Neurofibromatosis-related protein NF-1) [Cleaved into: Neurofibromin truncated]	actin cytoskeleton organization [GO:0030036]; adrenal gland development [GO:0030325]; amygdala development [GO:0021764]; angiogenesis [GO:0001525]; artery morphogenesis [GO:0048844]; brain development [GO:0007420]; camera-type eye morphogenesis [GO:0048593]; cell communication [GO:0007154]; cellular response to heat [GO:0034605]; cerebral cortex development [GO:0021987]; cognition [GO:0050890]; collagen fibril organization [GO:0030199]; endothelial cell proliferation [GO:0001935]; extracellular matrix organization [GO:0030198]; extrinsic apoptotic signaling pathway in absence of ligand [GO:0097192]; extrinsic apoptotic signaling pathway via death domain receptors [GO:0008625]; fibroblast proliferation [GO:0048144]; forebrain astrocyte development [GO:0021897]; forebrain morphogenesis [GO:0048853]; gamma-aminobutyric acid secretion, neurotransmission [GO:0061534]; glutamate secretion, neurotransmission [GO:0061535]; hair follicle maturation [GO:0048820]; heart development [GO:0007507]; liver development [GO:0001889]; long-term synaptic potentiation [GO:0060291]; MAPK cascade [GO:0000165]; mast cell apoptotic process [GO:0033024]; mast cell proliferation [GO:0070662]; metanephros development [GO:0001656]; myelination in peripheral nervous system [GO:0022011]; myeloid leukocyte migration [GO:0097529]; negative regulation of angiogenesis [GO:0016525]; negative regulation of astrocyte differentiation [GO:0048712]; negative regulation of cell migration [GO:0030336]; negative regulation of cell-matrix adhesion [GO:0001953]; negative regulation of endothelial cell proliferation [GO:0001937]; negative regulation of fibroblast proliferation [GO:0048147]; negative regulation of leukocyte migration [GO:0002686]; negative regulation of MAP kinase activity [GO:0043407]; negative regulation of MAPK cascade [GO:0043409]; negative regulation of mast cell proliferation	axon [GO:0030424]; cytoplasm [GO:0005737]; cytosol [GO:0005829]; dendrite [GO:0030425]; glutamatergic synapse [GO:0098978]; membrane [GO:0016020]; nucleolus [GO:0005730]; nucleoplasm [GO:0005654]; nucleus [GO:0005634]; plasma membrane [GO:0005886]; presynapse [GO:0098793]	GTPase activator activity [GO:0005096]; phosphatidylcholine binding [GO:0031210]; phosphatidylethanolamine binding [GO:0008429]

						[GO:0070667]; negative regulation of neuroblast proliferation [GO:0007406]; negative regulation of neurotransmitter secretion [GO:0046929]; negative regulation of oligodendrocyte differentiation [GO:0048715]; negative regulation of osteoclast differentiation [GO:0045671]; negative regulation of protein import into nucleus [GO:0042308]; negative regulation of protein kinase activity [GO:0006469]; negative regulation of Rac protein signal transduction [GO:0035021]; negative regulation of Ras protein signal transduction [GO:0046580]; negative regulation of Schwann cell migration [GO:1900148]; negative regulation of Schwann cell proliferation [GO:0010626]; negative regulation of stem cell proliferation [GO:2000647]; negative regulation of vascular associated smooth muscle cell migration [GO:1904753]; neural tube development [GO:0021915]; neuroblast proliferation [GO:0007405]; neuron apoptotic process [GO:0051402]; observational learning [GO:0098597]; oligodendrocyte differentiation [GO:0048709]; osteoblast differentiation [GO:0001649]; osteoclast differentiation [GO:0030316]; peripheral nervous system development [GO:0007422]; phosphatidylinositol 3-kinase/protein kinase B signal transduction [GO:0043491]; pigmentation [GO:0043473]; positive regulation of adenylate cyclase activity [GO:0045762]; positive regulation of apoptotic process [GO:0043065]; positive regulation of endothelial cell proliferation [GO:0001938]; positive regulation of extrinsic apoptotic signaling pathway in absence of ligand [GO:2001241]; positive regulation of GTPase activity [GO:0043547]; positive regulation of mast cell apoptotic process [GO:0033027]; positive regulation of neuron apoptotic process [GO:0043525]; positive regulation of vascular associated smooth muscle cell proliferation [GO:1904707]; protein import into nucleus [GO:0006606]; Rac protein signal transduction [GO:0016601]; Ras protein signal transduction		

						[GO:0007265]; regulation of angiogenesis [GO:0045765]; regulation of blood vessel endothelial cell migration [GO:0043535]; regulation of bone resorption [GO:0045124]; regulation of cell-matrix adhesion [GO:0001952]; regulation of ERK1 and ERK2 cascade [GO:0070372]; regulation of gene expression [GO:0010468]; regulation of glial cell differentiation [GO:0045685]; regulation of GTPase activity [GO:0043087]; regulation of long-term neuronal synaptic plasticity [GO:0048169]; regulation of long-term synaptic potentiation [GO:1900271]; regulation of postsynapse organization [GO:0099175]; regulation of synaptic transmission, GABAergic [GO:0032228]; response to hypoxia [GO:0001666]; Schwann cell development [GO:0014044]; Schwann cell migration [GO:0036135]; Schwann cell proliferation [GO:0014010]; skeletal muscle tissue development [GO:0007519]; smooth muscle tissue development [GO:0048745]; spinal cord development [GO:0021510]; stem cell proliferation [GO:0072089]; sympathetic nervous system development [GO:0048485]; vascular associated smooth muscle cell migration [GO:1904738]; vascular associated smooth muscle cell proliferation [GO:1990874]; visual learning [GO:0008542]; wound healing [GO:0042060]		
BNIP2	663	ENSG000001 40299	Q12982	CRALTRIO	BCL2/adenovirus E1B 19 kDa protein-interacting protein 2	apoptotic process [GO:0006915]; negative regulation of apoptotic process [GO:0043066]; response to oxygen-glucose deprivation [GO:0090649]	cytoplasm [GO:0005737]; cytosol [GO:0005829]; intracellular membrane-bounded organelle [GO:0043231]; nuclear envelope [GO:0005635]; nucleolus [GO:0005730]; perinuclear region of cytoplasm [GO:0048471]	calcium ion binding [GO:0005509]; GTPase activator activity [GO:0005096]
PRUNE2	158471	ENSG000001 06772	Q8WUY3	CRALTRIO	Protein prune homolog 2 (BNIP2 motif-containing molecule at the C-terminal region 1)	apoptotic process [GO:0006915]	cytoplasm [GO:0005737]; cytosol [GO:0005829]	metal ion binding [GO:0046872]; pyrophosphatase activity [GO:0016462]

PTPN9	5780	ENSG0000016 9410	P43378	CRALTRIO	Tyrosine-protein phosphatase non-receptor type 9 (EC 3.1.3.48) (Protein-tyrosine phosphatase MEG2) (PTPase MEG2)	negative regulation of neuron projection development [GO:0010977]; peptidyl-tyrosine dephosphorylation [GO:0035335]; positive regulation of protein localization to plasma membrane [GO:1903078]; protein dephosphorylation [GO:0006470]	cytoplasm [GO:0005737]; neuron projection terminus [GO:0044306]; nucleoplasm [GO:0005654]	non-membrane spanning protein tyrosine phosphatase activity [GO:0004726]; protein tyrosine phosphatase activity [GO:0004725]
MCF2L2	23101	ENSG00000053524	Q86YR7	CRALTRIO	Probable guanine nucleotide exchange factor MCF2L2 (Dbs-related Rho family guanine nucleotide exchange factor) (MCF2-transforming sequence-like protein 2)		cytoplasm [GO:0005737]	guanyl-nucleotide exchange factor activity [GO:0005085]
PDZD8	118987	ENSG0000016 5650	Q8NEN9	SMP	PDZ domain-containing protein 8 (Sarcoma antigen NY-SAR-84/NY-SAR-104)	cytoskeleton organization [GO:0007010]; lipid transport [GO:0006869]; mitochondrial calcium ion homeostasis [GO:0051560]; mitochondrion-endoplasmic reticulum membrane tethering [GO:1990456]; regulation of cell morphogenesis [GO:0022604]	endoplasmic reticulum membrane [GO:0005789]; membrane [GO:0016020]; mitochondria-associated endoplasmic reticulum membrane [GO:0044233]; mitochondrion [GO:0005739]	lipid binding [GO:0008289]; metal ion binding [GO:0046872]
ESYT3	83850	ENSG00000158220	A0FGR9	SMP	Extended synaptotagmin-3 (E-Syt3) (Chr3Syt)	endoplasmic reticulum-plasma membrane tethering [GO:0061817]; lipid transport [GO:0006869]	cytoplasmic side of plasma membrane [GO:0009898]; endoplasmic reticulum membrane [GO:0005789]; endoplasmic reticulum-plasma membrane contact site [GO:0140268]; extrinsic component of cytoplasmic side of plasma membrane [GO:0031234]; organelle membrane contact site [GO:0044232]; plasma membrane [GO:0005886]	calcium ion binding [GO:0005509]; calcium-dependent phospholipid binding [GO:0005544]; phosphatidylcholine binding [GO:0031210]; phosphatidylethanolamine binding [GO:0008429]; phosphatidylinositol binding [GO:0035091]
ESYT1	23344	ENSG00000139641	Q9BSJ8	SMP	Extended synaptotagmin-1 (E-Syt1) (Membrane-bound C2 domain-containing protein)	endoplasmic reticulum-plasma membrane tethering [GO:0061817]; intermembrane lipid transfer [GO:0120009]	endoplasmic reticulum [GO:0005783]; endoplasmic reticulum membrane [GO:0005789]; extrinsic component of cytoplasmic side of plasma membrane [GO:0031234]; membrane [GO:0016020]	calcium ion binding [GO:0005509]; calcium-dependent phospholipid binding [GO:0005544]; identical protein binding [GO:0042802]; phosphatidylcholine binding [GO:0031210]; phosphatidylethanolamine binding [GO:0008429]; phosphatidylinositol binding [GO:0035091]; phospholipid transfer activity [GO:0120014]
C2CD2L	9854	ENSG0000017 2375	O14523	SMP	Phospholipid transfer protein C2CD2L (C2 domain-containing protein 2-like) (C2CD2-like) (Transmembrane protein 24)	positive regulation of insulin secretion involved in cellular response to glucose stimulus [GO:0035774]	cortical endoplasmic reticulum [GO:0032541]; cytoplasmic side of apical plasma membrane [GO:0098592]; endoplasmic reticulum membrane [GO:0005789]; endoplasmic reticulum-plasma membrane contact site [GO:0140268]; plasma membrane [GO:0005886]	insulin binding [GO:0043559]; phosphatidylinositol binding [GO:0035091]; phosphatidylinositol transfer activity [GO:0008526]

C2CD2	25966	ENSG000001 57617	Q9Y426	SMP	C2 domain-containing protein 2 (Transmembrane protein 24-like)		cytosol [GO:0005829]; membrane [GO:0016020]; nucleus [GO:0005634]	
GRAMD1A	57655	ENSG000000893 51	Q96CP6	ASTER/VAST	Protein Aster-A (GRAM domain-containing protein 1A)	autophagy [GO:0006914]; cellular response to cholesterol [GO:0071397]; intracellular sterol transport [GO:0032366]	autophagosome [GO:0005776]; cytoplasmic vesicle [GO:0031410]; cytosol [GO:0005829]; endoplasmic reticulum membrane [GO:0005789]; endoplasmic reticulum-plasma membrane contact site [GO:0140268]; organelle membrane contact site [GO:0044232]; plasma membrane [GO:0005886]	cholesterol binding [GO:0015485]; cholesterol transfer activity [GO:0120020]
GRAMD1B	57476	ENSG000000 23171	Q3KR37	ASTER/VAST	Protein Aster-B (GRAM domain-containing protein 1B)	cellular response to cholesterol [GO:0071397]; cholesterol homeostasis [GO:0042632]; intracellular sterol transport [GO:0032366]	endoplasmic reticulum membrane [GO:0005789]; endoplasmic reticulum-plasma membrane contact site [GO:0140268]; membrane [GO:0016020]; plasma membrane [GO:0005886]	cholesterol binding [GO:0015485]; cholesterol transfer activity [GO:0120020]; phosphatidic acid binding [GO:0070300]; phosphatidylserine binding [GO:0001786]
VPS13C	54832	ENSG00000129003	Q709C8	VPS13	Intermembrane lipid transfer protein VPS13C (Vacuolar protein sorting-associated protein 13C)	Golgi to endosome transport [GO:0006895]; lipid transport [GO:0006869]; mitochondrion organization [GO:0007005]; negative regulation of parkin-mediated stimulation of mitophagy in response to mitochondrial depolarization [GO:1905090]; protein retention in Golgi apparatus [GO:0045053]; protein targeting to vacuole [GO:0006623]; response to insulin [GO:0032868]	cytoplasm [GO:0005737]; cytosol [GO:0005829]; dense core granule membrane [GO:0032127]; endoplasmic reticulum membrane [GO:0005789]; extracellular exosome [GO:0070062]; extrinsic component of membrane [GO:0019898]; late endosome [GO:0005770]; late endosome membrane [GO:0031902]; lipid droplet [GO:0005811]; lysosomal membrane [GO:0005765]; lysosome [GO:0005764]; mitochondrial outer membrane [GO:0005741]	
OSBP	5007	ENSG00000110048	P22059	OSBP	Oxysterol-binding protein 1	bile acid biosynthetic process [GO:0006699]; ceramide transport [GO:0035627]; intracellular cholesterol transport [GO:0032367]; phospholipid transport [GO:0015914]; positive regulation of insulin secretion involved in cellular response to glucose stimulus [GO:0035774]; positive regulation of secretory granule organization [GO:1904411]; positive regulation of tyrosine phosphorylation of STAT protein [GO:0042531]; sphingomyelin biosynthetic process [GO:0006686]; sterol transport [GO:0015918]	cell junction [GO:0030054]; cytoplasm [GO:0005737]; cytosol [GO:0005829]; endoplasmic reticulum membrane [GO:0005789]; Golgi apparatus [GO:0005794]; Golgi membrane [GO:0000139]; membrane [GO:0016020]; nucleolus [GO:0005730]; nucleoplasm [GO:0005654]; perinuclear endoplasmic reticulum [GO:0097038]; perinuclear region of cytoplasm [GO:0048471]; plasma membrane [GO:0005886]; trans-Golgi network [GO:0005802]	oxysterol binding [GO:0008142]; phosphatidylinositol-4-phosphate binding [GO:0070273]; protein domain specific binding [GO:0019904]; sterol binding [GO:0032934]; sterol transfer activity [GO:0120015]; sterol transporter activity [GO:0015248]

OSBPL11	114885	ENSG000001 44909	Q9BXB4	OSBP	Oxysterol-binding protein-related protein 11 (ORP-11) (OSBP-related protein 11)	fat cell differentiation [GO:0045444]; positive regulation of sequestering of triglyceride [GO:0010890]	cytosol [GO:0005829]; Golgi apparatus [GO:0005794]; late endosome membrane [GO:0031902]; membrane [GO:0016020]; nucleoplasm [GO:0005654]	sterol binding [GO:0032934]; sterol transporter activity [GO:0015248]
STAR	6770	ENSG00000147465	P49675	START	Steroidogenic acute regulatory protein, mitochondrial (StAR) (START domain-containing protein 1) (StARD1)	cellular lipid metabolic process [GO:0044255]; cholesterol metabolic process [GO:0008203]; glucocorticoid metabolic process [GO:0008211]; intracellular cholesterol transport [GO:0032367]; positive regulation of bile acid biosynthetic process [GO:0070859]; regulation of steroid biosynthetic process [GO:0050810]; steroid biosynthetic process [GO:0006694]	mitochondrial intermembrane space [GO:0005758]; mitochondrial matrix [GO:0005759]	cholesterol binding [GO:0015485]; cholesterol transfer activity [GO:0120020]
PCTP	58488	ENSG0000014 1179	Q9UKL6	START	Phosphatidylcholine transfer protein (PC-TP) (START domain-containing protein 2) (StARD2) (StAR-related lipid transfer protein 2)	lipid transport [GO:0006869]; negative regulation of cold-induced thermogenesis [GO:0120163]; phospholipid transport [GO:0015914]	cytosol [GO:0005829]	phosphatidylcholine binding [GO:0031210]; phosphatidylcholine transporter activity [GO:0008525]
ARHGAP8	23779	ENSG0000024 1484	P85298	CRALTRIO	Rho GTPase-activating protein 8 (Rho-type GTPase-activating protein 8)	negative regulation of endocytic recycling [GO:2001136]; positive regulation of ERK1 and ERK2 cascade [GO:0070374]; regulation of small GTPase mediated signal transduction [GO:0051056]; small GTPase-mediated signal transduction [GO:0007264]	cytoplasm [GO:0005737]; cytosol [GO:0005829]	GTPase activator activity [GO:0005096]
ARHGAP1	392	ENSG00000175220	Q07960	CRALTRIO	Rho GTPase-activating protein 1 (CDC42 GTPase-activating protein) (GTPase-activating protein rhoGAP) (Rho-related small GTPase protein activator) (Rho-type GTPase-activating protein 1) (p50-RhoGAP)	endosomal transport [GO:0016197]; negative regulation of endocytic recycling [GO:2001136]; regulation of small GTPase mediated signal transduction [GO:0051056]; Rho protein signal transduction [GO:0007266]; small GTPase-mediated signal transduction [GO:0007264]; transferrin transport [GO:0033572]	cytoplasm [GO:0005737]; cytosol [GO:0005829]; endosome membrane [GO:0010008]; extracellular exosome [GO:0070062]; perinuclear region of cytoplasm [GO:0048471]	cadherin binding [GO:0045296]; GTPase activator activity [GO:0005096]; SH3 domain binding [GO:0017124]; small GTPase binding [GO:0031267]
BNIPL	149428	ENSG000001 63141	Q7Z465	CRALTRIO	Bcl-2/adenovirus E1B 19 kDa-interacting protein 2-like protein	apoptotic process [GO:0006915]; negative regulation of cell population proliferation [GO:0008285]; regulation of growth rate [GO:0040009]	cytoplasm [GO:0005737]; cytosol [GO:0005829]; nucleus [GO:0005634]	identical protein binding [GO:0042802]

ATCAY	85300	ENSG0000016 7654	Q86WG3	CRALTRIO	Caytaxin (Ataxia cayman type protein) (BNIP-2-homology) (BNIP-H)	apoptotic process [GO:0006915]; mitochondrion distribution [GO:0048311]; negative regulation of glutamate metabolic process [GO:2000212]; neuron projection development [GO:0031175]; regulation of protein localization [GO:0032880]	axon [GO:0030424]; cytoplasm [GO:0005737]; dendrite [GO:0030425]; growth cone [GO:0030426]; mitochondrial membrane [GO:0031966]; neuron projection [GO:0043005]; presynapse [GO:0098793]; synapse [GO:0045202]	kinesin binding [GO:0019894]
TRIO	7204	ENSG00000038382	O75962	CRALTRIO	Triple functional domain protein (EC 2.7.11.1) (PTPRF-interacting protein)	axon guidance [GO:0007411]; negative regulation of fat cell differentiation [GO:0045599]; neuron projection morphogenesis [GO:0048812]; phosphorylation [GO:0016310]; postsynaptic modulation of chemical synaptic transmission [GO:0099170]; regulation of small GTPase mediated signal transduction [GO:0051056]; transmembrane receptor protein tyrosine phosphatase signaling pathway [GO:0007185]	cell projection [GO:0042995]; cytoplasm [GO:0005737]; cytosol [GO:0005829]; extrinsic component of membrane [GO:0019898]; glutamatergic synapse [GO:0098978]; postsynapse [GO:0098794]; presynaptic active zone [GO:0048786]	ATP binding [GO:0005524]; guanyl-nucleotide exchange factor activity [GO:0005085]; protein serine kinase activity [GO:0106310]; protein serine/threonine kinase activity [GO:0004674]
KIAA1755	85449	ENSG000001 49633	Q5JYT7	CRALTRIO	Uncharacterized protein KIAA1755	axon guidance [GO:0007411]	cytoplasm [GO:0005737]; extrinsic component of membrane [GO:0019898]; plasma membrane [GO:0005886]	guanyl-nucleotide exchange factor activity [GO:0005085]
KALRN	8997	ENSG00000160145	O60229	CRALTRIO	Kalirin (EC 2.7.11.1) (Huntingtin-associated protein-interacting protein) (Protein Duo) (Serine/threonine-protein kinase with Dbl- and pleckstrin homology domain)	axon guidance [GO:0007411]; ephrin receptor signaling pathway [GO:0048013]; intracellular signal transduction [GO:0035556]; nervous system development [GO:0007399]; protein phosphorylation [GO:0006468]; regulation of small GTPase mediated signal transduction [GO:0051056]; signal transduction [GO:0007165]; vesicle-mediated transport [GO:0016192]	actin cytoskeleton [GO:0015629]; cytoplasm [GO:0005737]; cytosol [GO:0005829]; extracellular exosome [GO:0070062]; extrinsic component of membrane [GO:0019898]; nucleoplasm [GO:0005654]; postsynaptic density [GO:0014069]	ATP binding [GO:0005524]; guanyl-nucleotide exchange factor activity [GO:0005085]; metal ion binding [GO:0046872]; protein serine kinase activity [GO:0106310]; protein serine/threonine kinase activity [GO:0004674]
ESYT2	57488	ENSG00000117868	A0FGR8	SMP	Extended synaptotagmin-2 (E-Syt2) (Chr2Syt)	endocytosis [GO:0006897]; endoplasmic reticulum-plasma membrane tethering [GO:0061817]; lipid transport [GO:0006869]	cytoplasmic side of plasma membrane [GO:0009898]; cytosol [GO:0005829]; endoplasmic reticulum membrane [GO:0005789]; endoplasmic reticulum-plasma membrane contact site [GO:0140268]; extrinsic component of cytoplasmic side of plasma membrane [GO:0031234]; membrane [GO:0016020]; organelle membrane contact site [GO:0044232]; plasma membrane [GO:0005886]	cadherin binding [GO:0045296]; calcium ion binding [GO:0005509]; calcium-dependent phospholipid binding [GO:0005544]; identical protein binding [GO:0042802]; phosphatidylcholine binding [GO:0031210]; phosphatidylethanolamine binding [GO:0008429]; phosphatidylinositol binding [GO:0035091]
GRAMD1C	54762	ENSG000001 78075	Q8IYS0	ASTER/VAST	Protein Aster-C (GRAM domain-containing protein 1C)	cellular response to cholesterol [GO:0071397]; intracellular sterol transport [GO:0032366]	endoplasmic reticulum membrane [GO:0005789]; endoplasmic reticulum-plasma membrane contact site [GO:0140268]; plasma membrane [GO:0005886]	cholesterol binding [GO:0015485]; cholesterol transfer activity [GO:0120020]

VPS13A	23230	ENSG00000197969	Q96RL7	VPS13	Intermembrane lipid transfer protein VPS13A (Chorea-acanthocytosis protein) (Chorein) (Vacuolar protein sorting-associated protein 13A)	autophagy [GO:0006914]; flagellated sperm motility [GO:0030317]; Golgi to endosome transport [GO:0006895]; lipid transport [GO:0006869]; locomotory behavior [GO:0007626]; lysosomal protein catabolic process [GO:1905146]; nervous system development [GO:0007399]; protein localization [GO:0008104]; protein retention in Golgi apparatus [GO:0045053]; protein targeting to vacuole [GO:0006623]; social behavior [GO:0035176]; sperm mitochondrion organization [GO:0030382]	cytosol [GO:0005829]; endoplasmic reticulum membrane [GO:0005789]; endosome membrane [GO:0010008]; extrinsic component of membrane [GO:0019898]; Golgi apparatus [GO:0005794]; lipid droplet [GO:0005811]; lysosomal membrane [GO:0005765]; mitochondria-associated endoplasmic reticulum membrane [GO:0044233]; mitochondrial membrane [GO:0031966]; mitochondrial outer membrane [GO:0005741]; neuronal dense core vesicle lumen [GO:0099013]; sperm midpiece [GO:0097225]	
VPS13B	157680	ENSG00000132549	Q7Z7G8	VPS13	Intermembrane lipid transfer protein VPS13B (Cohen syndrome protein 1) (Vacuolar protein sorting-associated protein 13B)	acrosome assembly [GO:0001675]; adipose tissue development [GO:0060612]; central nervous system development [GO:0007417]; Golgi organization [GO:0007030]; Golgi reassembly [GO:0090168]; lipid transport [GO:0006869]; maintenance of lens transparency [GO:0036438]; nervous system development [GO:0007399]; neuron projection development [GO:0031175]; slow endocytic recycling [GO:0032458]; vesicle-mediated transport [GO:0016192]	acrosomal membrane [GO:0002080]; cis-Golgi network membrane [GO:0033106]; early endosome membrane [GO:0031901]; endoplasmic reticulum-Golgi intermediate compartment membrane [GO:0033116]; Golgi membrane [GO:0000139]; lysosomal membrane [GO:0005765]; recycling endosome membrane [GO:0055038]; trans-Golgi network membrane [GO:0032588]	phosphatidylinositol-3-phosphate binding [GO:0032266]

Table S3. The human tumor cell lines used by National Cancer Institute as a screen to characterize novel compounds for tumor treatment. The screen utilizes 60 cell lines, representing leukemia (LE, n=6), melanoma (ME, n=10), and cancers of breast (BR, n=5), central nervous system/brain (CNS, n=6), colon (CO, n=7), non-small cell lung (LC, n=9), ovarian (OV, n=7), prostate (PR, n=2) and renal (RE, n=8).

Cell line	Cancer	age	sex	prior treatment	Epithelial	histology	source	ploidy	doubling time	Reference
BR:MCF7	Breast	69	F		yes	Adenocarcinoma- mammary gland; breast; metastatic site: pleural effusion;	Pleural effusion	3n-, Hypotriploid (58-68)	25.4	JNCI 51: 1409-1417,1973
BR:MDA-MB-231	Breast	51	F		yes	Adenocarcinoma-mammary gland; breast; epithelial; metastatic site: pleural effusion;	Pleural effusion	2n+, Hyperdiploid (47-57)	41.9	JNCI 53(3): 661-674,1974
BR:HS 578T	Breast	74	F		yes	Carcinosarcoma-mammary gland; breast	Primary	2n+, Hyperdiploid (47-57)	53.8	JNCI 58: 1795-1806,1977
BR:BT-549	Breast	72	F		yes	Papillary infiltrating ductal carcinoma-mammary gland; breast	Metastasis	3n+/-, Near-triploid 69+/- (58-80)	53.9	no pub
BR:T-47D	Breast	54	F		yes	infiltrating ductal carcinoma		2n+, Hyperdiploid (47-57)	45.5	Eur J Cancer 15:659-679,1979
CNS:SF-268	Central nervous system	24	F	Rad	no	Glioblastoma, ud		2n+, Hyperdiploid (47-57)	33.1	Acta Neuropathol 75: 92-103, 1987
CNS:SF-295	Central nervous system	67	F	Rad	no	Glioblastoma, ud		5n+/-, Near-pentaploid 115+/- (104-126)	29.5	Acta Neuropathol 75: 92-103, 1988
CNS:SF-539	Central nervous system	34	F	Rad/BCNU/5FU/HU/6MP	no	Glial cell neoplasm		4n+/-, Near-tetraploid 92+/- (81-103)	35.4	J Neuropathol Exp Neurol 40: 201-229, 1981
CNS:SNB-19	Central nervous system	47	M		no	Glioblastoma, ud		3n+/-, Near-triploid 69+/- (58-80)	34.6	Cancer 47: 255, 1981
CNS:SNB-75	Central nervous system		F		no	Astrocytoma		2n+, Hyperdiploid (47-57)	62.8	?
CNS:U251	Central nervous system	75	M		no	Glioblastoma,ud		2n+, Hyperdiploid (47-57)	23.8	J Neuropathol Exp Neurol 40: 410-427, 1981
CO:COLO 205	Colon	70	M		yes	Adenocarcinoma	Ascites	3n, Triploid (69)	23.8	Can Res. 38: 1345-1355, 1978
CO:HCC-2998	Colon				yes	carcinoma		2n+/-, Near-diploid 46+/- (35-57)	31.5	?
CO:HCT-116	Colon		M		yes	carcinoma-vpd		2n-, Hypodiploid (35-45)	17.4	Can Res 41: 1761-1756, 1981

CO:HCT-15	Colon				yes	Adenocarcinoma p/md		2n+/-, Near-diploid 46+/- (35-57)	20.6	Can Res 39: 1020-1025, 1979
CO:HT29	Colon	44	F		yes	Adenocarcinoma-md	Primary	3n+/-, Near-triploid 69+/- (58-80)	19.5	Human Tumor Cells in vitro: 115-159, 1975
CO:KM12	Colon				yes	Adenocarcinome-pd		2n+/-, Near-diploid 46+/- (35-57)	23.7	Can Res 48: 1943-1948, 1988
CO:SW-620	Colon	51	M		yes	Carcinoma-ud		2n+/-, Near-diploid 46+/- (35-57)	20.4	Can Res 1976 Dec;36(12):4562-9
LE:CCRF-CEM	Leukemia	4	F	Rad/Mtx/Ctx	no	ALL		2n+/-, Near-diploid 46+/- (35-57)	26.7	Cancer 18: 522-529, 1965
LE:HL-60(TB)	Leukemia	36	F		no	Pro myelocytic leukemia	PBL	2n+/-, Near-diploid 46+/- (35-57)	28.6	Blood 54(3):713-33, 1979
LE:K-562	Leukemia	53	F	Bisulfan/PiBr	no	CML	Pleural effusion	3n-, Hypotriploid (58-68)	19.6	Blood 45: 321-334, 1975
LE:MOLT-4	Leukemia	19	M	VB/6MP/Pred	no	ALL (cells were taken when patient was in relapse)	PB	4n, Tetraploid (92)	27.9	J. Natl. Cancer Inst. 49: 891-895, 1972
LE:RPMI-8226	Leukemia	61	M		no	Myeloma	PB	3n-, Hypotriploid (58-68)	33.5	Proc Soc Exp Biol Med 125: 1246-1250, 1967
LE:SR	Leukemia	11	M		no	Lymphoma		2n+/-, Near-diploid 46+/- (35-57)	28.7	?
ME:LOX IMVI	Melanoma	58	M		no	Malignant amelanotic melanoma		3n+/-, Near-triploid 69+/- (58-80)	20.5	Int J Cancer 41: 442-449, 1988
ME:MALME-3M	Melanoma	43	M		no	Malignant melanotic melanoma	Metastasis	4n+/-, Near-tetraploid 92+/- (81-103)	46.2	J. Natl. Cancer Inst. 59: 221-226, 1977
ME:M14	Melanoma				no	Melanotic melanoma		3n+/-, Near-triploid 69+/- (58-80)	26.3	Cancer Res 48: 578-582, 1988
ME:SK-MEL-2	Melanoma	60	M		no	Malignant melanotic melanoma	Metastasis	4n-, Hypotetraploid (81-91)	45.5	Human Tumor Cells in vitro, pp 115-159, 1975
ME:SK-MEL-28	Melanoma	51	M		no	Malignant melanotic melanoma		4n-, Hypotetraploid (81-91)	35.1	PNAS 73: 3278-3282, 1976
ME:SK-MEL-5	Melanoma	24	F		no	Malignant melanotic melanoma	Metastasis	4n+, Hypertetraploid (93-103)	25.2	PNAS, 73: 3278-3282, 1976
ME:UACC-257	Melanoma				no	Melanotic melanoma		3n+, Hypertriploid (70-80)	38.5	?
ME:UACC-62	Melanoma				no	Melanotic melanoma		3n+/-, Near-triploid 69+/- (58-80)	31.3	?
ME:MDA-MB-435	Melanoma	31	F		no	Ductal carcinoma- mammary gland; breast; duct; metastatic site: pleural effusion;	Pleural effusion	2n+, Hyperdiploid (47-57)	25.8	Cancer Res 40:3118-3129,1980
LC:A549/ATCC	Non-Small Cell Lung	58	M		yes	Adenocarcinoma-p/md		3n+/-, Near-triploid 69+/- (58-80)	22.9	J Natl Cancer Inst. 51(5):1417-23, 1973

LC:EKVX	Non-Small Cell Lung		M		yes	Adenocarcinoma-md		3n+/-, Near-triploid 69+/- (58-80)	43.6	?
LC:HOP-62	Non-Small Cell Lung	60	F		yes	adenocarcinoma-ud		4n+, Hypertetraploid (93-103)	39	?
LC:HOP-92	Non-Small Cell Lung	62	M		yes	Large cell-ud		4n+/-, Near-tetraploid 92+/- (81-103)	79.5	?
LC:NCI-H226	Non-Small Cell Lung		M	None (non smoker)	yes	Squamous cell carcinoma-vpd		3n, Triploid (69)	61	Cancer Res. 40: 3502-3507, 1980
LC:NCI-H23	Non-Small Cell Lung		M		yes	Adenocarcinoma-ud		2n+, Hyperdiploid (47-57)	33.4	Cancer Res. 40: 3502-3507, 1980
LC:NCI-H322M	Non-Small Cell Lung	52	M		yes	Small cell Bronchioalveolar Carcinoma		2n+/-, Near-diploid 46+/- (35-57)	35.3	?
LC:NCI-H460	Non-Small Cell Lung		M		yes	Large Cell Carcinoma-ud	Pleural effusion	2n+/-, Near-diploid 46+/- (35-57)	17.8	Science 246: 491-494, 1989
LC:NCI-H522	Non-Small Cell Lung		M		yes	Adenocarcinoma-vpd		2n+/-, Near-diploid 46+/- (35-57)	38.2	Can Res 45: 2913-2923, 1985
OV:IGROV1	Ovarian	47	F	Rad	yes	Cystoadenocarcinoma-pd		4n+/-, Near-tetraploid 92+/- (81-103)	31	Can Res 45: 4970-4979,1985
OV:OVCAR-3	Ovarian	60	F	CyPh/CsPt/Adr	yes	Adenocarcinoma-md	Ascites	3n+/-, Near-triploid 69+/- (58-80)	34.7	Sem Ocol 11: 285-298, 1984
OV:OVCAR-4	Ovarian	42	F	CyPh/CsPt/Adr	yes	Adenocarcinoma-md		3n+/-, Near-triploid 69+/- (58-80)	41.4	Sem Ocol 11: 285-298, 1984
OV:OVCAR-5	Ovarian	67	F		yes	Adenocarcinoma-wd		2n+, Hyperdiploid (47-57)	48.8	Sem Ocol 11: 285-298, 1985
OV:OVCAR-8	Ovarian	64	F	Ctx/Adr/CsPt/CyPh	yes	Carcinoma-ud		2n+, Hyperdiploid (47-57)	26.1	?
OV:SK-OV-3	Ovarian	64	F	Thiotepa	yes	Adenocarcinoma-vpd	Ascites	4n+/-, Near-tetraploid 92+/- (81-103)	48.7	Human Tumor Cells in vitro, pp. 115-159, 1975
OV:NCI/ADR-RES	Ovarian		F		yes	Adenocarinoma		2n+/-, Near-diploid 46+/- (35-57)	34	Cancer Res., 46:4087-4090,1986
PR:PC-3	Prostate	62	M		yes	Adenocarcinoma- prostate; metastatic site: bone;		4n, Tetraploid (92)	27.1	Invest Urol 1979 Jul;17(1):16-23
PR:DU-145	Prostate	69	M	Androgen independent and unresposive to hormone therapy	yes	prostate; metastatic site: brain; carcinoma (patient with metastatic carcinoma of the prostate and a 3 year history of lymphocytic leukemia.)	Metastasis	3n+/-, Near-triploid 69+/- (58-80)	32.3	Int J Cancer 21: 274-281,1978
RE:786-0	Renal	58	M		yes	Adenocarcinoma		4n+/-, Near-tetraploid 92+/- (81-103)	22.4	In Vitro 14: 779-786, 1978

RE:A498	Renal	52	F		yes	Adenocarcinoma		3n, Triploid (69)	66.8	JNCI 51: 1417-1423,1973
RE:ACHN	Renal	22	M	Rad/VB/CCNU/Mto/Pred	yes	Renal cell carcinoma-p/md		2n+/-, Near-diploid 46+/- (35-57)	27.5	Cancer Res 42: 4948-4953, 1982
RE:CAKI-1	Renal	49	M	Rad/HU/5FU/Mtx/Ctx	yes	Clear cell carcinoma	Metastasis	3n, Triploid (69)	39	Human Tumor cells in vitro, pp 115-159, 1975
RE:RXF 393	Renal	54	M		yes	hypernephroma-pd		3n+/-, Near-triploid 69+/- (58-80)	62.9	Contrib oncol 42, 1992
RE:SN12C	Renal	43	M		yes	Renal cell carcinoma-pd		3n, Triploid (69)	29.5	Cancer Res 46: 4109-4115, 1986
RE:TK-10	Renal	43	M		yes	Renal Spindle cell carcinoma		4n, Tetraploid (92)	51.3	Cancer Res 46: 3856-3862, 1987
RE:UO-31	Renal		F		yes	Renal cell carcinoma-vpd		2n+/-, Near-diploid 46+/- (35-57)	41.7	?

Table S4. LTP family and gene knockout along with the cluster assigned to the genes by k-nearest neighbors. The mismatch between the clusters and LTP family suggests that examination on a gene-by-gene basis is necessary to group genes functionally in way corresponding to their actual effect on the lipidome.

LTP Family	Target Gene Name	k cluster
NPC1 NTD	NPC1	1
ML	NPC2	1
START	STARD7	1
OSBP	OSBPL9	1
START	STARD6	1
CRALTRIO	PTPN9	1
START	STARD4	1
Non_target	NonTarget_1	1
CRALTRIO	SEC14L2	1
CRALTRIO	MOSPD2	1
Non_target	NonTarget_4	1
PITP	PITPNB	1
PITP	PITPNA	1
SCP2	SCP2	1
CRALTRIO	PTPN9	1
PITP	PITPNB	1
GLTP	GLTPD2	1
CRALTRIO	MOSPD2	1
NPC1 NTD	NPC1	1
CRALTRIO	SEC14L1	1
CRALTRIO	ARHGAP8	1
GLTP	GLTP	1
Non_target	NonTarget_2	1
Non_target	NonTarget_3	1
Non_target	NonTarget_3	1
VPS13	VPS13A	1
OSBP	OSBP	1
CRALTRIO	ARHGAP8	1
Non_target	NonTarget_4	1
Non_target	NonTarget_5	1
OSBP	OSBPL9	1
OSBP	OSBPL8	1
CRALTRIO	SEC14L3	1
VPS13	VPS13D	2
Non_target	NonTarget_6	2
SCP2	SCP2	2
OSBP	OSBPL8	2
CRALTRIO	SEC14L1	2
OSBP	OSBPL9	2
NPC1 NTD	NPC1	2
START	STARD7	2
OSBP	OSBPL1A	2
VPS13	VPS13A	2
SCP2	SCP2	2
Non_target	NonTarget_6	2
ML	LY96	2
SCP2	SCP2D1	2
Non_target	NonTarget_2	2
OSBP	OSBPL5	2
GLTP	GLTPD2	2
CRALTRIO	MCF2	2
CRALTRIO	RLBP1	2
OSBP	OSBPL10	2
ML	LY86	2
Non_target	NonTarget_3	2
Non_target	NonTarget_4	2
CRALTRIO	CLVS2	2

CRALTRIO	GDAP2	2
START	STARD3	2
OSBP	OSBPL6	2
OSBP	OSBPL7	2
ML	GM2A	2
PITP	PITPNM2	2
PITP	PITPNM1	2
PITP	PITPNB	2
SCP2	HSDL2	2
GLTP	CPTP	2
CRALTRIO	MCF2L	2
CRALTRIO	CLVS1	2
CRALTRIO	TTPAL	2
CRALTRIO	MOSPD2	2
CRALTRIO	KALRN	2
PITP	PITPNM3	2
ASTER/VAST	GRAMD1C	2
Non_target	NonTarget_4	2
NPC1 NTD	NPC1L1	2
SMP	TEX2	2
ASTER/VAST	GRAMD1A	2
SMP	ESYT1	2
SMP	C2CD2	2
CRALTRIO	SESTD1	2
ASTER/VAST	GRAMD1B	2
CRALTRIO	PRUNE2	2
SMP	C2CD2L	2
OSBP	OSBPL11	3
CRALTRIO	TRIO	3
SMP	TEX2	3
GLTP	PLEKHA8	3
PITP	PITPNC1	3
START	STARD10	3
CRALTRIO	TTPA	3
CRALTRIO	ATCAY	3
CRALTRIO	BNIPL	3
SMP	ESYT2	3
START	STAR	3
START	PCTP	3
VPS13	VPS13B	3
Non_target	NonTarget_2	3
CRALTRIO	KIAA1755	3
CRALTRIO	KALRN	3
START	STARD13	3
OSBP	OSBPL2	3
CRALTRIO	TTPA	3
START	COL4A3BP	3
CRALTRIO	MOSPD2	3
SMP	ESYT3	3
START	ACOT12	3
Non_target	NonTarget_5	3
START	STARD5	3
OSBP	OSBP2	3
START	STARD6	3
START	STARD8	3
START	STARD9	3
Non_target	NonTarget_5	3
Non_target	NonTarget_6	3
START	DLC1	3
Non_target	NonTarget_2	3
START	STARD4	3
GLTP	GLTP	3
SCP2	STOML1	3
CRALTRIO	ARHGAP1	3
ML	NPC2	3
Non_target	NonTarget_3	3

Non_target	NonTarget_4	3
CRALTRIO	SEC14L4	3
SMP	PDZD8	3
START	ACOT11	3
PITP	PITPNA	3
VPS13	VPS13D	3
CRALTRIO	SEC14L5	3
CRALTRIO	SEC14L6	3
OSBP	OSBPL1A	3
OSBP	OSBPL3	3
START	STARD9	3
PITP	PITPNC1	3
CRALTRIO	NF1	3
CRALTRIO	PTPN9	3
CRALTRIO	MCF2L2	3
CRALTRIO	SEC14L2	3
CRALTRIO	SEC14L3	3
CRALTRIO	BNIP2	3
VPS13	VPS13C	3
OSBP	OSBP	3
CRALTRIO	SEC14L1	3
OSBP	OSBPL11	3
ASTER/VAST	GRAMD1C	3
START	COL4A3BP	3
GLTP	GLTPD2	3
ASTER/VAST	GRAMD1C	3
ASTER/VAST	GRAMD1C	3
Non_target	NonTarget_5	3
CRALTRIO	ARHGAP8	3
VPS13	VPS13B	3
Non_target	NonTarget_2	3
Non_target	NonTarget_3	3
NPC1 NTD	NPC1	4
ML	NPC2	4
CRALTRIO	SESTD1	4
CRALTRIO	GDAP2	4
Non_target	NonTarget_1	4
Non_target	NonTarget_5	4
OSBP	OSBP	4
PITP	PITPNA	4
SCP2	SCP2	5
Non_target	NonTarget_6	5
START	STARD8	5
Non_target	NonTarget_5	5
START	STARD3	5
CRALTRIO	NF1	5
ASTER/VAST	GRAMD1B	5
START	STARD4	5
NPC1 NTD	NPC1L1	5
GLTP	PLEKHA8	5
Non_target	NonTarget_6	5
OSBP	OSBPL9	5
ASTER/VAST	GRAMD1A	5
START	COL4A3BP	5
CRALTRIO	TRIO	5
SMP	C2CD2	5
CRALTRIO	MOSPD2	5
Non_target	NonTarget_1	5
START	STARD5	5
CRALTRIO	CLVS1	5
Non_target	NonTarget_2	5
CRALTRIO	RLBP1	5
Non_target	NonTarget_4	5
CRALTRIO	SEC14L5	5
Non_target	NonTarget_3	5
START	STAR	5

START	STARD13	5
START	STARD10	5
START	DLC1	5
CRALTRIO	TTPA	5
CRALTRIO	BNIPL	5
CRALTRIO	PTPN9	5
Non_target	NonTarget_6	5
CRALTRIO	GDAP2	5
PITP	PITPNM3	5
SCP2	SCP2D1	5
CRALTRIO	SESTD1	5
CRALTRIO	CLVS2	5
VPS13	VPS13D	5
CRALTRIO	MCF2L2	5
START	PCTP	5
ML	LY86	5
ML	GM2A	5
ML	LY96	5
CRALTRIO	SEC14L6	5
CRALTRIO	MCF2	5
SCP2	HSD17B4	5
SCP2	HSD17B4	5
CRALTRIO	ATCAY	5
VPS13	VPS13C	5
GLTP	GLTP	5
SMP	ESYT1	5
OSBP	OSBPL2	5
SMP	ESYT2	5
SMP	ESYT3	5
CRALTRIO	SEC14L2	5
CRALTRIO	SEC14L3	5
START	STARD6	5
OSBP	OSBPL10	5
START	ACOT11	5
PITP	PITPNM1	5
SCP2	STOML1	5
OSBP	OSBPL5	5
VPS13	VPS13B	5
OSBP	OSBPL11	5
PITP	PITPNB	5
CRALTRIO	TTPAL	5
CRALTRIO	SEC14L1	5
PITP	PITPNM2	5
CRALTRIO	SEC14L4	5
GLTP	GLTPD2	5
CRALTRIO	PRUNE2	5
SCP2	HSDL2	5
START	STARD9	5
CRALTRIO	MCF2L	5
START	ACOT12	5
PITP	PITPNC1	5
GLTP	CPTP	5
SMP	TEX2	5
OSBP	OSBPL6	5
OSBP	OSBPL1A	5
OSBP	OSBP2	5
OSBP	OSBPL3	5
OSBP	OSBPL7	5
OSBP	OSBPL8	5
VPS13	VPS13A	5
SMP	PDZD8	5
CRALTRIO	ARHGAP8	5
Non_target	NonTarget_5	5
SMP	C2CD2L	5
CRALTRIO	ARHGAP1	5
CRALTRIO	KALRN	5

CRALTRIO	BNIP2	5
CRALTRIO	KIAA1755	5

Table S5. Performance testing of iSODA. The tests were performed on a Windows 10 computer with an Intel W-2135 processor and 64GB RAM.

Dataset	Omics type	# samples	# features	Matrix size [entry]	Size [MB]	Time to upload [sec]	Time to generate volcano plot [sec]	Time to perform SNF [sec]	Time to perform MOFA [sec]
**LTP**	Lipidomics	316	1,107	349,812	4.19	0.3	0.02	NA	NA
**NCI-60**	Proteomics	59	3,013	177,767	1.07	0.33	0.67	0.08	41.59
**NCI-60**	Transcriptomics	59	24,190	1,427,210	8.23	0.47	9.77		
**NCI-60**	Genomics-methylation	59	17,552	1,035,568	5.92	0.44	5.49		
**NDD**	Proteomics	363	1,253	454,839	2.69	0.13	0.44	0.15	55.4
**NDD**	Transcriptomics	363	60,583	21,991,629	106	6.78	28.44		
**NDD**	Metabolomics	363	732	265,716	4.21	0.36	0.85		

Example of combining modular functionalities for novel analysis

In this example we elaborate on the modular design aspect of iSDOA and how it allows to combine analyses. To demonstrate this, we ran enrichment analysis on lipid species annotation regarding saturation. Although enrichment analysis and lipid annotation are both not novel per se, the combination of both in iSODA is enabled by the modular design. Identifying if specific double bonds are enriched can pinpoint significant patterns that are not visible otherwise. For example, saturation enrichment analysis on lipid transfer proteins STARD7 and STARD8 knockouts (in the LTP dataset), produces clear results: although these two LTPs are from the same family, double bonds in STARD8 TG are increased while saturation in smaller non-TG species is decreased (CE, DG, PA, PG, PS). These statistically significant patterns can be visually represented in the CNET of the enrichment analysis (see Figure S10). One can color the nodes according to the fold change and immediately see a pattern of up and down regulation as in Figure S10. STARD7 is known to play a critical role in mitochondrial membrane integrity and function, this is possibly why it seems to transport more saturated lipids. STARD8 on the other hand is mainly involved in signaling, that might be an explanation for having a more diverse cargo including unsaturated lipids. Performing same analysis with different comparisons, one can build patterns of the different genes. For example, comparing STARD8 to control (non-target) and enrich for saturation, we can see that specifically high unsaturation of 7 double bonds of TGs are increased in STARD8, while saturated lipid species are degreased (see Figure S11).

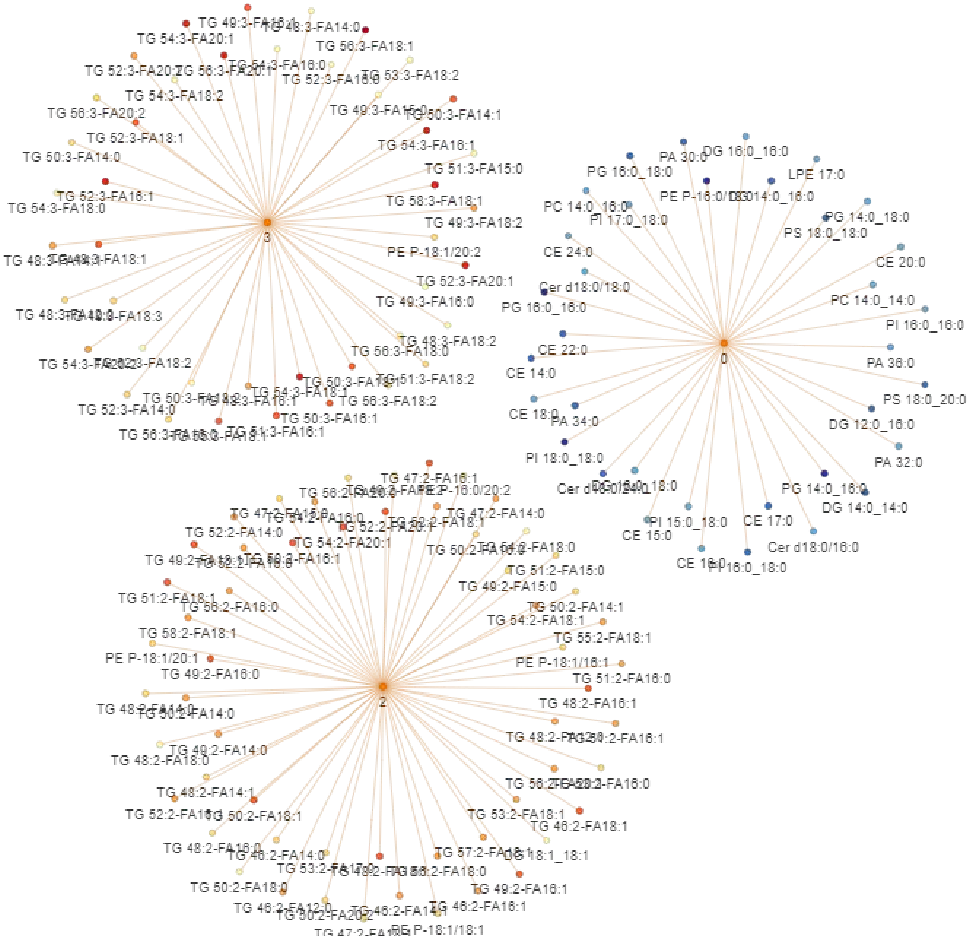

Figure S10. CNET of enrichment analysis on double bonds comparing STRAD8 to STRAD7 lipidomics. The color of the nodes reflects log2 fold change with red positive and blue negative.

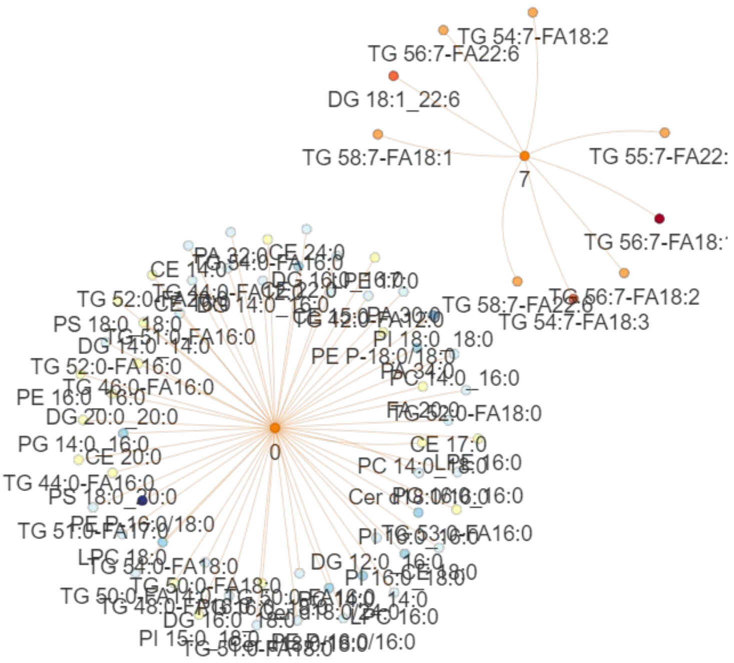

Figure S11. CNET of enrichment analysis on double bonds comparing STRAD8 to control/non-target lipidomics. The color of the nodes reflects log2 fold change with red positive and blue negative.

In summary, this demonstrates how modular design enables the combination of various methods for novel type of analyses in iSODA. This novelty is not because of one single new type of analysis or new implementation, rather it is because of the possibility of bringing different existing methods together.
